# Supplementary material for: Enhancing stability by trapping palladium inside N-heterocyclic carbene-functionalized hypercrosslinked polymers for heterogeneous C-C bond formations
Source: Nat Commun. 2021 Mar 25;12:1875. doi: 10.1038/s41467-021-22084-5 (PMC7994585; doi:10.1038/s41467-021-22084-5)
Supplement: Supplementary file 1 — Supplementary Information [file 41467_2021_22084_MOESM1_ESM.pdf]

Supplementary Information

Enhancing stability by trapping palladium inside N-heterocyclic carbene-functionalized hypercrosslinked polymers for heterogeneous C-C bond formations

Yue et al.

Supplementary Figures

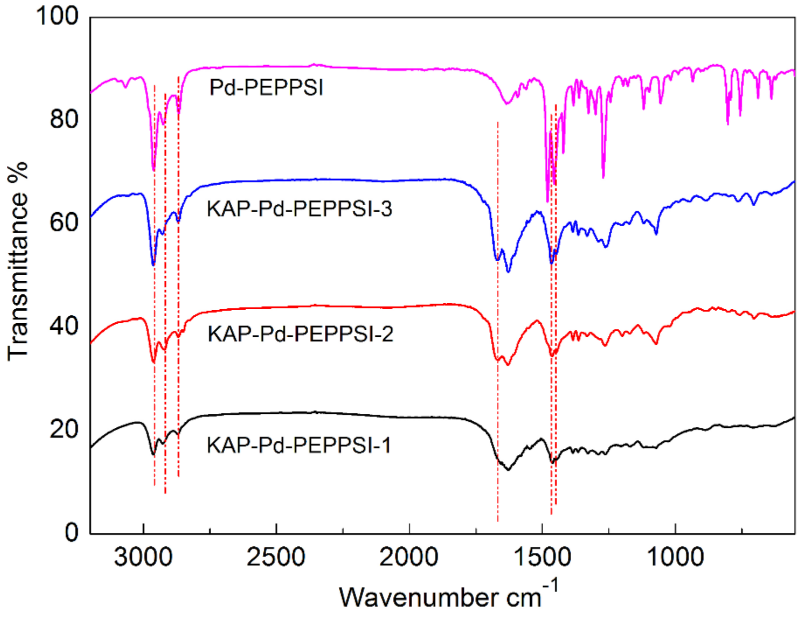

Supplementary Figure 1. FT-TR spectrum of KAP-Pd-PEPPSI. The characteristic peaks are labelled in the spectra (from left to right: 2959, 2918, 2867, 1667, 1465 and 1447 cm^{-1}).

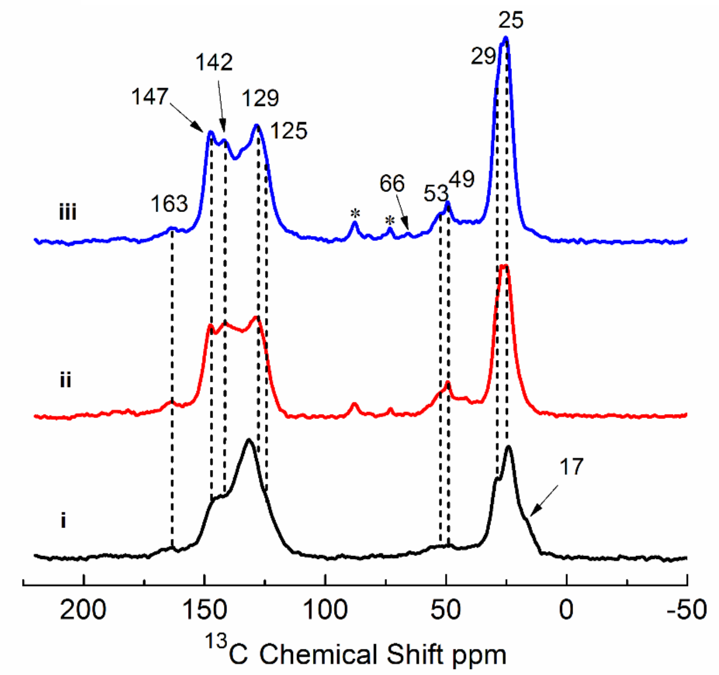

Supplementary Figure 2. Solid-state ^{13}C CP/MAS NMR spectrum of KAP-Pd-PEPPSI. The characteristic peaks are labelled in the spectra. i KAP-Pd-PEPPSI-1, ii KAP-Pd-PEPPSI-2, iii KAP-Pd-PEPPSI-3.

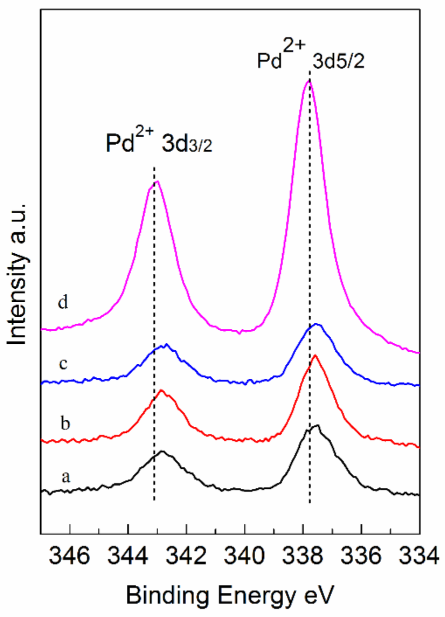

Supplementary Figure 3. Pd 3d XPS spectra of KAP-Pd-PEPPSI. (a) KAP-Pd-PEPPSI-1, (b) KAP-Pd-PEPPSI-2, (c) KAP-Pd-PEPPSI-3, and (d) molecular Pd-PEPPSI.

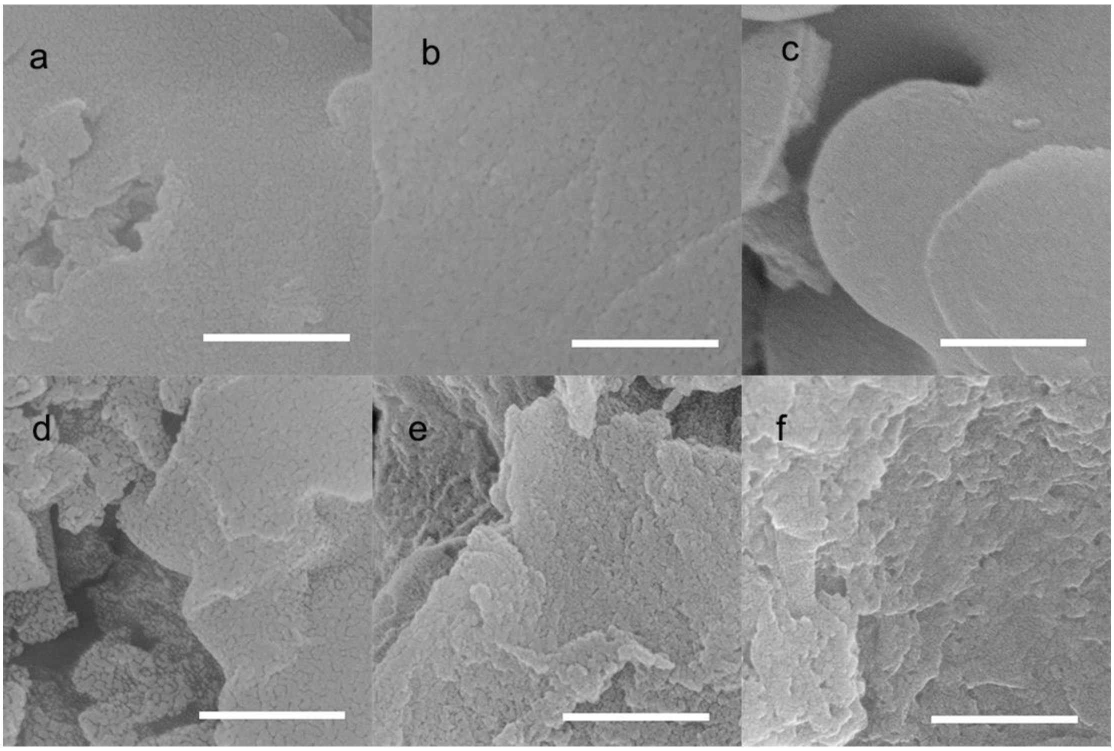

Supplementary Figure 4. SEM images of KAP-Pd-PEPPSI. (a, d) KAP-Pd-PEPPSI-1, (b, e) KAP-Pd-PEPPSI-2 and (c, f) KAP-Pd-PEPPSI-3. (a-c): KAP-Pd-PEPPSI. (d-f): KAP-Pd-PEPPSI after swelling in DMF. Scale bars: 500 nm

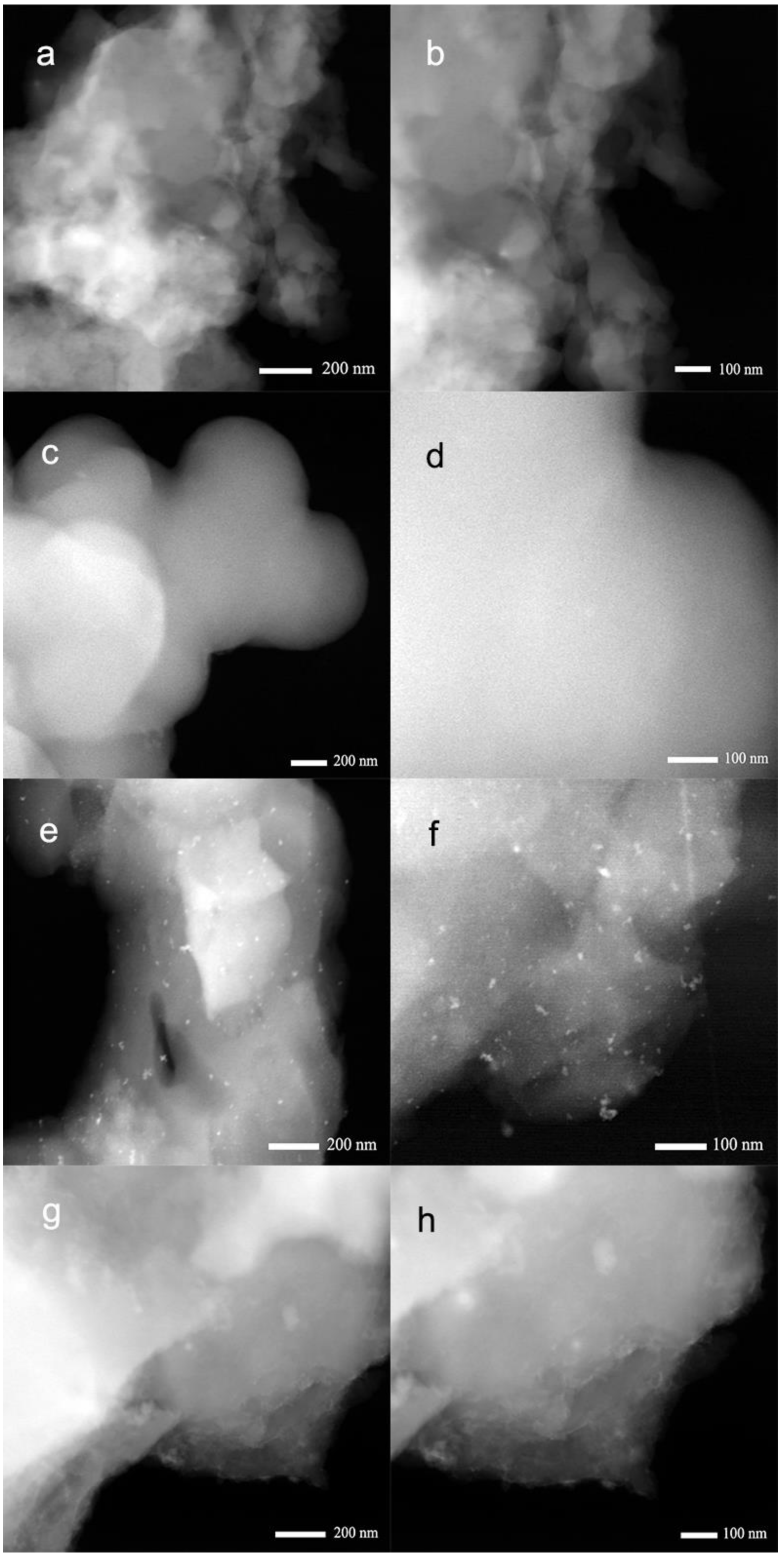

Supplementary Figure 5. STEM images of KAP-Pd-PEPPSI. (a-b) KAP-Pd-PEPPSI-1, (c-d) KAP-Pd-PEPPSI-2, (e-f) KAP-Pd-PEPPSI-2 after being reduced by $NaBH_4$ and (g-h) KAP-Pd-PEPPSI-3.

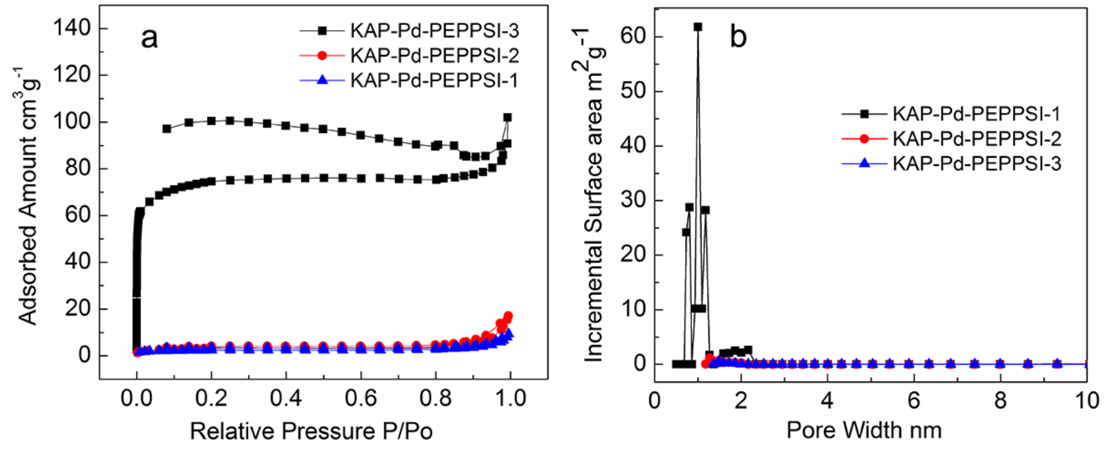

Supplementary Figure 6. Textural properties of catalysts. (a) N_2 sorption isotherms collected at 77 K and (b) pore size distributions of KAP-Pd-PEPPSI. The BET surface area in KAP-Pd-PEPPSI-1 is 227 cm^3 g^{-1}, which decreases to 10 cm^3 g^{-1} and 7 cm^3 g^{-1}, respectively, in KAP-Pd-PEPPSI-2 and KAP-Pd-PEPPSI-3.

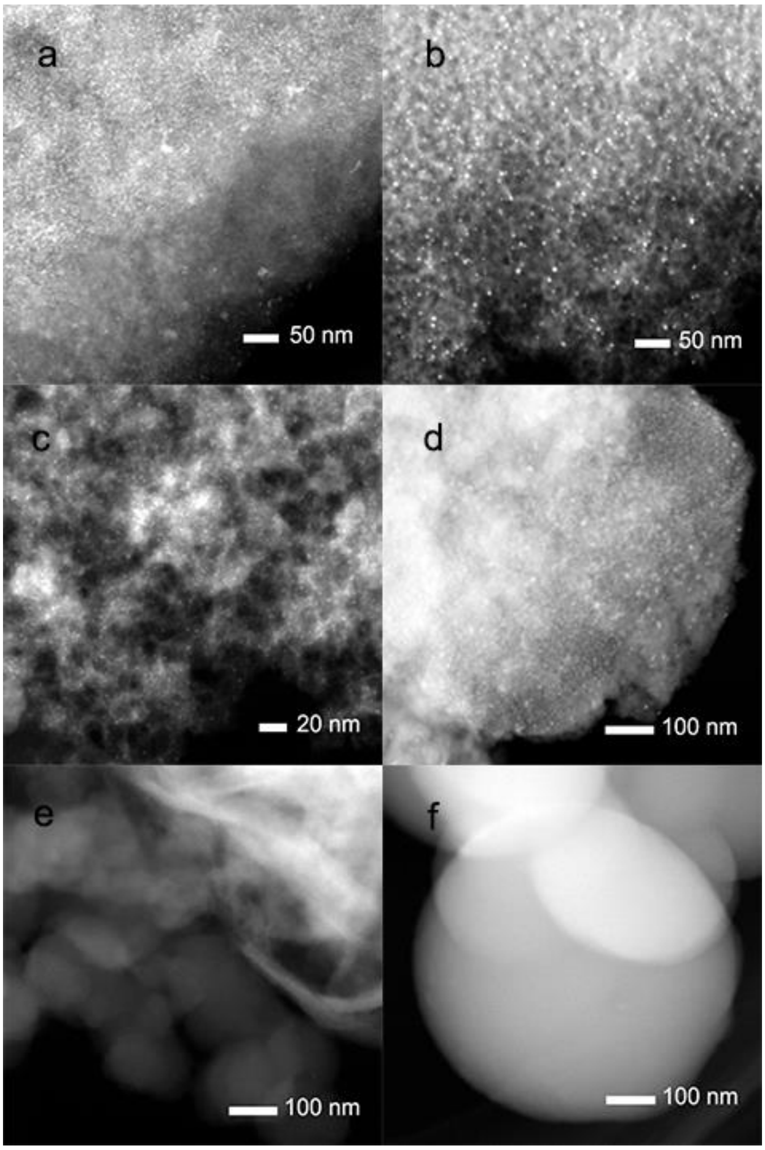

Supplementary Figure 7. STEM images of reference catalysts. (a) Fresh Pd@C, (b) Pd@NC, (c) Pd@PC, (d) Pd@SiO_2, (e) KAP-Pd-PPh_3 and (f) POP-Pd-PEPPSI. The STEM morphologies of fresh Pd@C, Pd@NC, Pd@PC, Pd@SiO_2 (a-d, supported Pd catalysts) show uniform distributed nanoparticles with sizes ranging from several nanometers to tens of nanometers, whereas KAP-Pd-PPh_3 and POP-Pd-PEPPSI (e-f, polymer supported Pd complex precatalysts) show even more uniform distributed Pd species without formation of nanoparticles.

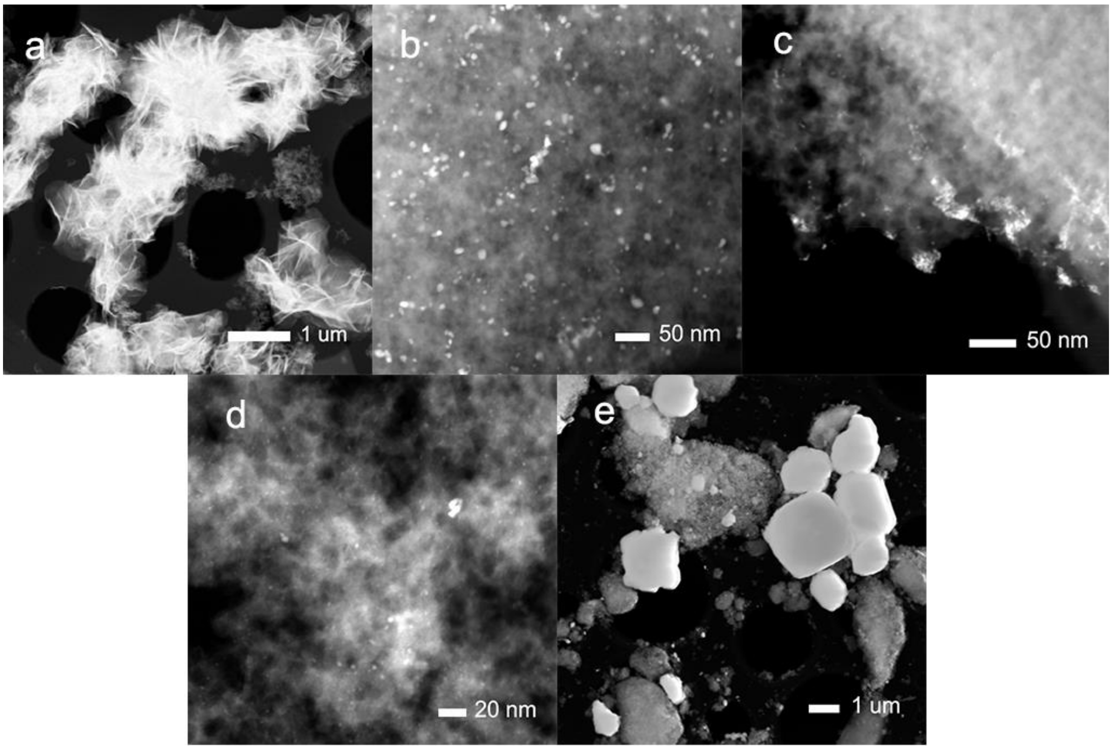

Supplementary Figure 8. STEM images of different catalysts after a single cycle. (a) Pd-PEPPSI, (b) Pd@C, (c) Pd@NC, (d) Pd@PC, (e) Pd@SiO_2.

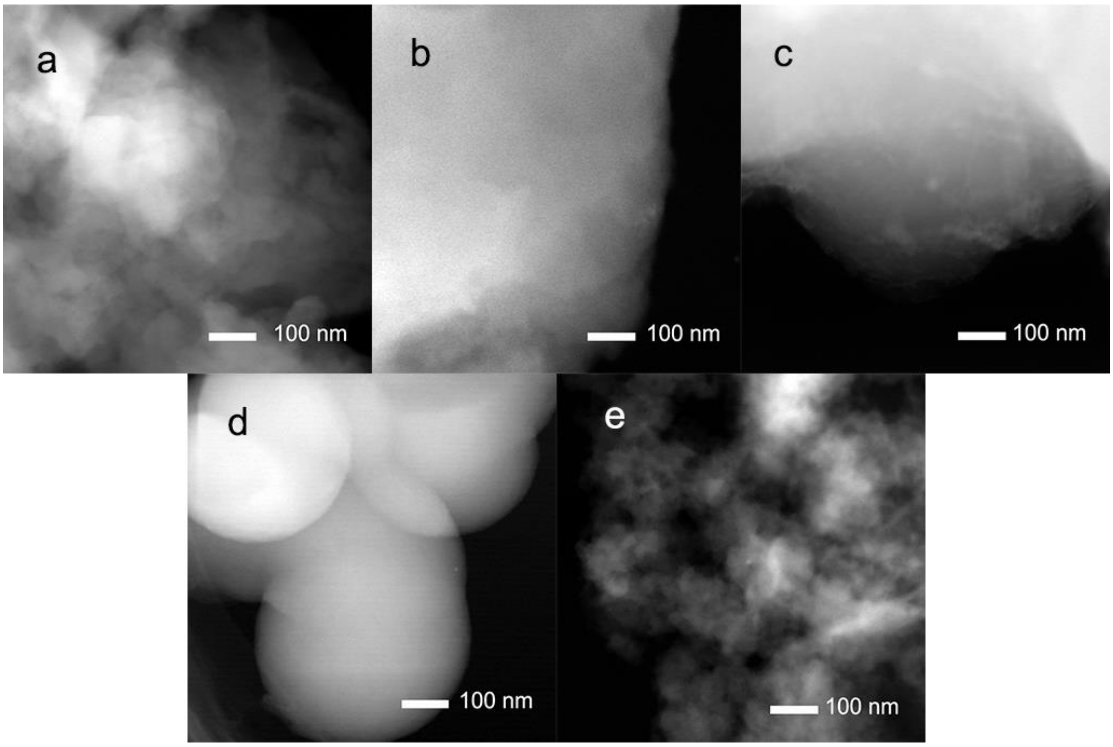

Supplementary Figure 9. STEM images of different catalysts after a single cycle. (a) KAP-Pd-PEPPSI-1, (b) KAP-Pd-PEPPSI-2, (c) KAP-Pd-PEPPSI-3, (d) POP-Pd-PEPPSI, (e) KAP-Pd-PPh_3.

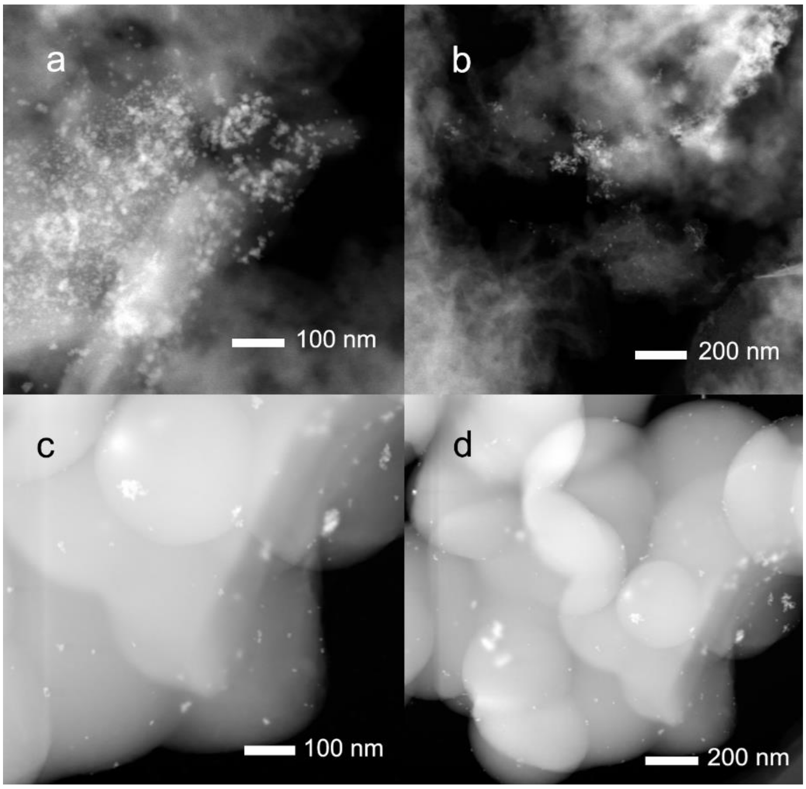

Supplementary Figure 10. STEM images of different catalysts after reusability test. STEM images of (a-b) KAP-Pd-PPh_3 after 5 cycles and (c-d) POP-Pd-PEPPSI after 5 cycles.

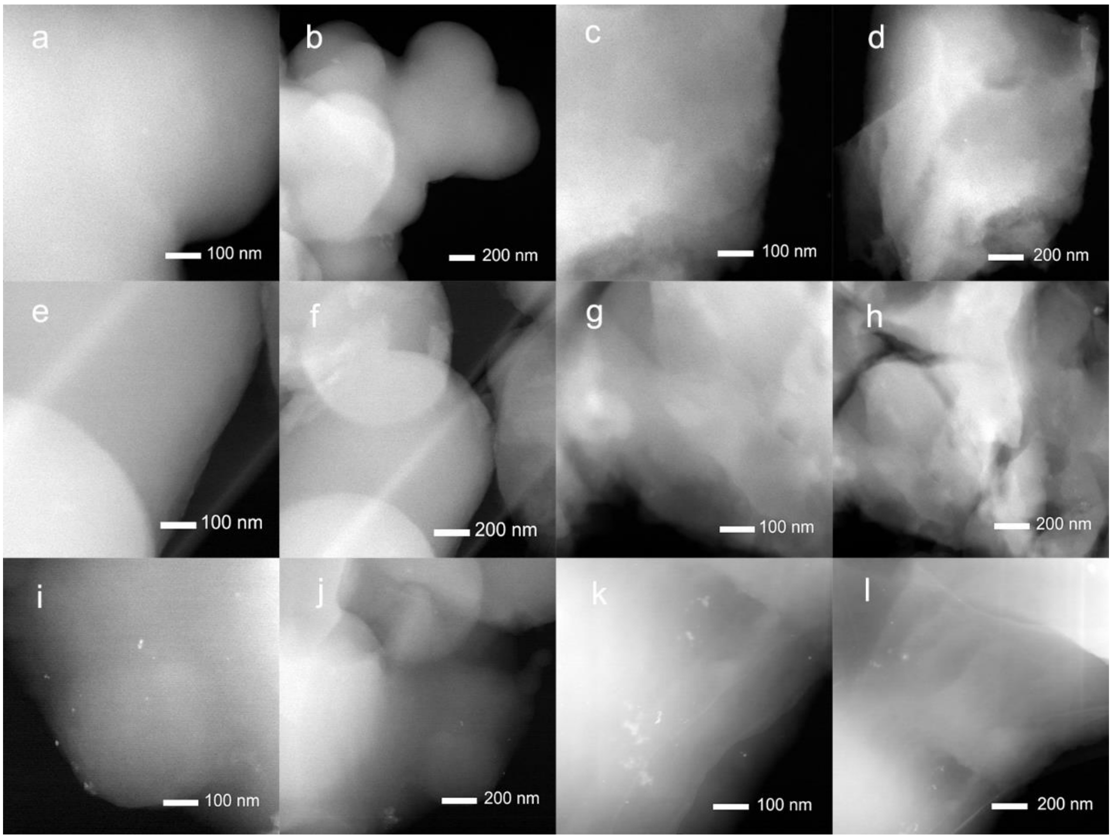

Supplementary Figure 11. STEM images of KAP-Pd-PEPPSI-2. (a-b) fresh catalyst, (c-d) after 1 cycles, (e-f) after 3 cycles, (g-h) after 5 cycles, (i-j) after 7 cycles, (k-l) after 9 cycles.

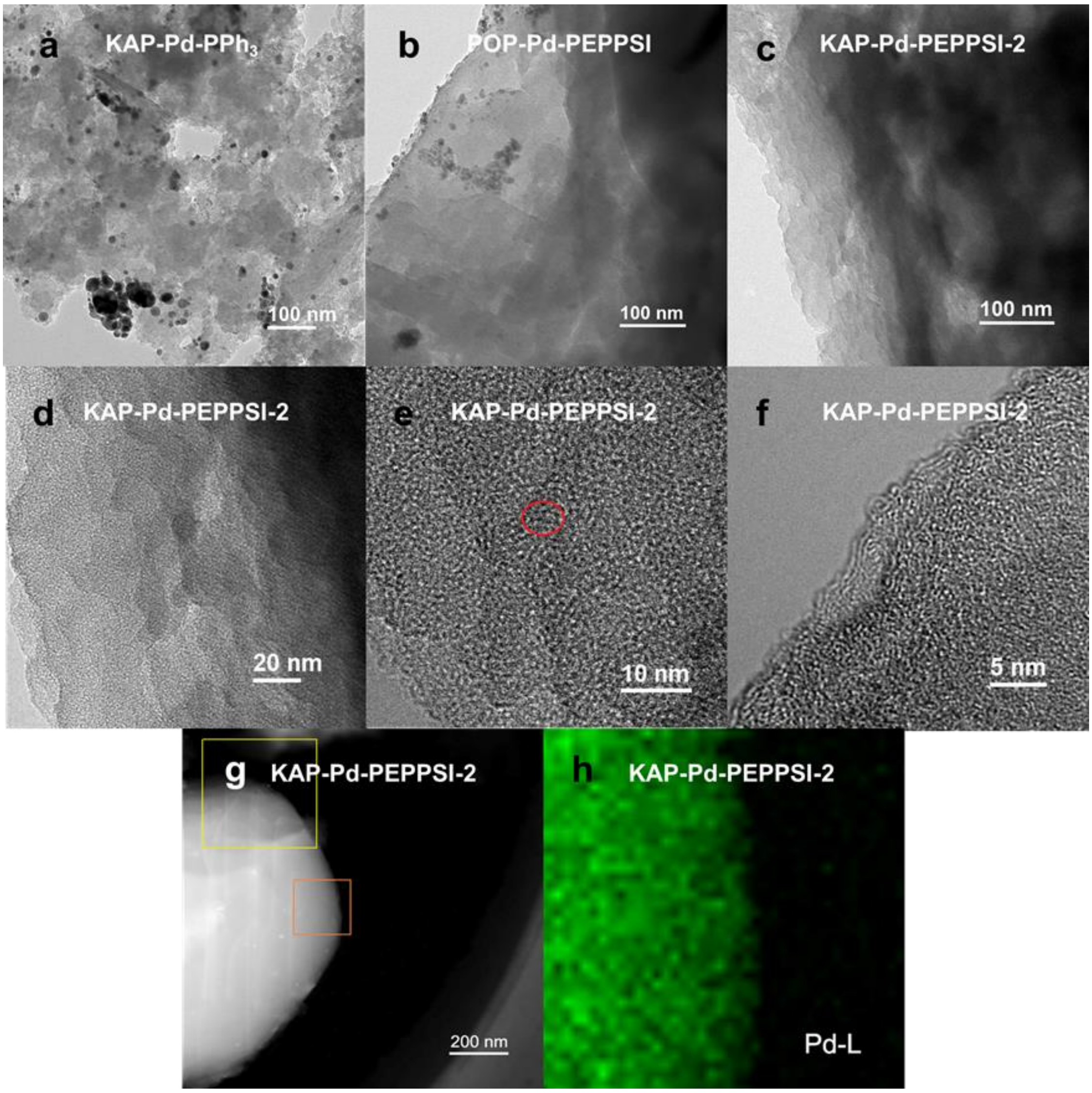

Supplementary Figure 12. TEM images of different catalysts after being used for five times. (a) KAP-Pd-PPh_3, (b) POP-Pd-PEPPSI, (c-f) KAP-Pd-PEPPSI-2, (g) STEM image of KAP-Pd-PEPPSI-2, (h) EDS mapping images for Pd in KAP-Pd-PEPPSI-2.

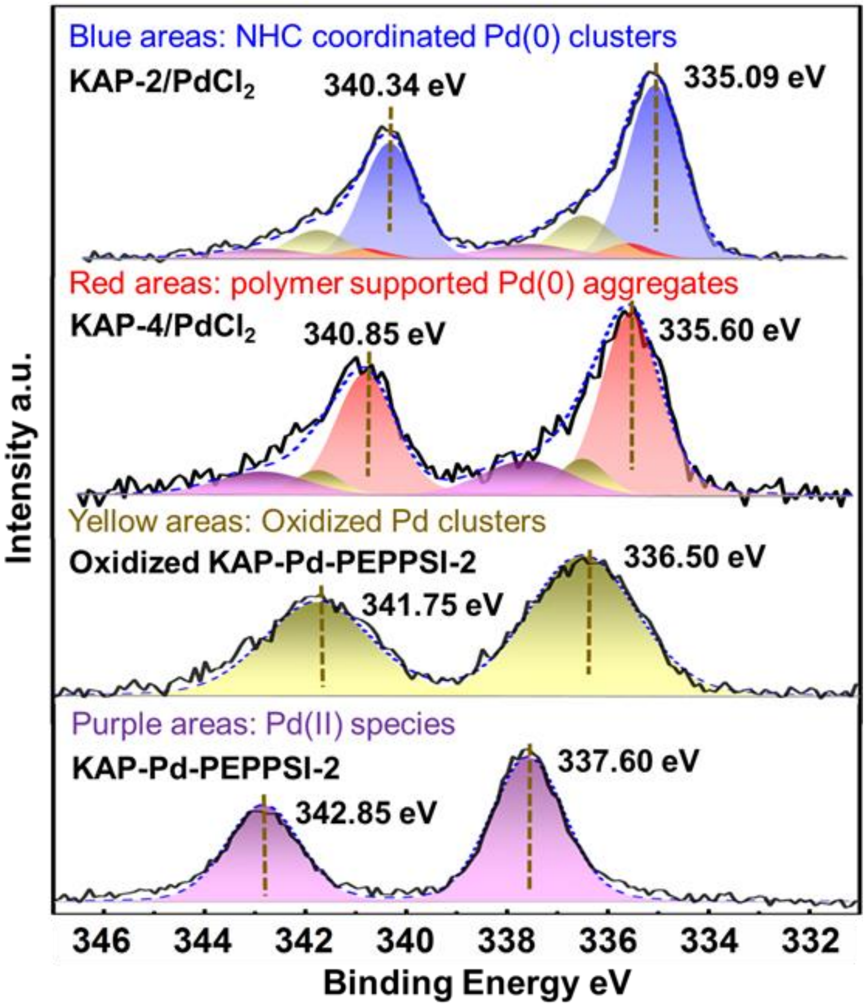

Supplementary Figure 13. Determination of the Pd components. Control experiments to investigate the Pd components involving in the supported Pd catalyzed fluorocarbonylation of indole. Blue areas: NHC coordinated Pd(0) clusters (Prepared by coordination of NHC-functionalized KAP with *in-situ* formed Pd(0) species form $PdCl_2$). Red areas: polymer supported Pd(0) aggregates (Prepared by deposition of *in-situ* formed Pd(0) aggregates form $PdCl_2$ onto non-NHC-functionalized KAP). Yellow areas: oxidized Pd clusters (Prepared by oxidation of *in-situ* formed Pd(0) clusters in KAP-Pd-PEPPSI-2 using the reaction mixture of fluorocarbonylation reaction in the absence of CsF). Purple areas: Pd(II) complexes (Pd(II) complexes in the original KAP-Pd-PEPPSI).

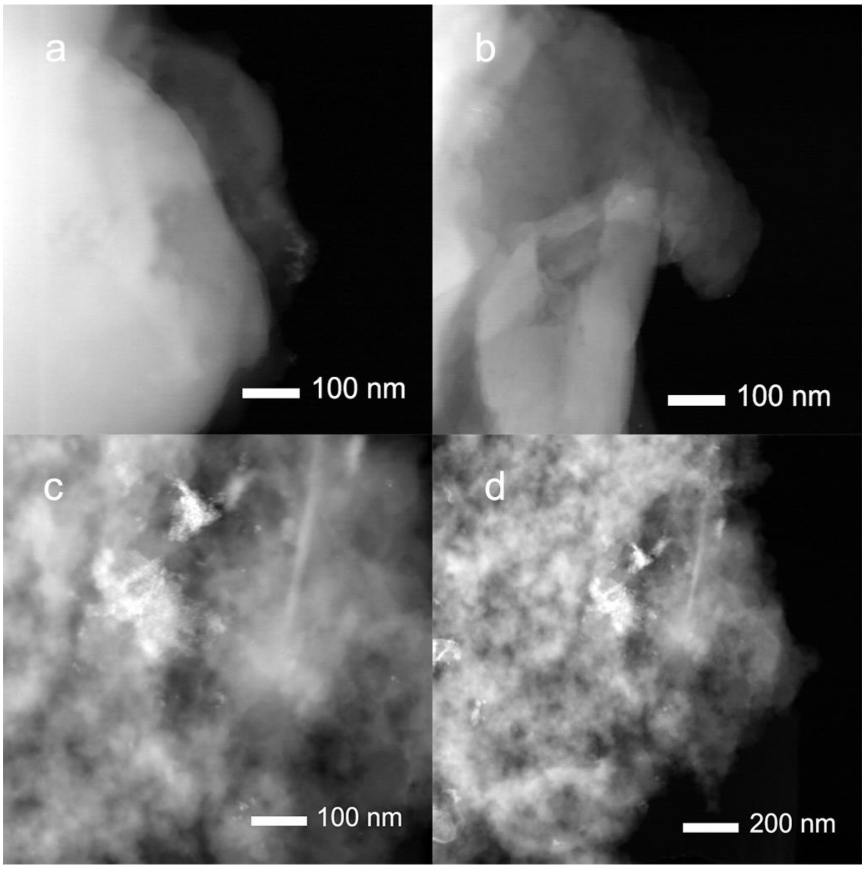

Supplementary Figure 14. STEM images of the spent catalysts in control experiment. (a-b) KAP-2/$PdCl_2$ and (c-d) KAP-4/$PdCl_2$.

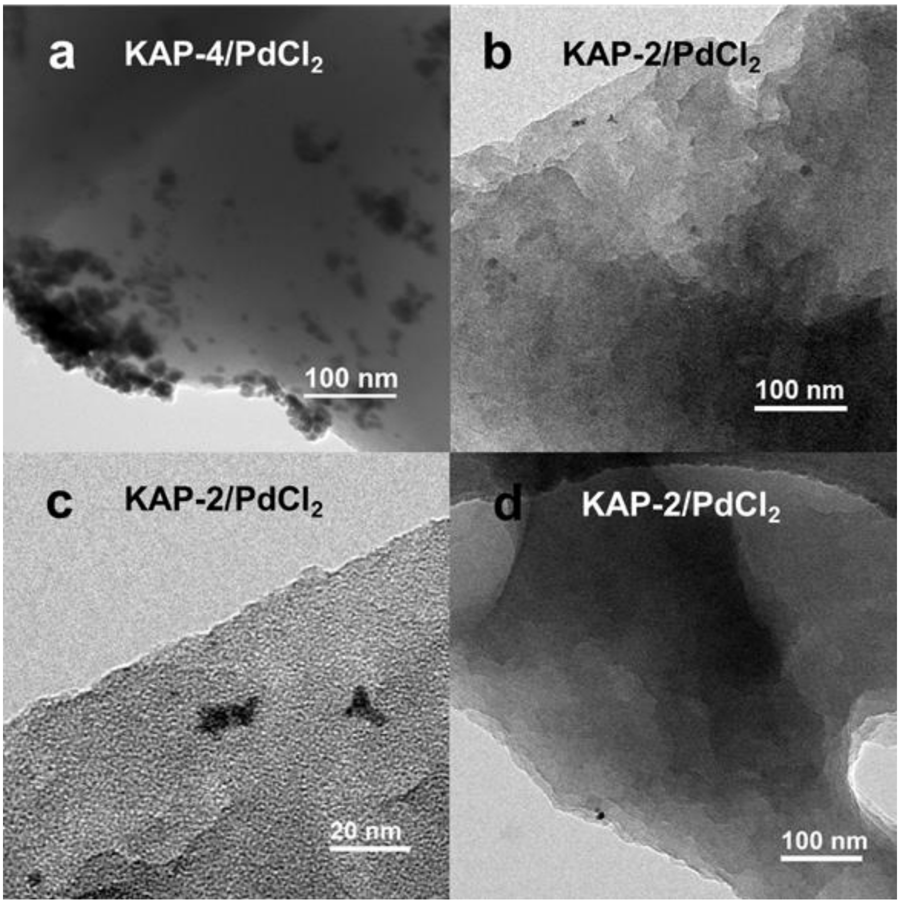

Supplementary Figure 15. TEM images of the spent catalysts in control experiment. (a) KAP-4/$PdCl_2$ and (b-d) KAP-2/$PdCl_2$.

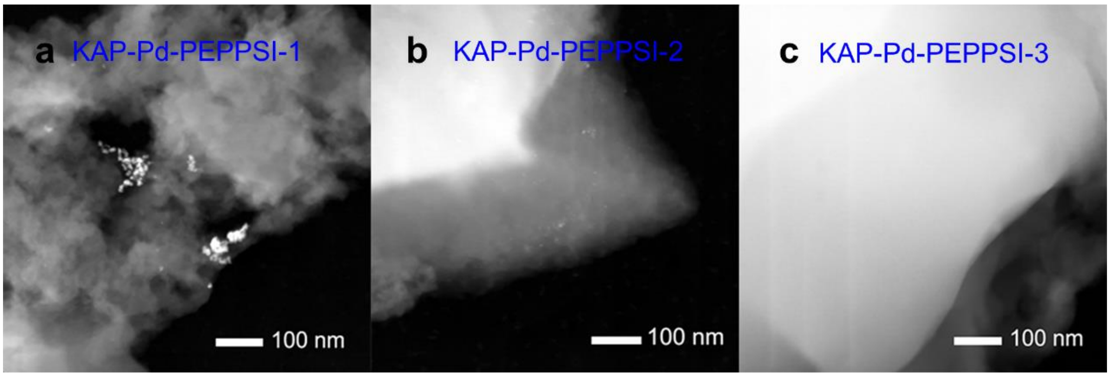

Supplementary Figure 16. STEM images of different KAP-Pd-PEPPSI after the 6th cycle. (a) KAP-Pd-PEPPSI-1, (b) KAP-Pd-PEPPSI-2 and (c) KAP-Pd-PEPPSI-3.

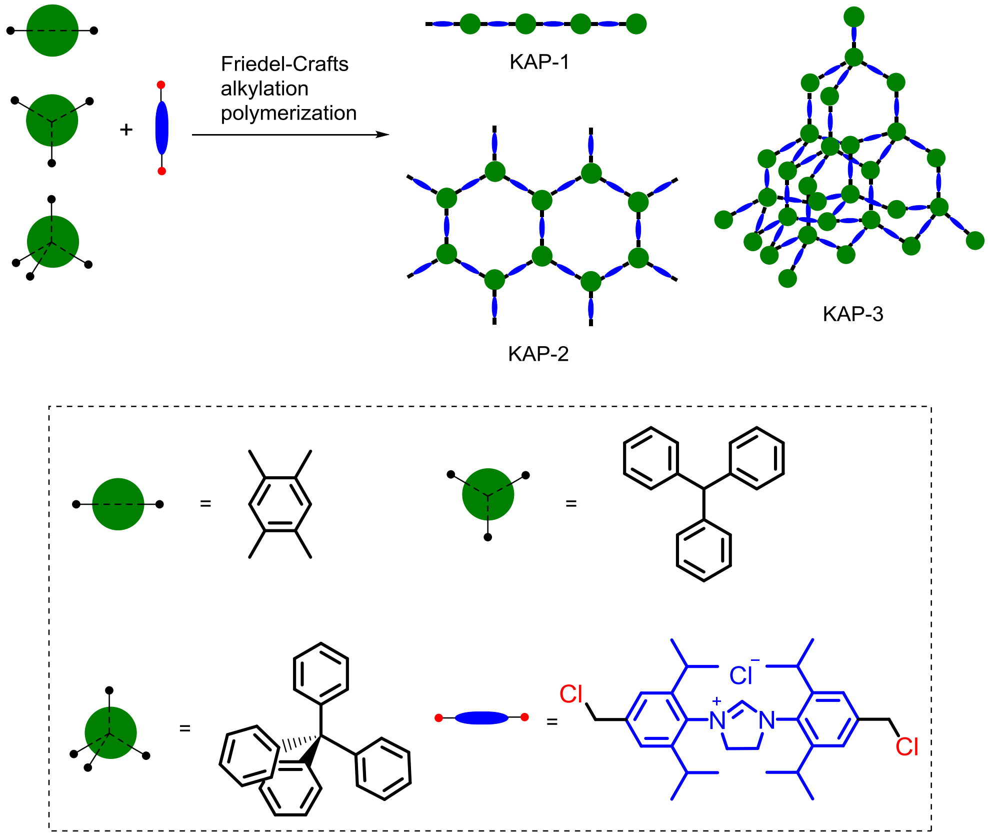

Supplementary Figure 17. Ideal structure models of different KAP-Pd-PEPPSI. The NHC precursor-functionalized KAPs with different crosslinking structures were prepared by Friedel-Crafts alkylation of chloromethyl functionalized NHC precursor with tetramethylbenzene, triphenylmethane and tetraphenylmethane.

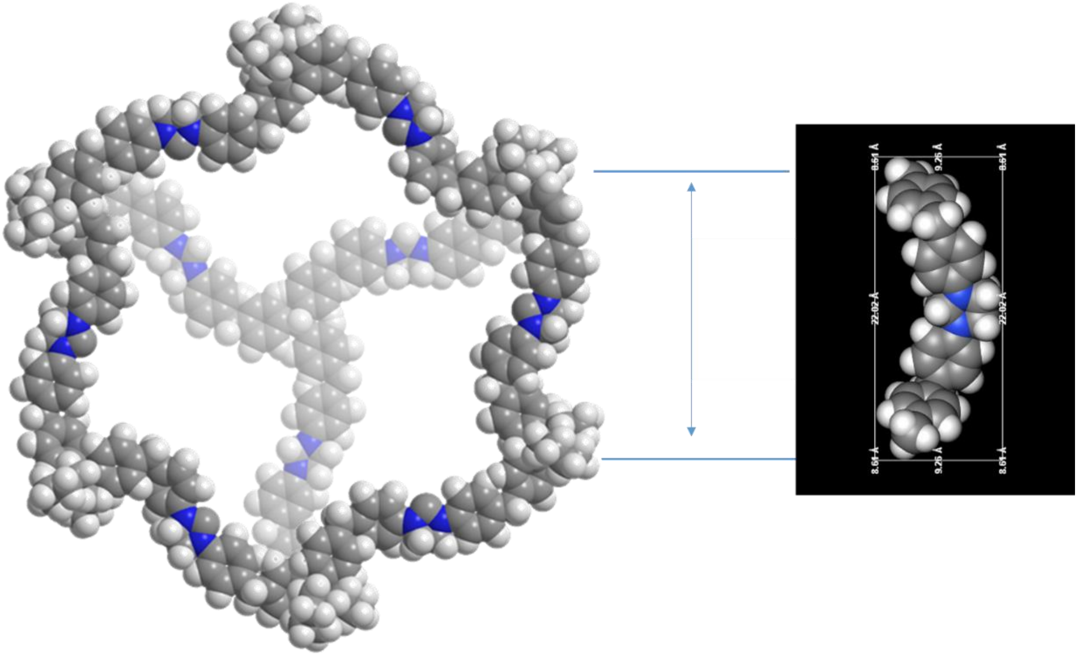

Supplementary Figure 18. Ideal molecular structure model of KAP-Pd-PEPPSI-3. Molecular cage and size of the repeat molecular unit.

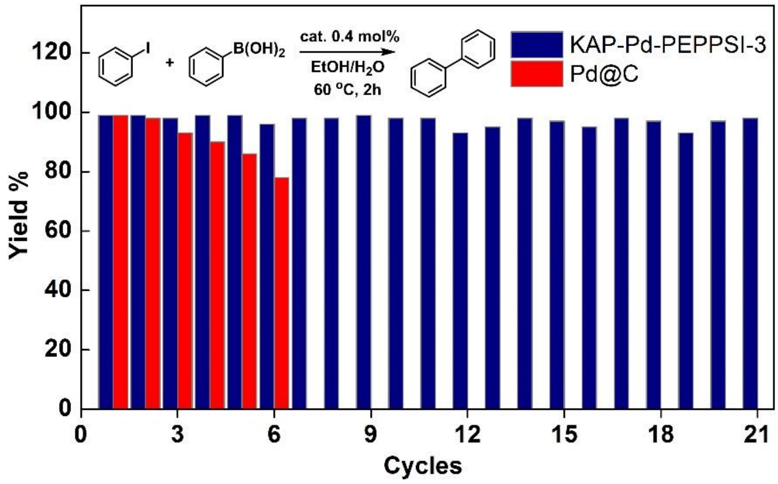

Supplementary Figure 19. Recyclability of KAP-Pd-PEPPSI-3 in Suzuki coupling reaction. Reaction conditions: catalyst 0.4 mol%, iodobenzene 1.0 mmol, phenylboronic acid 1.5 mmol, K_3PO_4 3.0 mmol, EtOH/H_2O 4 mL, 60 °C, 2 h.

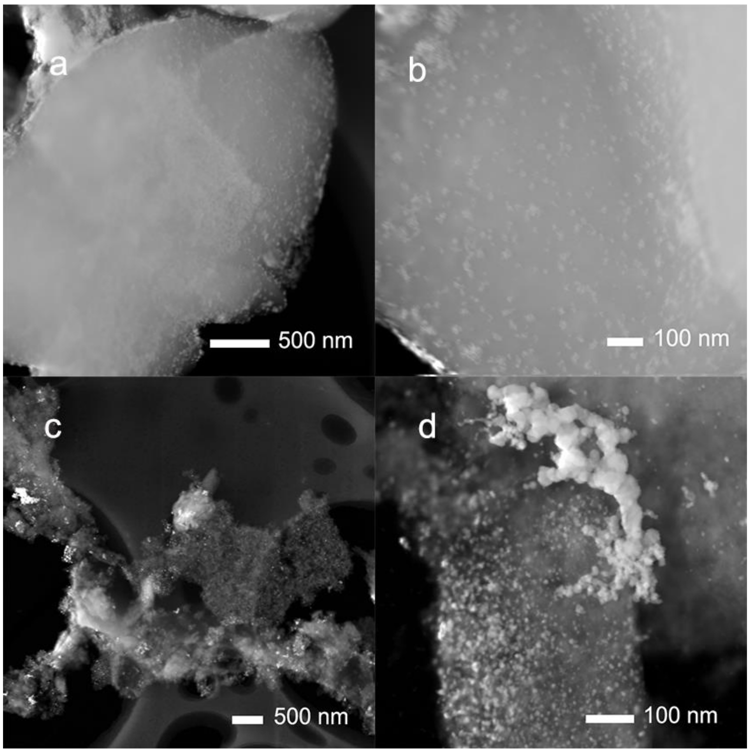

Supplementary Figure 20. STEM images of the spent catalysts. (a-b) Pd@C after 6 cycles and (c-d) KAP-Pd-PEPPSI-3 after 21 cycles in Suzuki coupling reaction.

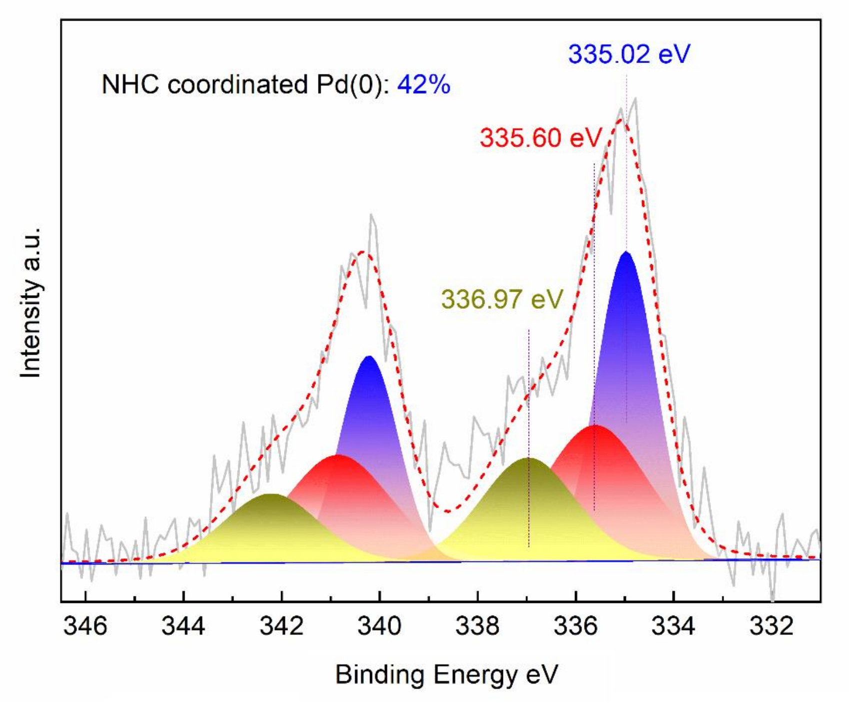

Supplementary Figure 21. XPS spectra of recycled KAP-Pd-PEPPSI-3. Pd 3d XPS spectra of recycled KAP-Pd-PEPPSI-3 after the 21th cycle in Suzuki coupling reaction.

Supplementary Tables

Supplementary Table 1. Pd content in different catalysts and the corresponding ligand-to-metal ratio *a*

Cat.	Pd loading wt%	L/Pd
Pd@C	5.6	-
Pd@NC[1]	2.1	56
Pd@PC[2,3]	1.2	19
Pd@SiO_2[4]	2.1	-
KAP-Pd-PPh_3[5]	2.2	5.34
POP-Pd-PEPPSI[6]	2.5	4.22
KAP- Pd-PEPPSI-1[5,7,8]	2.5	6.32
KAP- Pd-PEPPSI-2[5,7,8]	2.4	5.79
KAP- Pd-PEPPSI-3[5,7,8]	2.9	5.50

a These catalysts were synthesized according to the published methods[1-8]. The Pd content in catalysts were determined by ICP-MS using In as a reference and ligand-to-metal ratios were calculated from the XPS results.

Supplementary Table 2. Optimization of fluorocarbonylation of indoles [a]

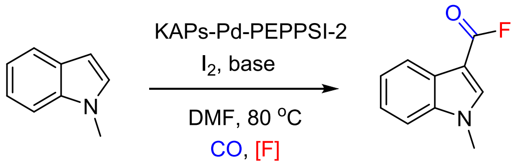

Entry	Solvent	[F-]	Base	Yield (%)
1	DMF	CsF	Cs_2CO_3	24
2	DMF	CsF	K_2CO_3	12
3	DMF	CsF	K_3PO_4	8
4	DMF	CsF	DBU	trace
**5**[b]	**DMF**	**CsF**	**DABCO**	**89(79)**
6	DMF	KF	DABCO	74
7	DMF	NH_4F	DABCO	trace
8	DMF	NBu_4F	DABCO	32
9	Toluene	CsF	DABCO	trace
10	Dioxane	CsF	DABCO	trace
11[c]	DMF	CsF	DABCO	trace
12[d]	DMF	CsF	DABCO	88
13[e]	DMF	CsF	DABCO	83
14[f]	DMF	CsF	DABCO	54
15[g]	DMF	CsF	DABCO	86

[a] Conditions: **1a** (0.25 mmol), CsF (1 mmol), catalyst (5 mol%), I_2 (0.5 mmol), base (0.5 mmol), DMF (2 mL), 80 °C, 24 h. [b] Isolated yield in bracket. [c] Mecury test. [d] 3 mol% catalyst. [e] 2 mol% catalyst. [f] 1 mol% catalyst. [g] 48 h.

Supplementary Table 3. Pd leaching conditions of different catalyst in washing test [a]

Entry	Catalyst	Pd leaching (%)
1	Pd@C	39.6
2	Pd@NC	19.6
3	Pd@PC	12.4
4	Pd@SiO_2	27.4
5	KAP-Pd-PPh_3	60.4
6	POP-Pd-PEPPSI	41.3
7	KAP-Pd-PEPPSI-1	39.6
8	KAP-Pd-PEPPSI-2	16.1
9	KAP-Pd-PEPPSI-3	7.0
10[b]	KAP-Pd-PEPPSI-3	5.5
11[c]	KAP-Pd-PEPPSI-3	91.0

[a] The washing test was performed to determine the extent of Pd leaching from the catalysts upon treatment with the reactants for 4 h at 80 ^{o}C in the absence of CsF. [b]After reaction for 4 h in the presence of CsF (3% yield). [c]Catalyst 0.6 mol% (21% yield).

Supplementary Table 4. The time-course analyses of Pd distribution as well as the corresponding reaction activity in flurocarbonylation of indole

Time	Pd@C		KAP-Pd-PEPPSI	
	Yield %	Pd in solution wt%	Yield %	Pd in solution wt%
2	45	37.3	0	2
4	67	40.7	3	5.5
6	74	35.1	55	1.9
8	81	28.8	69	0.6
10	84	16.4	78	0.5
24	89	2	86	0.2

Supplementary Table 5. Aminocarbonylation of indole with weakly nucleophilic aromatic amines using various catalyst systems [a]

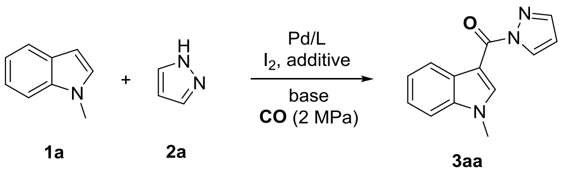

Entry	Reaction Conditions	Yield %
1	(Arndtsen's Conditions)1a 0.37 mmol, 2a 0.25 mmol, $Pd(PtBu_3)_2$ 5 mol% $EtN(iPr)_2$ 0.74 mmol, I_2 0.37 mmol, Bu_4NCl 0.25 mmol, CH_3CN 0.4 mL, 80 °C, 24 h	14
2	(Manabe's Conditions)1a 0.25 mmol, 2a 0.37 mmol, $Pd(OAc)_2$ 5 mol% Xantphos 10 mol%, Et_3N 1 mmol, I_2 0.47 mmol, KF 0.625 mmol, DMF 3 mL, 80 °C, 24 h	11
3	(This Work)1a 0.25 mmol, 2a 1.25 mmol, KAPs-Pd-PEPPSI 5 mol%, DABCO 0.5 mmol, I_2 0.5 mmol, CsF 1 mmol, DMF 2 mL, 80 °C, 24 h	74

[a] Isolated yield.

Supplementary Table 6. Control reactions to investigate the role of fluoride [a]

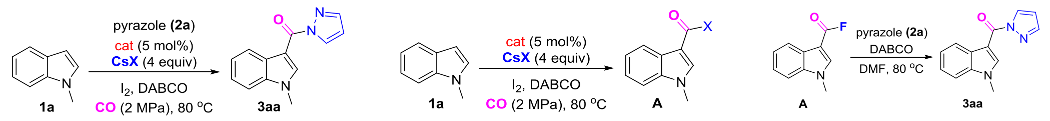

Halides	Yield %	Halides	Yield %	Yield %
-	n.d.	-	n.d.	84
CsF	78	CsF	89	
CsCl	n.d.	CsCl	n.d.	
CsBr	n.d.	CsBr	n.d.	
CsI	n.d.	CsI	n.d.	

[a] Conditions: **1a** (0.25 mmol), CsX (1 mmol), KAP-Pd-PEPPSI-2 (5 mol%), I_2 (0.5 mmol), base (0.5 mmol), DMF (2 mL), 80 °C, 24 h. HPLC yield.

Supplementary Table 7. KAP-Pd-PEPPSI catalyzed Suzuki coupling reactions of Aryl halides with phenylboronic acid[a].

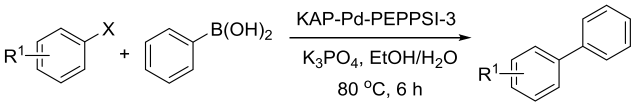

Entry	Aryl halide	Product	Yield[b] (%)
1[c]	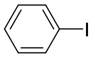	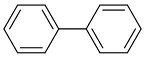	99%
2	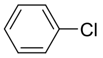	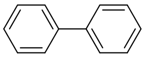	98%
3	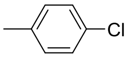	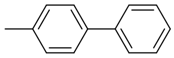	97%
4	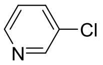	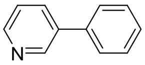	94%
5	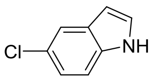	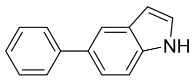	98%
6	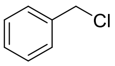	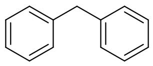	92%

[a]Reaction conditions: catalyst 0.4 mol%, aryl halide 1.0 mmol, aryl boronic acid 1.5 mmol, K_3PO_4 3.0 mmol, EtOH/H_2O 4 mL, 80 °C, 6 h. [b]GC yield. [c]60 °C, 2 h, the Pd content in the filtrate after the completion of reaction was determined to be 0.06 ppm by ICP-MS.

Supplementary Note 1

Detailed Descriptions for Products

1-methyl-1H-indole-3-carbonyl fluoride

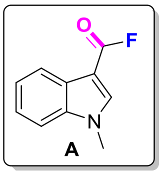

Purified by column chromatography (10:1 PE/EA); white solid; 79% yield; *Rf* = 0.28 (5:1 PE/EA). ^{1}H NMR (400 MHz, $CDCl_3$) δ 7.99 – 7.97 (m, 1H), 7.70 (s, 1H), 7.29 – 7.23 (m, 3H), 3.75 (s, 3H). ^{13}C NMR (100 MHz, $CDCl_3$) δ 156.3, 153.0, 137.8, 137.5, 136.5, 126.8, 123.9, 123.1, 121.1, 110.4, 109.9, 33.8, 33.6. The spectral data obtained agree with those reported in the literature.[9]

(1-methyl-1H-indol-3-yl)(1H-pyrazol-1-yl)methanone

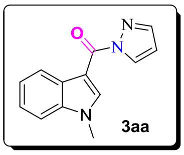

Purified by column chromatography (10:1 PE/EA); light red solid; 74% yield; *Rf* = 0.36 (5:1 PE/EA). ^{1}H NMR (400 MHz, $CDCl_3$) δ 8.82 (s, 1H), 8.50 (s, 2H), 7.75 (s, 1H), 7.35 (s, 3H), 6.45 (s, 1H), 3.86 (d, *J* = 2.3 Hz, 3H). ^{13}C NMR (100 MHz, $CDCl_3$) δ 160.9, 143.1, 140.1, 136.8, 129.7, 128.9, 123.4, 122.8, 122.4, 109.8, 108.0, 106.1, 33.7. HRMS (ESI) m/z: calculated for $C_{13}H_{11}N_3ONa$ $[M + Na]^+$ 248.0800, found 248.0797.

(1,4-dimethyl-1H-indol-3-yl)(1H-pyrazol-1-yl)methanone

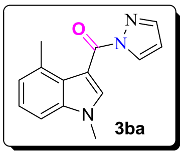

Purified by column chromatography (10:1 then 5:1 PE/EA); light red solid; 83% yield; *Rf* = 0.29 (5:1 PE/EA). ^{1}H NMR (400 MHz, $CDCl_3$) δ 8.45 (d, *J* = 2.6 Hz, 1H), 8.12 (s, 1H), 7.75 (s, 1H), 7.18 (m, 3H), 6.48 (dd, *J* = 2.6, 1.5 Hz, 1H), 3.78 (s, 3H), 2.75 (s, 3H). ^{13}C NMR (100 MHz, $CDCl_3$) δ 161.2, 143.2, 139.7, 137.6, 132.7,

129.9, 126.6, 124.6, 123.6, 108.5, 107.6, 107.2, 33.7, 22.0. HRMS (ESI) m/z: calculated for $C_{14}H_{13}N_3ONa$ $[M + Na]^+$ 262.0956, found 262.0954.

(1,5-dimethyl-1H-indol-3-yl)(1H-pyrazol-1-yl)methanone

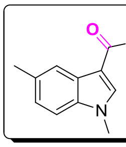

3ca

Purified by column chromatography (10:1 PE/EA); light red solid; 81% yield; *Rf* = 0.38 (5:1 PE/EA). ^1H NMR (400 MHz, DMSO) δ 8.89 (s, 1H), 8.55 (dd, *J* = 2.7, 0.5 Hz, 1H), 8.12 (s, 1H), 7.94 (d, *J* = 0.8 Hz, 1H), 7.50 (d, *J* = 8.3 Hz, 1H), 7.18 (dd, *J* = 8.4, 1.3 Hz, 1H), 6.64 (dd, *J* = 2.7, 1.5 Hz, 1H), 3.93 (s, 3H), 2.47 (s, 3H). ^{13}C NMR (100 MHz, DMSO) δ 160.1, 143.5, 140.5, 135.1, 131.5, 129.6, 128.3, 124.6, 121.0, 110.7, 108.5, 103.9, 33.4, 21.3. HRMS (ESI) m/z: calculated for $C_{14}H_{13}N_3ONa$ $[M + Na]^+$ 262.0956, found 262.0957.

(1,6-dimethyl-1H-indol-3-yl)(1H-pyrazol-1-yl)methanone

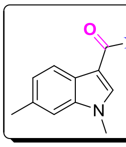

3da

Purified by column chromatography (10:1 PE/EA); light red solid; 91% yield; *Rf* = 0.40 (5:1 PE/EA). ^1H NMR (400 MHz, DMSO) δ 8.85 (s, 1H), 8.56 (d, *J* = 2.7 Hz, 1H), 8.18 (d, *J* = 8.1 Hz, 1H), 7.94 (d, *J* = 0.7 Hz, 1H), 7.40 (s, 1H), 7.15 (d, *J* = 8.1 Hz, 1H), 6.63 (dd, *J* = 2.7, 1.5 Hz, 1H), 3.91 (s, 3H), 2.46 (s, 3H). ^{13}C NMR (100 MHz, DMSO) δ 160.1, 143.6, 140.2, 137.0, 132.6, 129.6, 125.9, 124.1, 120.9, 110.8, 108.5, 104.4, 33.3, 21.3. HRMS (ESI) m/z: calculated for $C_{14}H_{13}N_3ONa$ $[M + Na]^+$ 262.0956, found 262.0952.

(1,7-dimethyl-1H-indol-3-yl)(1H-pyrazol-1-yl)methanone

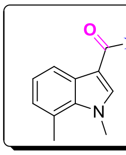

3ea

Purified by column chromatography (10:1 then 5:1 PE/EA); light red solid; 69% yield; *Rf* = 0.31 (5:1 PE/EA). ^1H NMR (400 MHz, DMSO) δ 8.82 (s, 1H), 8.59–8.52 (m, 1H), 8.19 (d, *J* = 7.9 Hz, 1H), 7.98–7.88 (m, 1H), 7.16 (t, *J* = 7.6 Hz,

1H), 7.03 (d, *J* = 7.1 Hz, 1H), 6.63 (dd, *J* = 2.7, 1.5 Hz, 1H), 4.19 (s, 3H), 2.77 (s, 3H). ^{13}C NMR (100 MHz, DMSO) δ 160.0, 143.5, 142.0, 135.2, 129.7, 129.2, 125.8, 122.6, 119.2, 108.5, 103.8, 37.5, 19.0. HRMS (ESI) m/z: calculated for $C_{14}H_{13}N_3ONa$ [M + Na]$^+$ 262.0956, found 262.0954.

(1,2-dimethyl-1H-indol-3-yl)(1H-pyrazol-1-yl)methanone

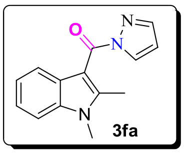

Purified by column chromatography (10:1 then 5:1 PE/EA); light red solid; 57% yield; *Rf* = 0.13 (5:1 PE/EA). ^{1}H NMR (400 MHz, DMSO) δ 8.45 (d, *J* = 2.7 Hz, 1H), 7.86 (s, 1H), 7.56 (d, *J* = 8.2 Hz, 1H), 7.37 (d, *J* = 7.9 Hz, 1H), 7.22 (t, *J* = 7.6 Hz, 1H), 7.12 (t, *J* = 7.5 Hz, 1H), 6.64 (s, 1H), 3.78 (s, 3H), 2.47 (s, 3H). ^{13}C NMR (100 MHz, DMSO) δ 163.3, 146.2, 143.1, 136.3, 130.2, 126.3, 122.0, 121.3, 120.0, 110.1, 109.0, 105.3, 29.9, 12.4. HRMS (ESI) m/z: calculated for $C_{14}H_{13}N_3ONa$ [M + Na]$^+$ 262.0956, found 262.0957.

(6-chloro-1-methyl-1H-indol-3-yl)(1H-pyrazol-1-yl)methanone

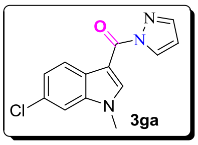

Purified by column chromatography (10:1 PE/EA); light red solid; 69% yield; *Rf* = 0.35 (5:1 PE/EA). ^{1}H NMR (400 MHz, DMSO) δ 8.94 (s, 1H), 8.56 (d, *J* = 2.8 Hz, 1H), 8.27 (d, *J* = 8.5 Hz, 1H), 7.96 (s, 1H), 7.77 (d, *J* = 1.7 Hz, 1H), 7.33 (dd, *J* = 8.5, 1.8 Hz, 1H), 6.65 (dd, *J* = 2.7, 1.5 Hz, 1H), 3.95 (s, 3H). ^{13}C NMR (100 MHz, DMSO) δ 160.0, 143.9, 141.4, 137.2, 129.7, 127.9, 126.7, 122.7, 122.5, 111.2, 108.8, 104.7, 33.6. HRMS (ESI) m/z: calculated for $C_{13}H_{10}ClN_3ONa$ [M + Na]$^+$ 282.0410, found 282.0408.

(6-bromo-1-methyl-1H-indol-3-yl)(1H-pyrazol-1-yl)methanone

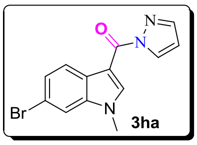

Purified by column chromatography (10:1 PE/EA); light red solid; 64% yield; *Rf* = 0.34 (5:1 PE/EA). ^{1}H NMR (400 MHz, DMSO) δ 8.96 (s, 1H), 8.60 (dd, *J* = 2.8, 0.6 Hz, 1H), 8.25 (d, *J* = 8.5 Hz, 1H), 8.02–7.97 (m, 1H), 7.94 (d, *J* = 1.6 Hz, 1H), 7.49 (dd, *J* = 8.5, 1.8 Hz, 1H), 6.68 (dd, *J* = 2.8, 1.5 Hz, 1H), 3.98 (s, 3H). ^{13}C NMR (100 MHz, DMSO) δ 160.0, 143.9, 141.3, 137.6, 129.7, 127.0, 125.4, 122.8, 115.9, 114.1, 108.8, 104.7, 33.6. HRMS (ESI) m/z: calculated for $C_{13}H_{10}BrN_3ONa$ [M + Na]$^+$ 325.9904, found 325.9912.

(5-methoxy-1-methyl-1H-indol-3-yl)(1H-pyrazol-1-yl)methanone

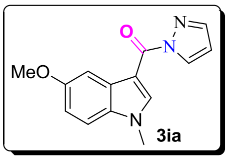

Purified by column chromatography (10:1 then 5:1 PE/EA); white solid; 73% yield; *Rf* = 0.30 (5:1 PE/EA). ^{1}H NMR (400 MHz, DMSO) δ 8.89 (s, 1H), 8.55 (d, *J* = 2.7 Hz, 1H), 7.95 (d, *J* = 0.6 Hz, 1H), 7.83 (d, *J* = 2.5 Hz, 1H), 7.53 (d, *J* = 8.9 Hz, 1H), 6.98 (dd, *J* = 8.9, 2.5 Hz, 1H), 6.64 (dd, *J* = 2.4, 1.7 Hz, 1H), 3.93 (s, 3H), 3.84 (s, 3H). ^{13}C NMR (100 MHz, DMSO) δ 160.1, 156.0, 143.6, 140.5, 131.6, 129.6, 129.1, 112.7, 111.9, 108.5, 104.0, 103.2, 55.3, 33.6. HRMS (ESI) m/z: calculated for $C_{14}H_{13}N_3O_2Na$ [M + Na]$^+$ 278.0905, found 278.0902.

(1-methyl-1H-pyrrolo[2,3-b]pyridin-3-yl)(1H-pyrazol-1-yl)methanone

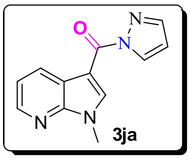

Purified by column chromatography (10:1 then 5:1 PE/EA); light red solid; 70% yield; *Rf* = 0.16 (5:1 PE/EA). ^{1}H NMR (400 MHz, $CDCl_3$) δ 8.98 (s, 1H), 8.72 (dd, *J* = 7.9, 1.6 Hz, 1H), 8.49 (dd, *J* = 2.8, 0.5 Hz, 1H), 8.44 (dd, *J* = 4.7, 1.6 Hz, 1H), 7.78 (d, *J* = 0.7 Hz, 1H), 7.33–7.23 (m, 1H), 6.48 (dd, *J* = 2.8, 1.5 Hz, 1H), 4.00 (s, 3H). ^{13}C NMR (100 MHz, $CDCl_3$) δ 160.5, 147.9, 144.6, 143.5, 140.0, 130.7, 129.6,

121.3, 118.7, 108.4, 104.8, 32.1. HRMS (ESI) m/z: calculated for $C_{12}H_{10}N_4ONa$ [M + Na]$^+$ 249.0752, found 249.0750.

(1-allyl-1H-indol-3-yl)(1H-pyrazol-1-yl)methanone

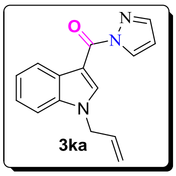

Purified by column chromatography (10:1 PE/EA); light red solid; 56% yield; *Rf* = 0.47 (5:1 PE/EA). ^{1}H NMR (400 MHz, DMSO) δ 8.98 (s, 1H), 8.58 (d, *J* = 2.7 Hz, 1H), 8.40–8.28 (m, 1H), 7.96 (d, *J* = 0.7 Hz, 1H), 7.67–7.58 (m, 1H), 7.39–7.30 (m, 2H), 6.65 (dd, *J* = 2.7, 1.5 Hz, 1H), 6.08–6.04 (m, 1H), 5.24 (dd, *J* = 10.3, 1.3 Hz, 1H), 5.18–5.13 (m, 1H), 5.04 (d, *J* = 5.5 Hz, 2H). ^{13}C NMR (100 MHz, DMSO) δ 160.2, 143.8, 139.8, 135.9, 133.4, 129.7, 128.2, 123.2, 122.6, 121.4, 117.9, 111.4, 108.7, 104.9, 48.8. HRMS (ESI) m/z: calculated for $C_{15}H_{13}N_3ONa$ [M + Na]$^+$ 274.0956, found 274.0956.

(1-benzyl-1H-indol-3-yl)(1H-pyrazol-1-yl)methanone

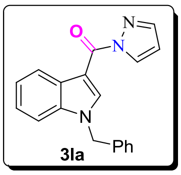

Purified by column chromatography (10:1 PE/EA); white solid; 56% yield; *Rf* = 0.40 (5:1 PE/EA). ^{1}H NMR (400 MHz, $CDCl_3$) δ 8.97 (s, 1H), 8.52 (t, *J* = 5.1 Hz, 2H), 7.74 (s, 1H), 7.37–7.25 (m, 6H), 7.19–7.11 (m, 2H), 6.46 (dd, *J* = 2.5, 1.4 Hz, 1H), 5.41 (s, 2H). ^{13}C NMR (100 MHz, $CDCl_3$) δ 160.9, 143.2, 139.8, 136.3, 135.8, 129.7, 129.1, 129.0, 128.1, 126.8, 123.6, 122.9, 122.5, 110.5, 108.1, 106.7, 51.1. HRMS (ESI) m/z: calculated for $C_{19}H_{15}N_3ONa$ [M + Na]$^+$ 324.1112, found 324.1111.

(1-methyl-1H-pyrrol-3-yl)(1H-pyrazol-1-yl)methanone

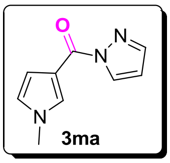

Purified by column chromatography (10:1 PE/EA); light red solid; 62% yield; *Rf* = 0.49 (5:1 PE/EA). ^{1}H NMR (400 MHz, $CDCl_3$) δ 8.38 (d, *J* = 2.4 Hz, 1H), 7.80–7.63 (m, 2H), 6.96 (s, 1H), 6.46 (dd, *J* = 2.7, 1.5 Hz, 1H), 6.24 (dd, *J* = 4.2, 2.5 Hz, 1H), 4.01 (s, 3H). ^{13}C NMR (100 MHz, $CDCl_3$) δ 157.7, 143.5, 132.3, 129.9, 124.6, 122.6, 109.0, 108.3, 37.7. HRMS (ESI) m/z: calculated for $C_9H_9N_3ONa$ $[M + Na]^+$ 198.0643, found 198.0632.

(1-methyl-1H-indol-3-yl)(3-methyl-1H-pyrazol-1-yl)methanone

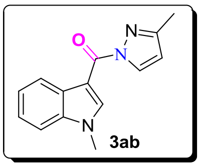

Purified by column chromatography (10:1 PE/EA); white solid; 72% yield; *Rf* = 0.36 (5:1 PE/EA). ^{1}H NMR (400 MHz, $CDCl_3$) δ 8.84 (s, 1H), 8.54–8.45 (m, 1H), 8.39 (d, *J* = 2.5 Hz, 1H), 7.44–7.29 (m, 3H), 6.26 (d, *J* = 2.5 Hz, 1H), 3.89 (s, 3H), 2.40 (s, 3H). ^{13}C NMR (100 MHz, $CDCl_3$) δ 160.8, 153.0, 139.8, 136.8, 130.4, 128.9, 123.3, 122.7, 122.4, 109.7, 108.6, 106.3, 33.7, 14.1. HRMS (ESI) m/z: calculated for $C_{14}H_{13}N_3ONa$ $[M + Na]^+$ 262.0956, found 262.0954.

(3,5-dimethyl-1H-pyrazol-1-yl)(1-methyl-1H-indol-3-yl)methanone

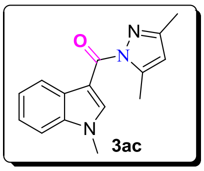

Purified by column chromatography (10:1 PE/EA); light red solid; 63% yield; *Rf* = 0.35 (5:1 PE/EA). ^{1}H NMR (400 MHz, $CDCl_3$) δ 8.60 (s, 1H), 8.46 (dd, *J* = 6.1, 2.7 Hz, 1H), 7.33 (dt, *J* = 5.5, 3.9 Hz, 3H), 6.01 (s, 1H), 3.86 (s, 3H), 2.66 (s, 3H), 2.32 (s, 3H). ^{13}C NMR (100 MHz, $CDCl_3$) δ 163.2, 150.9, 144.7, 139.7, 136.8, 128.9, 123.1, 122.5, 122.4, 110.0, 109.6, 107.6, 33.6, 14.7, 13.9. HRMS (ESI) m/z: calculated for $C_{15}H_{15}N_3ONa$ $[M + Na]^+$ 276.1112, found 276.1092.

(1H-imidazol-1-yl)(1-methyl-1H-indol-3-yl)methanone

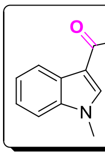
3ad

Purified by column chromatography (2:1 PE/EA); white solid; 85% yield; *Rf* = 0.06 (2:1 PE/EA). ^{1}H NMR (400 MHz, $CDCl_3$) δ 8.19 – 8.02 (m, 2H), 7.55 (d, *J* = 26.8 Hz, 2H), 7.36 – 7.22 (m, 3H), 7.07 (s, 1H), 3.80 (s, 3H). ^{13}C NMR (101 MHz, $CDCl_3$) δ 160.73 (s), 137.38 (d, *J* = 7.5 Hz), 136.05 (s), 130.31 (s), 127.29 (s), 124.26 (s), 123.10 (s), 121.72 (s), 118.02 (s), 110.22 (s), 107.94 (s), 33.86 (s). HRMS (ESI) m/z: calculated for $C_{13}H_{11}N_3ONa$ $[M + Na]^+$ 248.0800, found 248.0802.

(1H-benzo[d]imidazol-1-yl)(1-methyl-1H-indol-3-yl)methanone

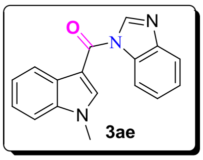
3ae

Purified by column chromatography (2:1 PE/EA); white solid; 68% yield; *Rf* = 0.14 (2:1 PE/EA). ^{1}H NMR (400 MHz, $CDCl_3$) δ 8.39 (s, 1H), 8.08 (dd, *J* = 6.4, 2.5 Hz, 1H), 8.03 (d, *J* = 7.5 Hz, 1H), 7.75 (dd, *J* = 6.2, 2.3 Hz, 1H), 7.57 (s, 1H), 7.36–7.21 (m, 5H), 3.80 (s, 3H). ^{13}C NMR (100 MHz, $CDCl_3$) δ 160.7, 143.1, 141.7, 136.4, 134.8, 131.5, 127.8, 124.1, 123.5, 123.1, 121.9, 120.5, 119.3, 113.9, 109.2, 107.7, 32.8. HRMS (ESI) m/z: calculated for $C_{17}H_{13}N_3ONa$ $[M + Na]^+$ 298.0956, found 298.0954.

(1H-benzo[d][1,2,3]triazol-1-yl)(1-methyl-1H-indol-3-yl)methanone

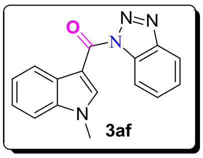
3af

Purified by column chromatography (10:1 then 5:1 PE/EA); white solid; 71% yield; *Rf* = 0.20 (5:1 PE/EA). ^{1}H NMR (400 MHz, $CDCl_3$) δ 8.76 (s, 1H), 8.57–8.52 (m, 1H), 8.49 (d, *J* = 8.3 Hz, 1H), 8.12 (d, *J* = 8.3 Hz, 1H), 7.63 (dd, *J* = 11.4, 4.0 Hz, 1H), 7.53–7.44 (m, 1H), 7.43–7.31 (m, 3H), 3.90 (d, *J* = 1.3 Hz, 3H). ^{13}C NMR (100 MHz, $CDCl_3$) δ 160.9, 145.6, 139.8, 137.0, 132.6, 129.8, 128.6, 125.7, 123.8, 123.2, 122.3, 119.8, 115.1, 110.0, 106.4, 33.8. The spectral data obtained agree with those reported in the literature.[10]

(1H-indazol-1-yl)(1-methyl-1H-indol-3-yl)methanone

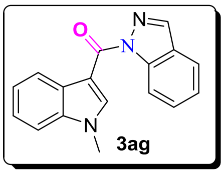
3ag

Purified by column chromatography (10:1 PE/EA); light red solid; 67% yield; *Rf* = 0.30 (5:1 PE/EA). ^{1}H NMR (400 MHz, $CDCl_3$) δ 8.74 (s, 1H), 8.70 (dd, *J* = 8.5, 0.7 Hz, 1H), 8.63–8.57 (m, 1H), 8.18 (d, *J* = 0.5 Hz, 1H), 7.76 (d, *J* = 8.0 Hz, 1H), 7.58 (ddd, *J* = 8.3, 7.1, 1.1 Hz, 1H), 7.39–7.31 (m, 4H), 3.90 (s, 3H). ^{13}C NMR (100 MHz, $CDCl_3$) δ 163.3, 140.4, 138.9, 138.7, 136.7, 129.1, 129.0, 125.6, 123.9, 123.2, 122.5 (two peaks overlapped), 120.7, 116.1, 109.7, 107.4, 33.6. HRMS (ESI) m/z: calculated for $C_{17}H_{13}N_3ONa$ $[M + Na]^+$ 298.0956, found 298.0956.

1-methyl-N-phenyl-1H-indole-3-carboxamide

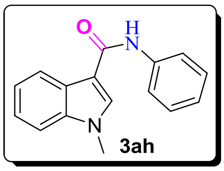
3ah

Purified by column chromatography (10:1 then 5:1 PE/EA); white solid; 64% yield; *Rf* = 0.38 (2:1 PE/EA). ^{1}H NMR (400 MHz, $CDCl_3$) δ 8.05 (dd, *J* = 6.3, 2.5 Hz, 1H), 7.74 (d, *J* = 10.5 Hz, 2H), 7.66 (d, *J* = 7.7 Hz, 2H), 7.40–7.28 (m, 5H), 7.11 (t, *J* = 7.4 Hz, 1H), 3.80 (s, 3H). ^{13}C NMR (100 MHz, $CDCl_3$) δ 163.2, 138.5, 137.4, 132.6, 130.9, 129.1, 128.9, 125.4, 123.9, 122.8, 121.8, 120.1 (two peaks overlapped), 111.1, 110.3, 33.4. The spectral data obtained agree with those reported in the literature.[11]

N-(4-methoxyphenyl)-1-methyl-1H-indole-3-carboxamide

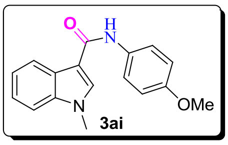
3ai

Purified by column chromatography (10:1 then 5:1 PE/EA); white solid; 70% yield; *Rf* = 0.05 (5:1 PE/EA). ^{1}H NMR (400 MHz, $CDCl_3$) δ 8.05 (dd, *J* = 6.5, 1.9 Hz, 1H), 7.71 (s, 2H), 7.55 (d, *J* = 8.9 Hz, 2H), 7.44–7.27 (m, 3H), 6.93–6.86 (m, 2H), 3.80, 3.79 (6H). ^{13}C NMR (100 MHz, $CDCl_3$) δ 163.3, 156.3, 137.3, 132.5,

131.5, 130.9, 128.9, 125.5, 122.8, 122.1, 121.7, 120.2, 114.2, 111.0, 110.2, 55.5, 33.3. The spectral data obtained agree with those reported in the literature.[12]

N-(4-chlorophenyl)-1-methyl-1H-indole-3-carboxamide

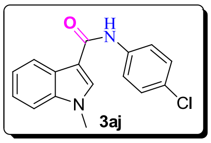

Purified by column chromatography (10:1 then 5:1 PE/EA); white solid; 59% yield; *Rf* = 0.06 (5:1 PE/EA). ^{1}H NMR (400 MHz, $CDCl_3$) δ 8.03 (dd, *J* = 6.4, 2.0 Hz, 1H), 7.76–7.68 (m, 2H), 7.61 (d, *J* = 8.8 Hz, 2H), 7.42–7.37 (m, 1H), 7.37–7.28 (m, 4H), 3.82 (s, 3H). ^{13}C NMR (100 MHz, $CDCl_3$) δ 163.1, 137.4, 137.1, 132.7, 130.9, 129.0, 128.9, 128.8, 125.3, 122.9, 121.9, 121.2, 120.1, 110.8, 110.3, 33.4. HRMS (ESI) m/z: calculated for $C_{16}H_{13}ClN_2ONa$ $[M + Na]^+$ 307.0614, found 307.0593.

1-methyl-1H-indole-3-carbonyl azide

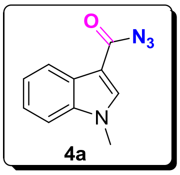

Purified by column chromatography (10:1 PE/EA); light red solid; 86% yield; *Rf* = 0.30 (5:1 PE/EA). ^{1}H NMR (400 MHz, $CDCl_3$) δ 8.20–8.09 (m, 1H), 7.63 (s, 1H), 7.29–7.20 (m, 3H), 3.68 (s, 3H). ^{13}C NMR (100 MHz, $CDCl_3$) δ 167.8, 137.6, 136.5, 126.5, 123.5, 122.7, 121.7, 110.1, 108.4, 33.6. The spectral data obtained agree with those reported in the literature.[12]

1,5-dimethyl-1H-indole-3-carbonyl azide

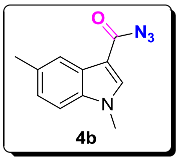

Purified by column chromatography (10:1 PE/EA); light red solid; 85% yield; *Rf* = 0.35 (5:1 PE/EA). ^{1}H NMR (400 MHz, $CDCl_3$) δ 7.93 (s, 1H), 7.57 (s, 1H), 7.07 (dd, *J* = 26.7, 8.4 Hz, 2H), 3.64 (s, 3H), 2.39 (s, 3H). ^{13}C NMR (100 MHz, $CDCl_3$) δ 167.8, 136.4, 136.0, 132.5, 126.7, 125.0, 121.4, 109.7, 107.9, 33.7, 21.6. HRMS (ESI) m/z: calculated for $C_{11}H_{11}N_2O$ $[M + H – N_2]^+$ 187.0871, found 187.0869.

1,6-dimethyl-1H-indole-3-carbonyl azide

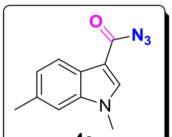

4c

Purified by column chromatography (10:1 PE/EA); light red solid; 68% yield; *Rf* = 0.31 (5:1 PE/EA). ^{1}H NMR (400 MHz, DMSO) δ 8.13 (s, 1H), 7.93 (d, *J* = 8.1 Hz, 1H), 7.37 (s, 1H), 7.12 (d, *J* = 8.1 Hz, 1H), 3.83 (s, 3H), 2.45 (s, 3H). ^{13}C NMR (100 MHz, DMSO) δ 166.7, 137.9, 137.5, 132.6, 124.1, 123.6, 120.2, 110.9, 106.6, 33.2, 21.3. HRMS (ESI) m/z: calculated for $C_{11}H_{11}N_2O$ [M + H – N_2]$^+$ 187.0871, found 187.0868.

1,7-dimethyl-1H-indole-3-carbonyl azide

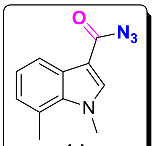

4d

Purified by column chromatography (10:1 PE/EA); light red solid; 92% yield; *Rf* = 0.38 (5:1 PE/EA). ^{1}H NMR (400 MHz, DMSO) δ 8.12 (s, 1H), 7.93 (d, *J* = 7.9 Hz, 1H), 7.12 (t, *J* = 7.6 Hz, 1H), 7.00 (d, *J* = 7.2 Hz, 1H), 4.10 (s, 3H), 2.72 (s, 3H). ^{13}C NMR (100 MHz, DMSO) δ 166.6, 139.3, 136.1, 127.0, 125.7, 122.8, 122.6, 118.6, 106.1, 37.3, 18.8. HRMS (ESI) m/z: calculated for $C_{11}H_{11}N_2O$ [M + H – N_2]$^+$ 187.0871, found 187.0868.

1,2-dimethyl-1H-indole-3-carbonyl azide

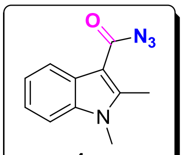

4e

Purified by column chromatography (10:1 PE/EA); light red solid; 71% yield; *Rf* = 0.39 (5:1 PE/EA). ^{1}H NMR (400 MHz, DMSO) δ 8.12–8.03 (m, 1H), 7.59–7.50 (m, 1H), 7.27–7.18 (m, 2H), 3.72 (s, 3H), 2.73 (s, 3H). ^{13}C NMR (100 MHz, DMSO) δ 167.7, 147.9, 136.5, 125.5, 122.3, 122.0, 120.5, 110.4, 104.0, 29.8, 12.0. HRMS (ESI) m/z: calculated for $C_{11}H_{10}N_2ONa$ [M + Na – N_2]$^+$ 209.0690, found 209.0684.

5-methoxy-1-methyl-1H-indole-3-carbonyl azide

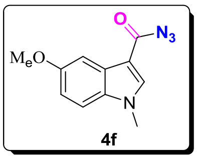

Purified by column chromatography (10:1 PE/EA); light red solid; 66% yield; *Rf* = 0.23 (5:1 PE/EA). ^{1}H NMR (400 MHz, DMSO) δ 8.12 (s, 1H), 7.53 (d, *J* = 2.4 Hz, 1H), 7.47 (d, *J* = 8.9 Hz, 1H), 6.94 (dd, *J* = 8.9, 2.5 Hz, 1H), 3.83 (s, 3H), 3.81 (s, 3H). ^{13}C NMR (100 MHz, DMSO) δ 166.7, 155.9, 137.8, 132.5, 126.7, 112.8, 112.1, 106.2, 102.3, 55.3, 33.4. HRMS (ESI) m/z: calculated for $C_{11}H_{11}N_2O_2$ $[M + H – N_2]^+$ 203.0820, found 203.0809.

5-cyano-1-methyl-1H-indole-3-carbonyl azide

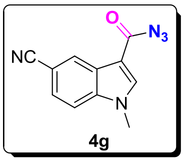

Purified by column chromatography (10:1 PE/EA); light red solid; 49% yield; *Rf* = 0.11 (5:1 PE/EA). ^{1}H NMR (400 MHz, DMSO) δ 8.46 (s, 1H), 8.40 (d, *J* = 0.7 Hz, 1H), 7.82 (d, *J* = 8.6 Hz, 1H), 7.72 (dd, *J* = 8.6, 1.2 Hz, 1H), 3.93 (s, 3H). ^{13}C NMR (100 MHz, DMSO) δ 166.9, 140.2, 139.2, 126.0, 125.4 (two peaks overlapped), 119.8, 112.9, 107.3, 104.7, 33.6. HRMS (ESI) m/z: calculated for $C_{11}H_8N_3O$ $[M + H – N_2]^+$ 198.0667, found 198.0662.

6-bromo-1-methyl-1H-indole-3-carbonyl azide

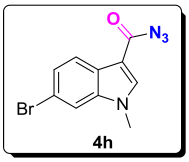

Purified by column chromatography (10:1 PE/EA); light red solid; 61% yield; *Rf* = 0.43 (5:1 PE/EA). ^{1}H NMR (400 MHz, DMSO) δ 8.21 (s, 1H), 7.94 (d, *J* = 8.5 Hz, 1H), 7.85 (d, *J* = 1.5 Hz, 1H), 7.40 (dd, *J* = 8.5, 1.7 Hz, 1H), 3.85 (s, 3H). ^{13}C NMR (100 MHz, DMSO) δ 166.8, 138.6, 138.4, 125.3, 124.7, 122.1, 115.9, 114.2, 106.8, 33.5. HRMS (ESI) m/z: calculated for $C_{10}H_8BrN_2O$ $[M + H – N_2]^+$ 250.9820, found 250.9821.

6-chloro-1-methyl-1H-indole-3-carbonyl azide

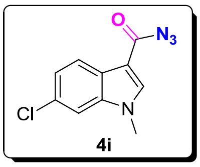

Purified by column chromatography (10:1 PE/EA); light red solid; 68% yield; *Rf* = 0.30 (5:1 PE/EA). ^{1}H NMR (400 MHz, DMSO) δ 8.24 (s, 1H), 8.01 (d, *J* = 8.5 Hz, 1H), 7.73 (d, *J* = 1.4 Hz, 1H), 7.30 (dd, *J* = 8.5, 1.5 Hz, 1H), 3.86 (s, 3H). ^{13}C NMR (100 MHz, DMSO) δ 166.8, 138.8, 138.0, 127.9, 124.4, 122.7, 121.8, 111.3, 106.8, 33.5. HRMS (ESI) m/z: calculated for $C_{10}H_8BrN_2O$ $[M + H – N_2]^+$ 207.0325, found 207.0316.

1-methyl-1H-pyrrolo[2,3-*b*]pyridine-3-carbonyl azide

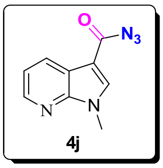

Purified by column chromatography (10:1 PE/EA); light red solid; 69% yield; *Rf* = 0.15 (5:1 PE/EA). ^{1}H NMR (400 MHz, DMSO) δ 8.48–8.39 (m, 2H), 8.36 (dd, *J* = 7.9, 1.3 Hz, 1H), 7.35 (dd, *J* = 7.9, 4.7 Hz, 1H), 3.90 (s, 3H). ^{13}C NMR (100 MHz, DMSO) δ 166.8, 148.0, 144.3, 138.1, 129.0, 118.6, 118.1, 105.2, 31.6. HRMS (ESI) m/z: calculated for $C_9H_7N_3ONa$ $[M + Na – N_2]^+$ 196.0487, found 196.0486.

1-allyl-1H-indole-3-carbonyl azide

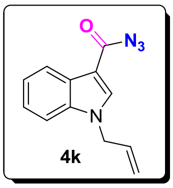

Purified by column chromatography (10:1 PE/EA); light red solid; 66% yield; *Rf* = 0.6 (5:1 PE/EA). ^{1}H NMR (400 MHz, DMSO) δ 8.26 (s, 1H), 8.09 (dt, *J* = 5.0, 2.8 Hz, 1H), 7.59 (dd, *J* = 6.0, 2.8 Hz, 1H), 7.36–7.23 (m, 2H), 6.03 (dq, *J* = 10.5, 5.6 Hz, 1H), 5.22 (dd, *J* = 10.3, 1.3 Hz, 1H), 5.11 (dd, *J* = 17.1, 1.5 Hz, 1H), 4.95 (d, *J* = 5.5 Hz, 2H). ^{13}C NMR (100 MHz, DMSO) δ 166.9, 137.2, 136.8, 133.2, 125.9, 123.2, 122.6, 120.7, 117.9, 111.6, 107.1, 48.7. HRMS (ESI) m/z: calculated for $C_{12}H_{11}N_2O$ $[M + H – N_2]^+$ 199.0871, found 199.0869.

1-allyl-5-methyl-1H-indole-3-carbonyl azide

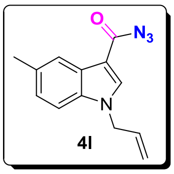

Purified by column chromatography (10:1 PE/EA); light red solid; 83% yield; *Rf* = 0.34 (5:1 PE/EA). ^{1}H NMR (400 MHz, DMSO) δ 8.19 (s, 1H), 7.89 (s, 1H), 7.45 (d, *J* = 8.4 Hz, 1H), 7.12 (dd, *J* = 8.4, 1.2 Hz, 1H), 6.02 (m, 1H), 5.20 (dd, *J* = 10.3, 1.3 Hz, 1H), 5.09 (dd, *J* = 17.1, 1.4 Hz, 1H), 4.91 (d, *J* = 5.5 Hz, 2H), 2.43 (s, 3H). ^{13}C NMR (100 MHz, DMSO) δ 166.8, 137.0, 135.2, 133.3, 131.6, 126.2, 124.7, 120.4, 117.7, 111.3, 106.7, 48.7, 21.2. HRMS (ESI) m/z: calculated for $C_{13}H_{13}N_2O$ [M + H – N_2]$^+$ 213.1028, found 213.1027.

1-allyl-6-bromo-1H-indole-3-carbonyl azide

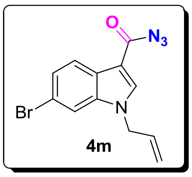

Purified by column chromatography (10:1 PE/EA); light red solid; 61% yield; *Rf* = 0.44 (5:1 PE/EA). ^{1}H NMR (400 MHz, DMSO) δ 8.30 (s, 1H), 8.18 (d, *J* = 1.9 Hz, 1H), 7.57 (d, *J* = 8.8 Hz, 1H), 7.44 (dd, *J* = 8.8, 2.0 Hz, 1H), 6.02 (qd, *J* = 10.6, 5.5 Hz, 1H), 5.22 (dd, *J* = 10.3, 1.3 Hz, 1H), 5.09 (dd, *J* = 17.1, 1.4 Hz, 1H), 4.95 (d, *J* = 5.5 Hz, 2H). ^{13}C NMR (100 MHz, DMSO) δ 166.9, 138.1, 135.6, 133.0, 127.5, 125.8, 122.8, 118.0, 115.4, 113.8, 106.7, 48.9. HRMS (ESI) m/z: calculated for $C_{12}H_{10}BrN_2O$ [M + H – N_2]$^+$ 276.9976, found 276.9971.

methyl 1-allyl-3-(azidocarbonyl)-1H-indole-5-carboxylate

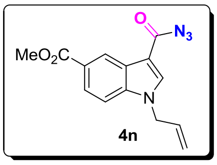

Purified by column chromatography (10:1 then 5:1 PE/EA); light red solid; 47% yield; *Rf* = 0.29 (5:1 PE/EA). 1H NMR (400 MHz, DMSO) δ 8.76–8.70 (m, 1H), 8.38 (s, 1H), 7.90 (dd, *J* = 8.7, 1.7 Hz, 1H), 7.69 (d, *J* = 8.7 Hz, 1H), 6.04 (qd, *J* = 10.6, 5.5 Hz, 1H), 5.23 (dd, *J* = 10.3, 1.3 Hz, 1H), 5.11 (dd, *J* = 17.1, 1.4 Hz, 1H), 4.99

(d, J = 5.5 Hz, 2H), 3.89 (s, 3H). ^{13}C NMR (100 MHz, DMSO) δ 167.0, 166.6, 139.2, 138.9, 133.0, 125.4, 123.9 (two peaks overlapped), 122.7, 118.1, 111.8, 108.2, 52.0, 48.9. HRMS (ESI) m/z: calculated for $C_{14}H_{13}N_2O_3$ [M + H – N_2]$^+$ 257.0926, found 257.0923.

1-benzyl-1H-indole-3-carbonyl azide

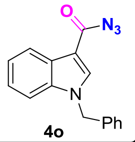

4o

Purified by column chromatography (10:1 PE/EA); light red solid; 81% yield; *Rf* = 0.42 (5:1 PE/EA). ^{1}H NMR (400 MHz, DMSO) δ 8.46 (s, 1H), 8.09 (dd, J = 6.4, 2.8 Hz, 1H), 7.60 (dd, J = 6.7, 2.6 Hz, 1H), 7.42–7.20 (m, 7H), 5.55 (s, 2H). ^{13}C NMR (100 MHz, DMSO) δ 167.0, 137.4, 136.7, 136.6, 128.7, 127.8, 127.4, 126.1, 123.3, 122.6, 120.7, 111.7, 107.3, 49.8. The spectral data obtained agree with those reported in the literature.[13]

1-methyl-1H-indole-3-carbonyl cyanide

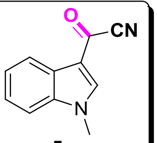

5a

Purified by column chromatography (10:1 then 5:1 PE/EA); light red solid; 82% yield; *Rf* = 0.24 (5:1 PE/EA). ^{1}H NMR (400 MHz, DMSO) δ 8.69 (s, 1H), 8.05 (d, J = 7.3 Hz, 1H), 7.67 (d, J = 7.9 Hz, 1H), 7.47–7.34 (m, 2H), 3.97 (s, 3H). ^{13}C NMR (100 MHz, DMSO) δ 157.8, 144.2, 138.3, 124.9, 124.5, 124.2, 121.0, 115.0, 114.3, 111.9, 33.9. The spectral data obtained agree with those reported in the literature.[14]

1,4-dimethyl-1H-indole-3-carbonyl cyanide

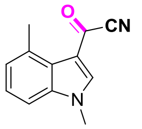

5b

Purified by column chromatography (10:1 then 5:1 PE/EA); light red solid; 81% yield; *Rf* = 0.23 (5:1 PE/EA). 1H NMR (400 MHz, $CDCl_3$) δ 8.06 (s, 1H), 7.29–

7.20 (m, 1H), 7.14 (d, *J* = 8.2 Hz, 1H), 7.07 (d, *J* = 7.3 Hz, 1H), 3.84 (s, 3H), 2.78 (s, 3H). ^{13}C NMR (100 MHz, $CDCl_3$) δ 158.2, 144.4, 139.4, 134.1, 126.3, 125.5, 124.4, 117.6, 115.0, 107.9, 34.4, 22.8. GCMS (EI): m/z for $C_{12}H_{10}N_2O$ $[M]^+$ 198.1.

1,5-dimethyl-1H-indole-3-carbonyl cyanide

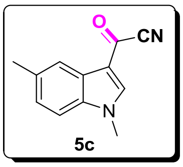

Purified by column chromatography (10:1 PE/EA); light red solid; 85% yield; *Rf* = 0.25 (5:1 PE/EA). ^{1}H NMR (400 MHz, DMSO) δ 8.63 (s, 1H), 7.86 (s, 1H), 7.56 (d, *J* = 8.4 Hz, 1H), 7.26 (d, *J* = 8.3 Hz, 1H), 3.94 (s, 3H), 2.44 (s, 3H). ^{13}C NMR (100 MHz, DMSO) δ 162.89 (s), 149.22 (s), 141.94 (s), 138.93 (s), 131.53 (s), 130.10 (s), 126.12 (s), 119.98 (s), 119.63 (s), 116.82 (s), 45.20 (s), 44.99 (s), 44.78 (s), 44.57 (s), 44.36 (s), 26.34 (s). GCMS (EI): m/z for $C_{12}H_{10}N_2O$ $[M]^+$ 198.1.

1,6-dimethyl-1H-indole-3-carbonyl cyanide

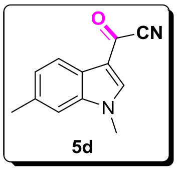

Purified by column chromatography (10:1 then 5:1 PE/EA); light red solid; 71% yield; *Rf* = 0.27 (5:1 PE/EA). ^{1}H NMR (400 MHz, DMSO) δ 8.57 (s, 1H), 7.88 (d, *J* = 8.0 Hz, 1H), 7.44 (s, 1H), 7.19 (d, *J* = 8.0 Hz, 1H), 3.91 (s, 3H), 2.45 (s, 3H). ^{13}C NMR (100 MHz, DMSO) δ 157.6, 143.8, 138.6, 134.6, 125.6, 122.3, 120.7, 115.0, 114.3, 111.6, 33.7, 21.3. GCMS (EI): m/z for $C_{12}H_{10}N_2O$ $[M]^+$ 198.1.

1,7-dimethyl-1H-indole-3-carbonyl cyanide

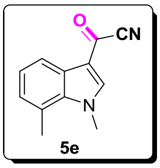

Purified by column chromatography (10:1 then 5:1 PE/EA); light red solid; 83% yield; *Rf* = 0.22 (5:1 PE/EA). ^{1}H NMR (400 MHz, DMSO) δ 8.56 (s, 1H), 7.89 (d, *J* = 7.7 Hz, 1H), 7.21 (t, *J* = 7.6 Hz, 1H), 7.11 (d, *J* = 7.2 Hz, 1H), 4.18 (s, 3H), 2.73 (s,

3H). ^{13}C NMR (100 MHz, DMSO) δ 157.5, 145.2, 136.8, 127.5, 125.7, 124.3, 123.6, 119.1, 114.5, 114.3, 37.8, 18.6. GCMS (EI): m/z for $C_{12}H_{10}N_2O$ $[M]^+$ 198.1.

1,2-dimethyl-1H-indole-3-carbonyl cyanide

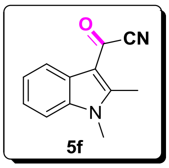

Purified by column chromatography (10:1 then 5:1 PE/EA); light red solid; 60% yield; *Rf* = 0.17 (5:1 PE/EA). ^{1}H NMR (400 MHz, DMSO) δ 8.13 (dd, *J* = 5.9, 3.0 Hz, 1H), 7.67 (dd, *J* = 6.1, 2.9 Hz, 1H), 7.39–7.31 (m, 2H), 3.80 (s, 3H), 3.36 (s, 3H). ^{13}C NMR (100 MHz, $CDCl_3$) δ 163.0, 157.0, 142.1, 130.2, 129.2, 129.1, 124.8, 120.8, 116.5, 35.6, 17.3. GCMS (EI): m/z for $C_{12}H_{10}N_2O$ $[M]^+$ 198.1.

5-methoxy-1-methyl-1H-indole-3-carbonyl cyanide

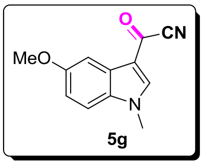

Purified by column chromatography (10:1 then 5:1 PE/EA); light red solid; 79% yield; *Rf* = 0.16 (5:1 PE/EA). ^{1}H NMR (400 MHz, DMSO) δ 8.59 (s, 1H), 7.57 (d, *J* = 8.9 Hz, 1H), 7.49 (s, 1H), 7.03 (dd, *J* = 8.8, 1.6 Hz, 1H), 3.93 (s, 3H), 3.81 (s, 3H). ^{13}C NMR (100 MHz, DMSO) δ 157.5, 157.1, 143.6, 133.0, 125.6, 114.8, 114.3, 114.2, 112.9, 103.1, 55.4, 34.0. GCMS (EI): m/z for $C_{12}H_{10}N_2O_2$ $[M]^+$ 214.1.

6-bromo-1-methyl-1H-indole-3-carbonyl cyanide

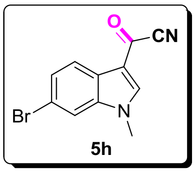

Purified by column chromatography (10:1 then 5:1 PE/EA); light red solid; 63% yield; *Rf* = 0.23 (5:1 PE/EA). ^{1}H NMR (400 MHz, DMSO) δ 8.72 (s, 1H), 7.98–7.93 (m, 2H), 7.52 (dd, *J* = 8.4, 1.6 Hz, 1H), 3.96 (s, 3H). ^{13}C NMR (100 MHz, DMSO) δ 158.0, 144.7, 139.1, 127.0, 123.5, 122.5, 117.5, 115.0, 114.8, 114.1, 34.0. GCMS (EI): m/z for $C_{11}H_7BrN_2O$ $[M]^+$ 262.0.

6-chloro-1-methyl-1H-indole-3-carbonyl cyanide

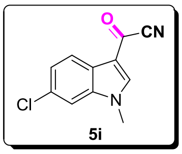

Purified by column chromatography (10:1 then 5:1 PE/EA); light red solid; 71% yield; *Rf* = 0.20 (5:1 PE/EA). ^{1}H NMR (400 MHz, DMSO) δ 8.73 (s, 1H), 7.99 (d, *J* = 8.4 Hz, 1H), 7.84 (d, *J* = 1.7 Hz, 1H), 7.39 (dd, *J* = 8.4, 1.8 Hz, 1H), 3.95 (s, 3H). ^{13}C NMR (100 MHz, DMSO) δ 158.0, 144.9, 138.8, 129.5, 124.4, 123.2, 122.2, 114.8, 114.1, 112.2, 34.0. GCMS (EI): m/z for $C_{11}H_7ClN_2O$ $[M]^+$ 218.0.

1-methyl-1H-pyrrolo[2,3-b]pyridine-3-carbonyl cyanide

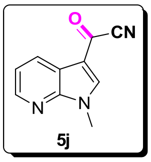

Purified by column chromatography (10:1 then 5:1 PE/EA); light red solid; 55% yield; *Rf* = 0.12 (5:1 PE/EA). ^{1}H NMR (400 MHz, DMSO) δ 8.95 (s, 1H), 8.52 (dd, *J* = 4.7, 1.3 Hz, 1H), 8.39 (dd, *J* = 7.8, 1.3 Hz, 1H), 7.45 (dd, *J* = 7.8, 4.7 Hz, 1H), 3.98 (s, 3H). ^{13}C NMR (100 MHz, DMSO) δ 158.4, 148.8, 145.9, 144.3, 129.7, 120.1, 117.1, 113.9, 113.1, 32.2. GCMS (EI): m/z for $C_{10}H_7N_3O$ $[M]^+$ 185.1.

1-allyl-1H-indole-3-carbonyl cyanide

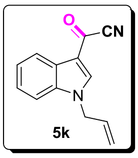

Purified by column chromatography (10:1 PE/EA); light red solid; 67% yield; *Rf* = 0.36 (5:1 PE/EA). ^{1}H NMR (400 MHz, DMSO) δ 8.73 (s, 1H), 8.12–7.99 (m, 1H), 7.67 (d, *J* = 7.7 Hz, 1H), 7.42–7.38 (m, 2H), 6.11–6.04 (m, 1H), 5.31–5.16 (m, 2H), 5.06 (d, *J* = 5.6 Hz, 2H). ^{13}C NMR (100 MHz, DMSO) δ 158.1, 143.4, 137.5, 132.7, 125.0, 124.7, 124.2, 121.2, 118.5, 115.4, 114.3, 112.3, 49.3. GCMS (EI): m/z for $C_{13}H_{10}N_2O$ $[M]^+$ 210.1.

1-allyl-5-methyl-1H-indole-3-carbonyl cyanide

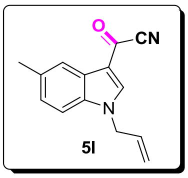

Purified by column chromatography (10:1 PE/EA); light red solid; 75% yield; *Rf* = 0.34 (5:1 PE/EA). ^{1}H NMR (400 MHz, $CDCl_3$) δ 8.06, 8.02 (s, 2H), 7.33–7.18 (m, 2H), 6.07–6.00 (m, 1H), 5.40 (d, *J* = 10.2 Hz, 1H), 5.27 (d, *J* = 17.1 Hz, 1H), 4.81 (d, *J* = 5.6 Hz, 2H), 2.49 (s, 3H). ^{13}C NMR (100 MHz, $CDCl_3$) δ 158.7, 140.5, 136.0, 134.6, 130.8, 126.8, 125.6, 122.4, 120.1, 116.6, 114.1, 110.6, 50.1, 21.5. GCMS (EI): m/z for $C_{14}H_{12}N_2O$ $[M]^+$ 224.1.

1-allyl-6-bromo-1H-indole-3-carbonyl cyanide

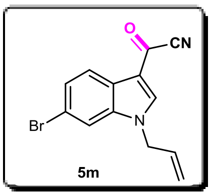

Purified by column chromatography (10:1 PE/EA); light red solid; 57% yield; *Rf* = 0.40 (5:1 PE/EA). ^{1}H NMR (400 MHz, $CDCl_3$) δ 8.41 (d, *J* = 1.9 Hz, 1H), 8.06 (s, 1H), 7.49 (d, *J* = 8.8 Hz, 1H), 7.29 (dd, *J* = 8.8, 2.0 Hz, 1H), 6.07–6.00 (m, 1H), 5.44 (dd, *J* = 10.3, 1.3 Hz, 1H), 5.30 (dd, *J* = 17.1, 1.4 Hz, 1H), 4.83 (d, *J* = 5.6 Hz, 2H). ^{13}C NMR (100 MHz, $CDCl_3$) δ 158.7, 140.7, 136.3, 130.4, 128.5, 126.8, 125.2, 120.5, 118.3, 116.3, 113.7, 112.3, 50.3. GCMS (EI): m/z for $C_{13}H_9BrN_2O$ $[M]^+$ 288.0.

1-benzyl-1H-indole-3-carbonyl cyanide

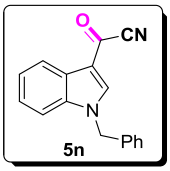

Purified by column chromatography (10:1 PE/EA); light red solid; 77% yield; *Rf* = 0.36 (5:1 PE/EA). ^{1}H NMR (400 MHz, DMSO) δ 8.98 (s, 1H), 8.14–8.02 (m, 1H), 7.66 (dd, *J* = 6.2, 2.7 Hz, 1H), 7.42–7.28 (m, 7H), 5.66 (s, 2H). ^{13}C NMR (100 MHz, DMSO) δ 158.3, 143.6, 137.4, 136.0, 128.8, 128.0, 127.5, 125.0, 124.9, 124.3, 121.3, 115.5, 114.3, 112.5, 50.3. GCMS (EI): m/z for $C_{17}H_{12}N_2O$ $[M]^+$ 260.1.

1H-indole-3-carbonyl cyanide

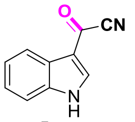

5o

Purified by column chromatography (10:1 then 5:1 PE/EA); light red solid; 59% yield; *Rf* = 0.51 (2:1 PE/EA). ^{1}H NMR (400 MHz, DMSO) δ 12.93 (s, 1H), 8.65 (s, 1H), 8.10–8.00 (m, 1H), 7.59 (d, *J* = 7.2 Hz, 1H), 7.39–7.32 (m, 2H). ^{13}C NMR (100 MHz, $CDCl_3$) δ 163.8, 146.7, 142.8, 130.2, 129.4, 129.1, 126.2, 121.4, 119.6, 118.6. The spectral data obtained agree with those reported in the literature.[14]

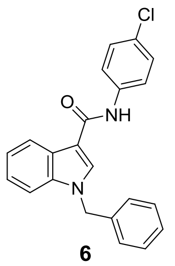

6

Purified by recrystallization from ethanol; white solid; 72% yield. ^{1}H NMR (400 MHz, $CDCl_3$) δ 8.06 (dd, *J* = 6.5, 1.9 Hz, 1H), 7.78 (s, 1H), 7.69-7.56 (m, 3H), 7.42-7.27 (m, 8H), 7.21-7.12 (m, 2H), 5.36 (s, 2H). ^{13}C NMR (100 MHz, DMSO) δ 163.3, 139.2, 137.8, 136.8, 132.5, 129.2, 128.9, 128.1, 127.6, 127.4, 123.0, 121.9, 121.7, 111.3, 110.5. HRMS (ESI) m/z: calculated for $C_{22}H_{18}ClN_2O$ $[M + H]^+$ 361.1108, found 361.1112.

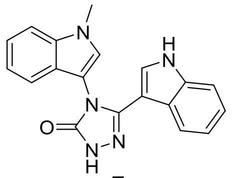

7c

Purified by column chromatography (1:2 PE/EA); white solid; 76% yield; *Rf* = 0.50 (EA). ^{1}H NMR (400 MHz, DMSO) δ11.74 (s, 1H), 10.98 (d, *J* = 1.9 Hz, 1H), 8.14-7.95 (m, 1H), 7.52 (s, 1H), 7.38 (d, *J* = 8.5 Hz, 1H), 7.19 (dd, *J* = 6.5, 1.8 Hz, 1H), 7.10-7.03 (m, 2H), 7.03-6.94 (m, 2H), 6.87 (t, *J* = 7.4 Hz, 1H), 6.42 (d, *J* = 2.8 Hz, 1H), 3.68 (s, 3H). ^{13}C NMR (100 MHz, DMSO) δ 155.6, 144.6, 136.0, 128.9, 125.4, 125.2, 124.8, 122.9, 122.6, 121.6, 120.9, 120.6, 117.9, 112.3, 111.0, 108.9, 102.7. HRMS (ESI) m/z: calculated for $C_{19}H_{16}N_5O$ $[M + H]^+$ 330.1355, found 330.1355.

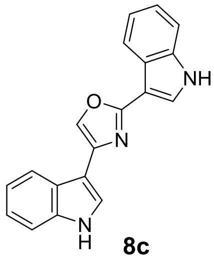

8c

Purified by column chromatography (2:1 PE/EA); yellow solid; 81% yield; *Rf* = 0.58 (3:1 PE/EA). ^{1}H NMR (400 MHz, DMSO) δ11.77 (s, 1H), 11.59 (s, 1H), 8.29 (d, *J* = 6.6 Hz, 1H), 8.14 (d, *J* = 2.2 Hz, 1H), 7.97 (d, *J* = 7.5 Hz, 1H), 7.90 (d, *J* = 1.9 Hz, 1H), 7.51 (d, *J* = 10.8 Hz, 3H), 7.35-7.10 (m, 4H). ^{13}C NMR (100 MHz, DMSO) δ 157.3, 145.9, 136.9, 123.4, 121.1, 120.5, 120.1, 112.6, 104.6. HRMS (ESI) m/z: calculated for $C_{19}H_{13}N_3ONa$ [M + Na]$^+$ 322.0956, found 322.0961.

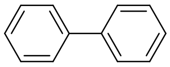

Purified by column chromatography (PE); white solid; > 98% yield; ^{1}H NMR (400 MHz, $CDCl_3$) δ7.59 (dd, *J* = 7.0, 1.5 Hz, 4H), 7.44 (td, *J* = 7.5, 1.7 Hz, 4H), 7.38-7.31 (m, 2H). ^{13}C NMR (100 MHz, $CDCl_3$) δ 141.3, 128.8, 127.3, 127.2.

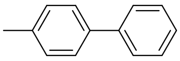

Purified by column chromatography (PE); white solid; 97% yield; ^{1}H NMR (400 MHz, $CDCl_3$) δ7.61-7.55 (m, 2H), 7.49 (d, *J* = 8.1 Hz, 2H), 7.42 (t, *J* = 7.6 Hz, 2H), 7.32 (t, *J* = 7.4 Hz, 1H), 7.27-7.24 (m, 2H), 2.39 (s, 3H). ^{13}C NMR (100 MHz, $CDCl_3$) δ 141.2, 138.4, 137.0, 129.5, 128.7, 127.0, 126.9.

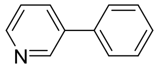

Purified by column chromatography (10:1 PE/EA); colorless oil; 94% yield; ^{1}H NMR (400 MHz, $CDCl_3$) δ8.85 (s, 1H), 8.64-8.53 (m, 1H), 7.91-7.80 (m, 1H), 7.57 (dd, *J* = 5.5, 2.0 Hz, 2H), 7.47 (ddd, *J* = 7.9, 5.5, 2.2 Hz, 2H), 7.43-7.31 (m, 2H). ^{13}C NMR (100 MHz, $CDCl_3$) δ 148.5, 148.4, 137.8, 136.7, 134.4, 129.1, 128.1, 127.2, 123.6.

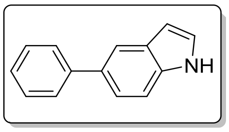 Purified by column chromatography (5:1 PE/EA); light yellow solid; 98% yield; ^{1}H NMR (400 MHz, $CDCl_3$) δ8.02 (s, 1H), 7.85 (s, 1H), 7.65 (d, *J* = 7.4 Hz, 2H), 7.48-7.36 (m, 4H), 7.30 (t, *J* = 7.4 Hz, 1H), 7.16 (t, *J* = 2.7 Hz, 1H), 6.58 (s, 1H). ^{13}C NMR (100 MHz, $CDCl_3$) δ 142.6, 135.4, 133.5, 128.7, 128.4, 127.5, 126.4, 124.9, 122.0, 119.3, 111.3, 103.1.

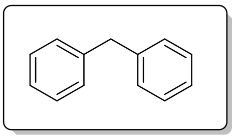 Purified by column chromatography (PE); white solid; 92% yield; ^{1}H NMR (400 MHz, $CDCl_3$) δ7.26 (dd, *J* = 10.6, 4.4 Hz, 4H), 7.16 (dd, *J* = 5.2, 1.8 Hz, 6H), 3.95 (s, 2H). ^{13}C NMR (100 MHz, $CDCl_3$) δ 141.2, 129.1, 128.6, 126.2, 42.1.

Supplementary Note 2

^{1}H NMR and ^{13}C NMR Copies of Products

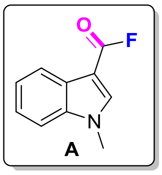

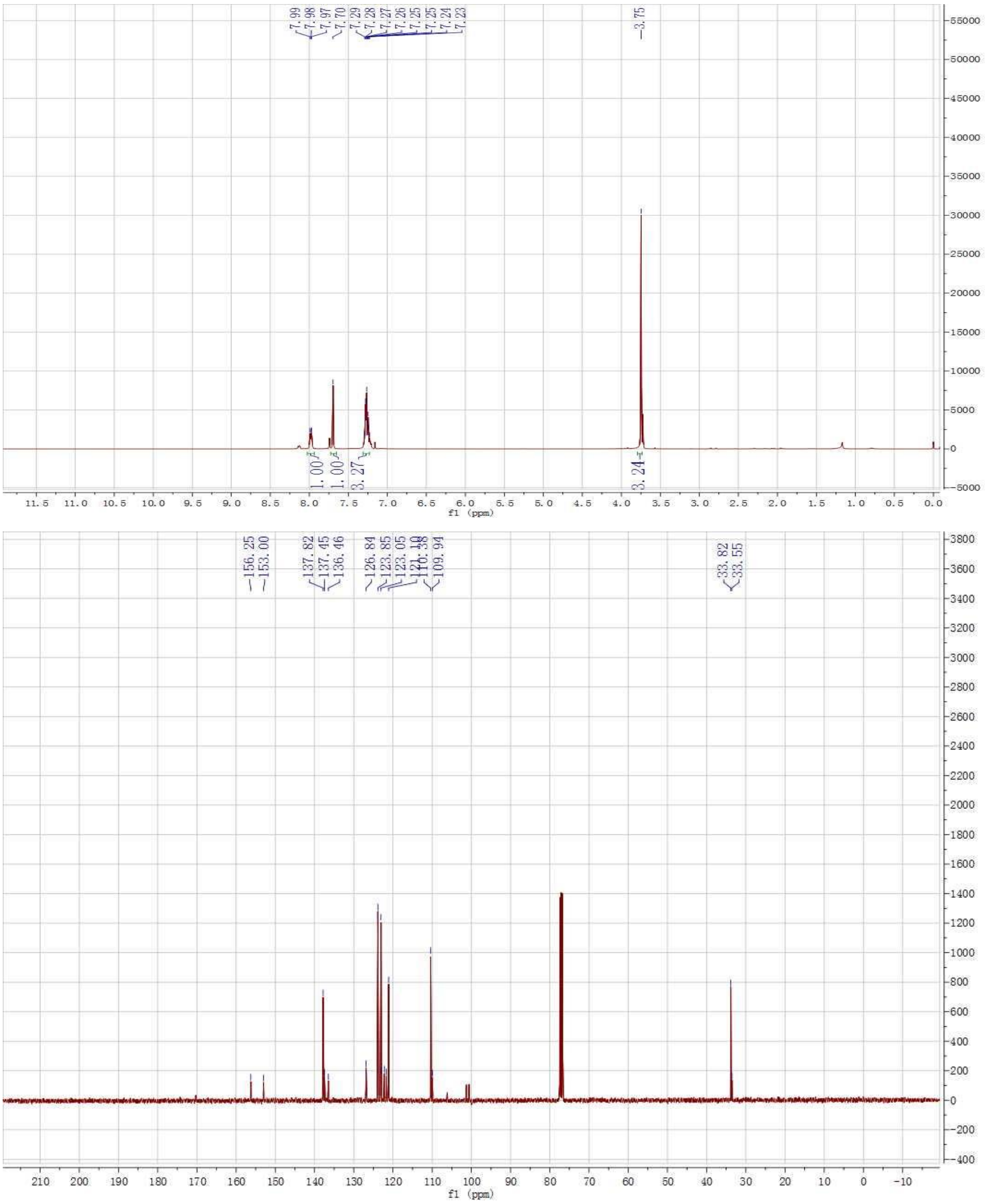

Supplementary Figure 22. ^{1}H and ^{13}C NMR spectrum for **A**

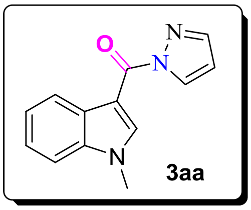

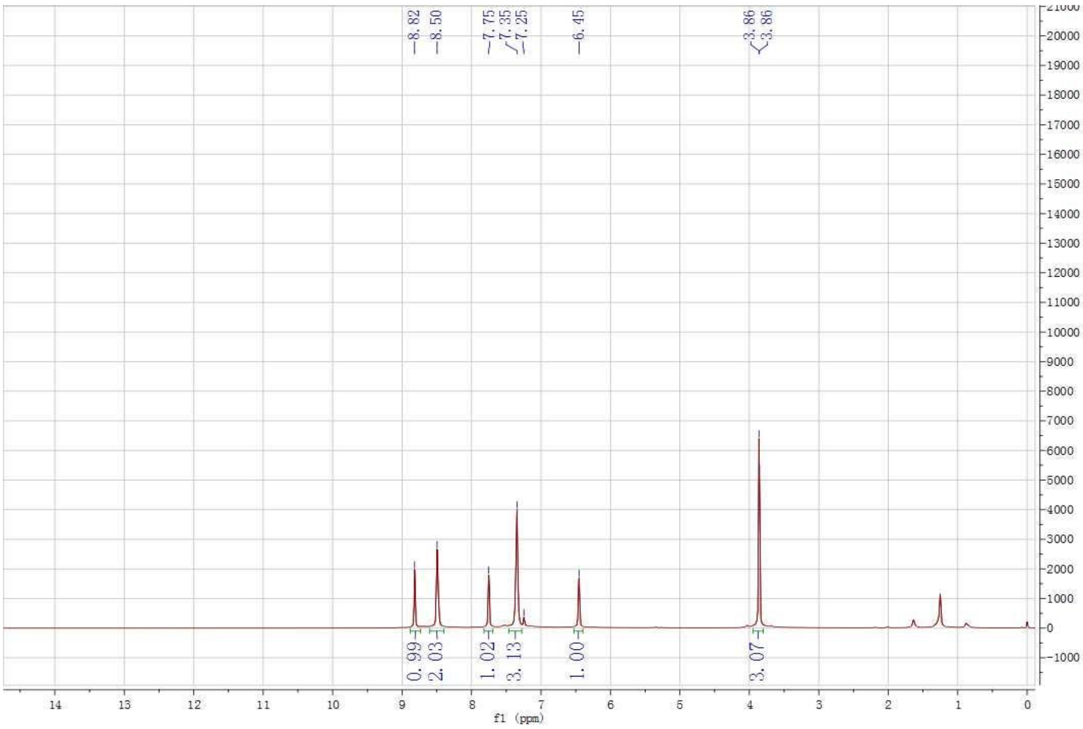

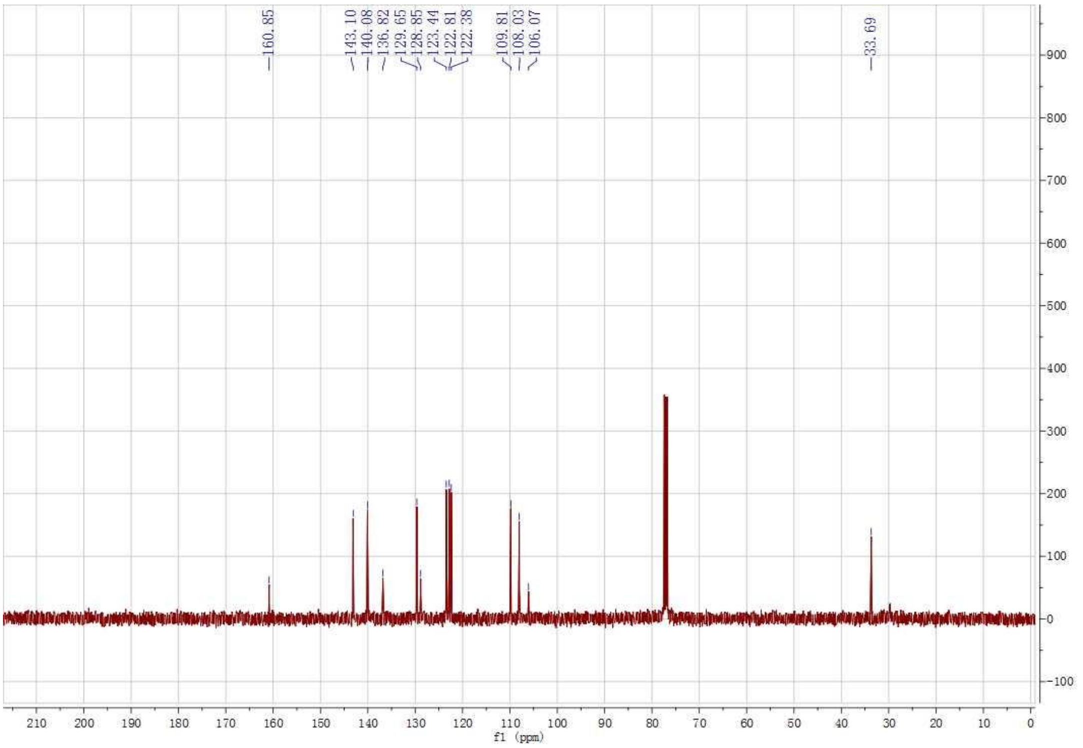

Supplementary Figure 23. ^{1}H and ^{13}C NMR spectrum for **3aa**

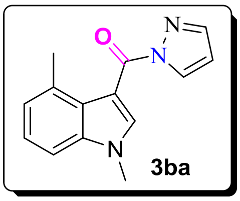

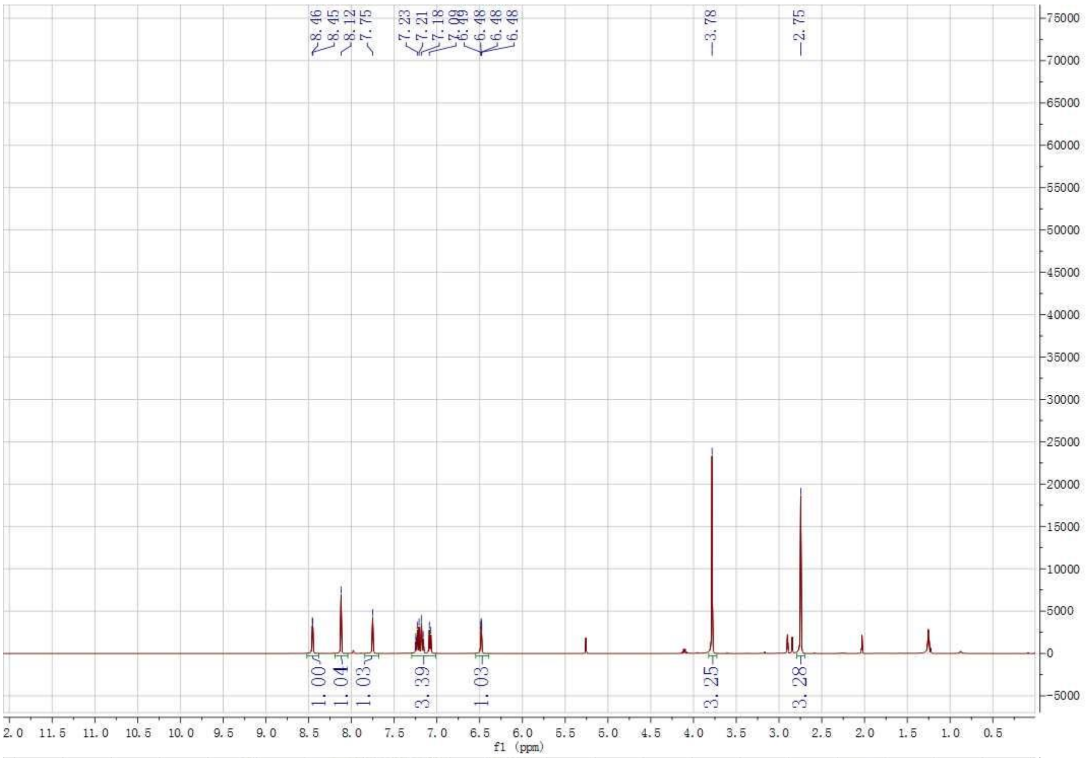

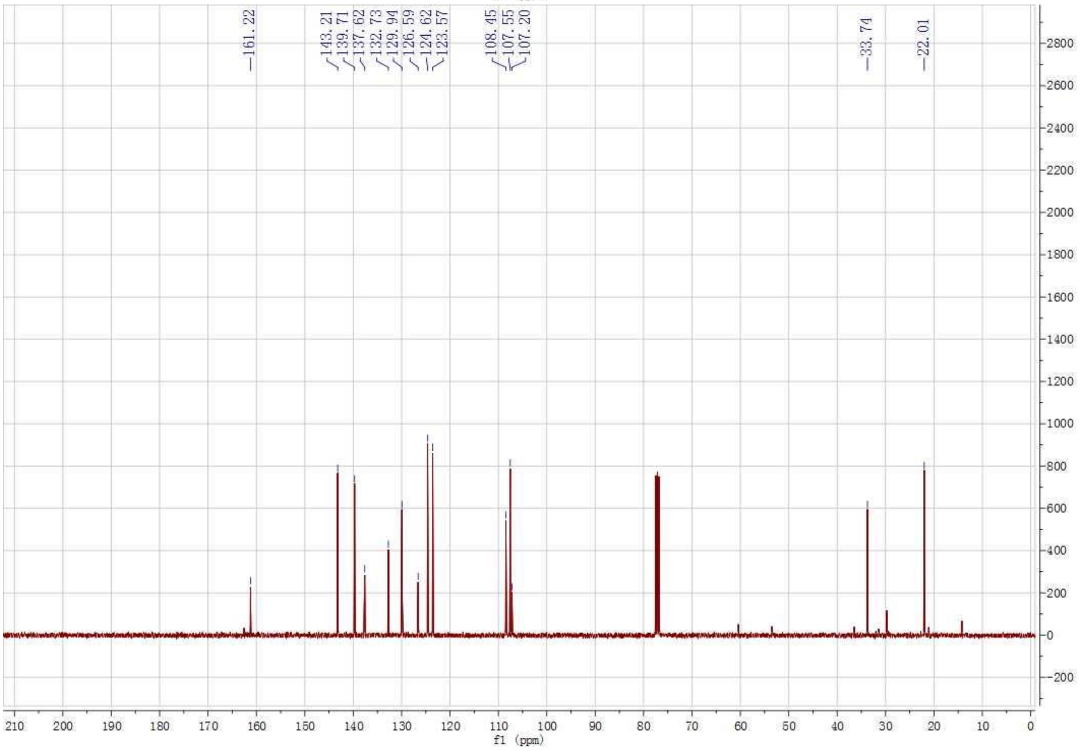

Supplementary Figure 24. ^{1}H and ^{13}C NMR spectrum for **3ba**

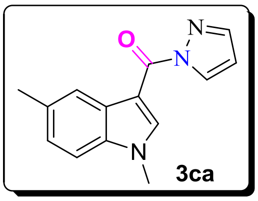

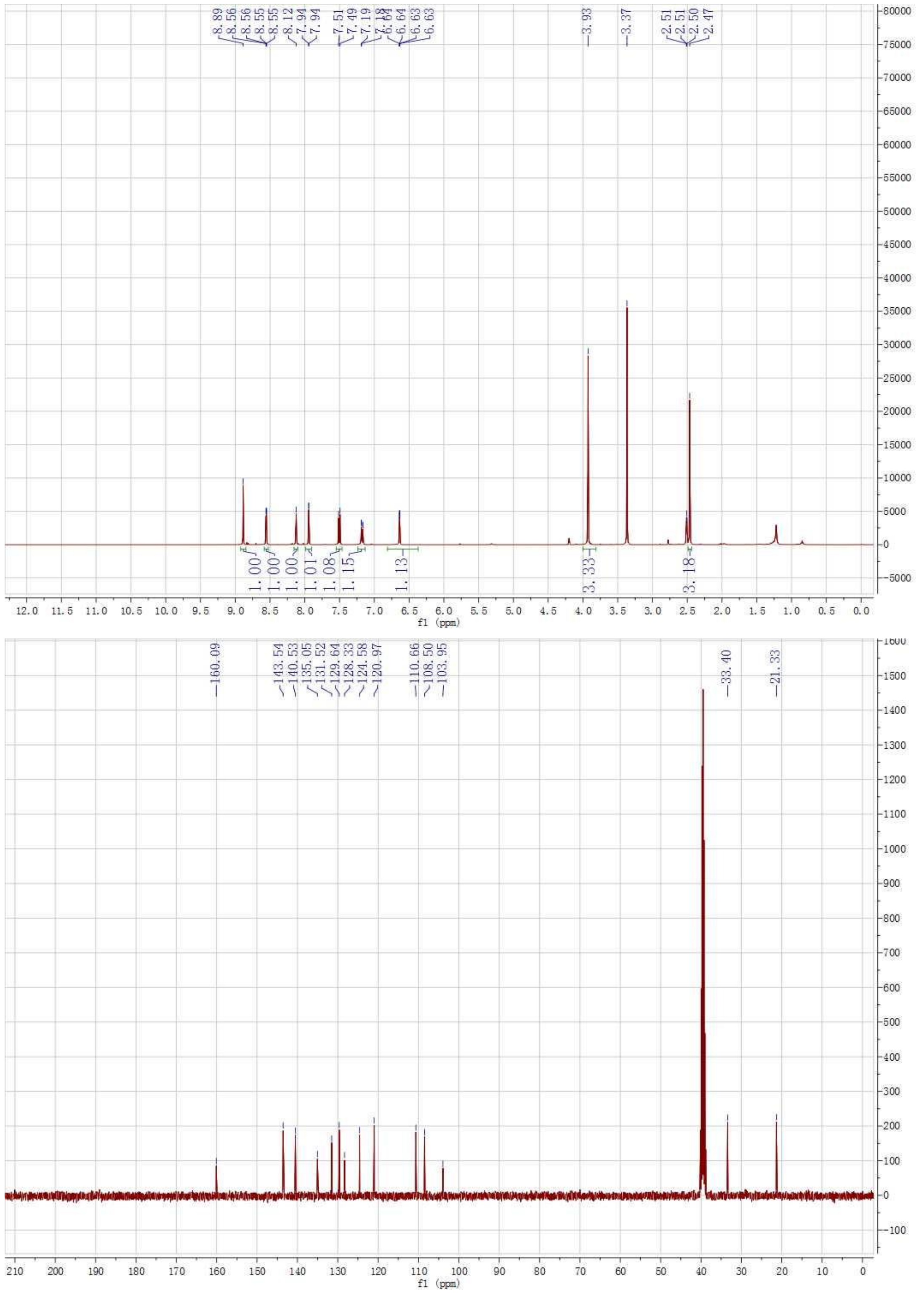

Supplementary Figure 25. ^{1}H and ^{13}C NMR spectrum for **3ca**

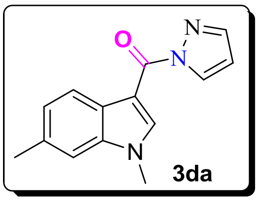

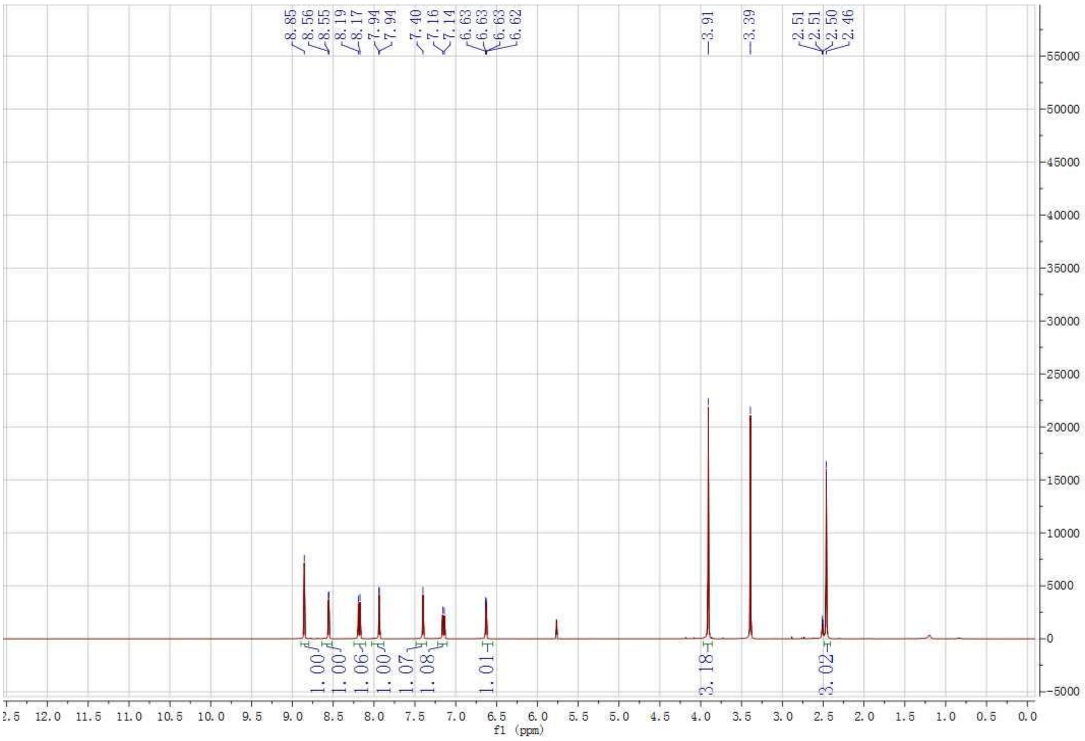

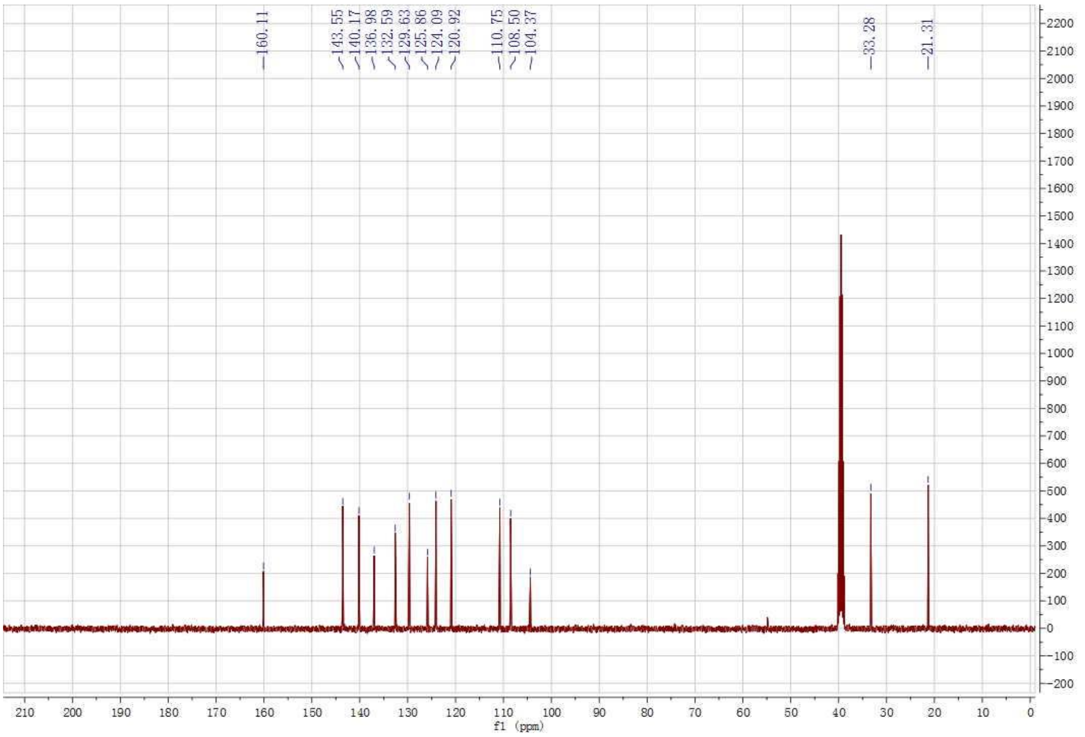

Supplementary Figure 26. ^{1}H and ^{13}C NMR spectrum for **3da**

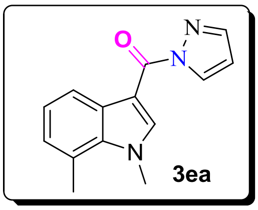

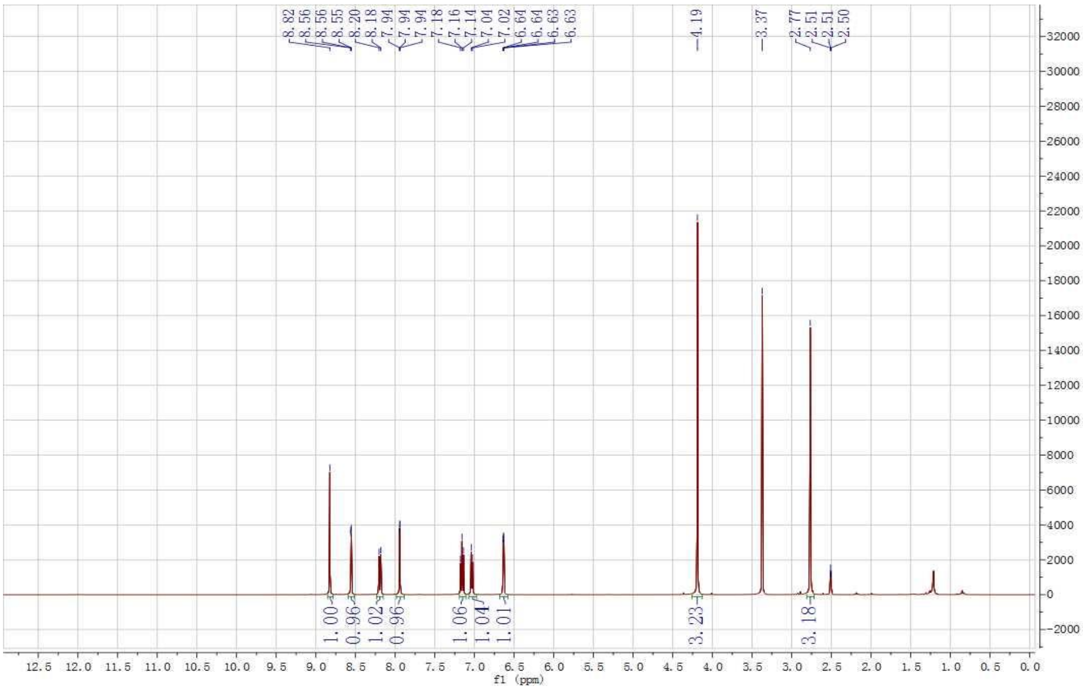

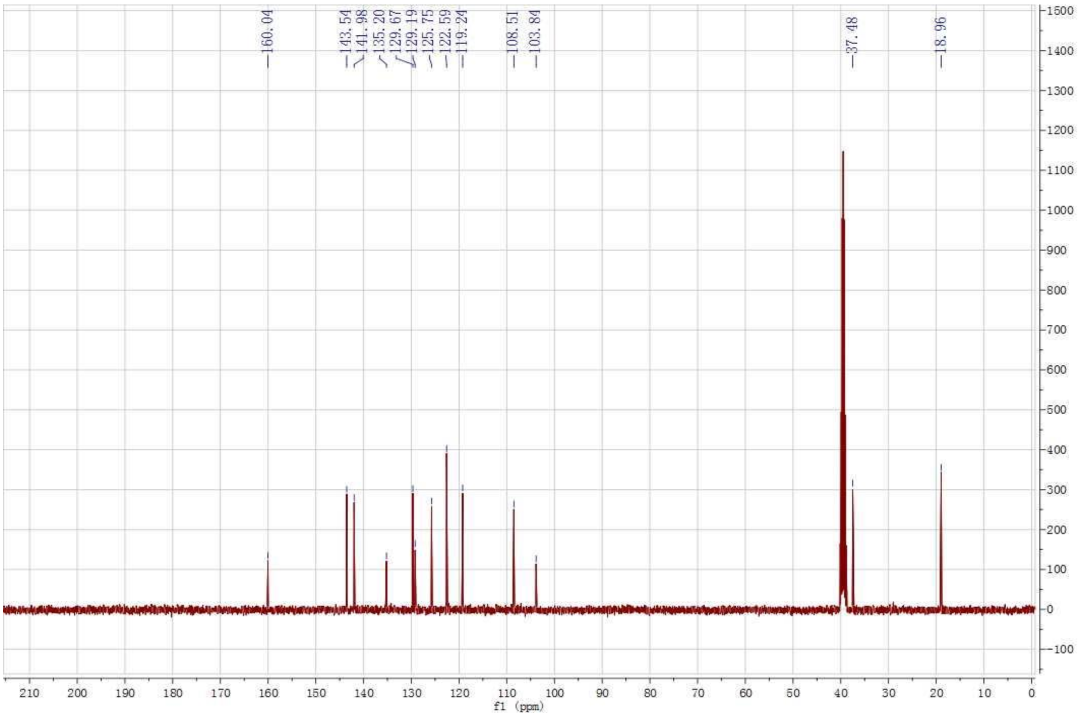

Supplementary Figure 27. ^{1}H and ^{13}C NMR spectrum for **3ea**

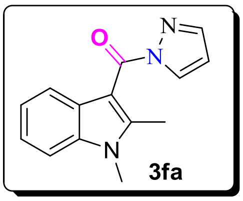

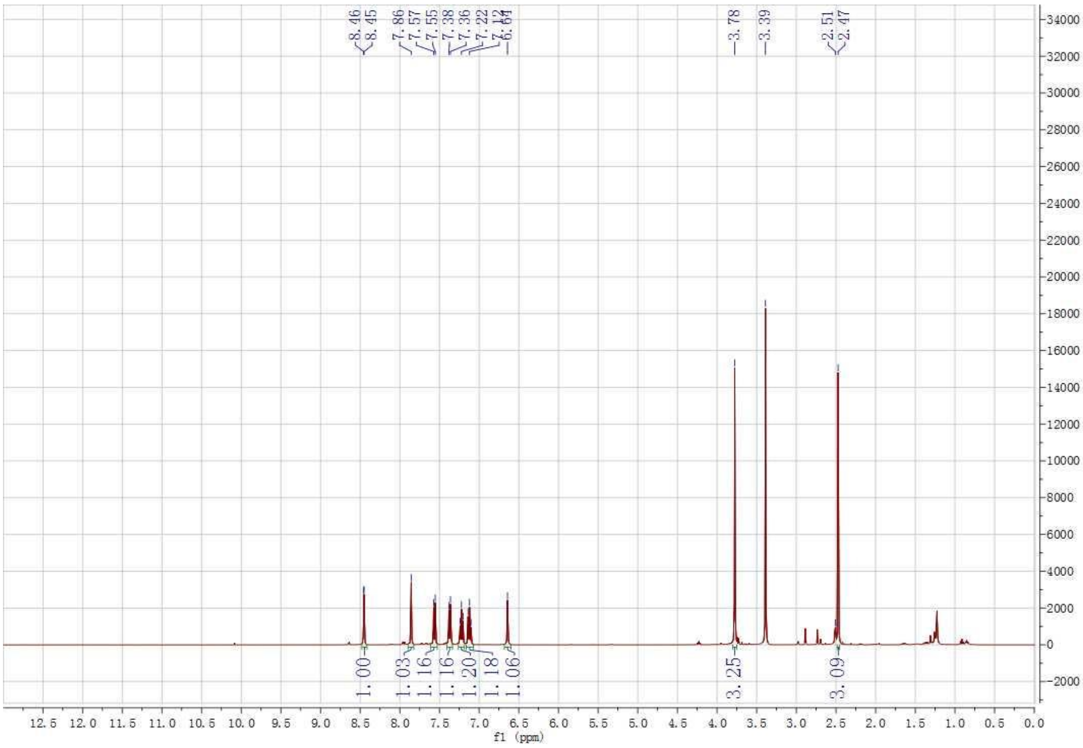

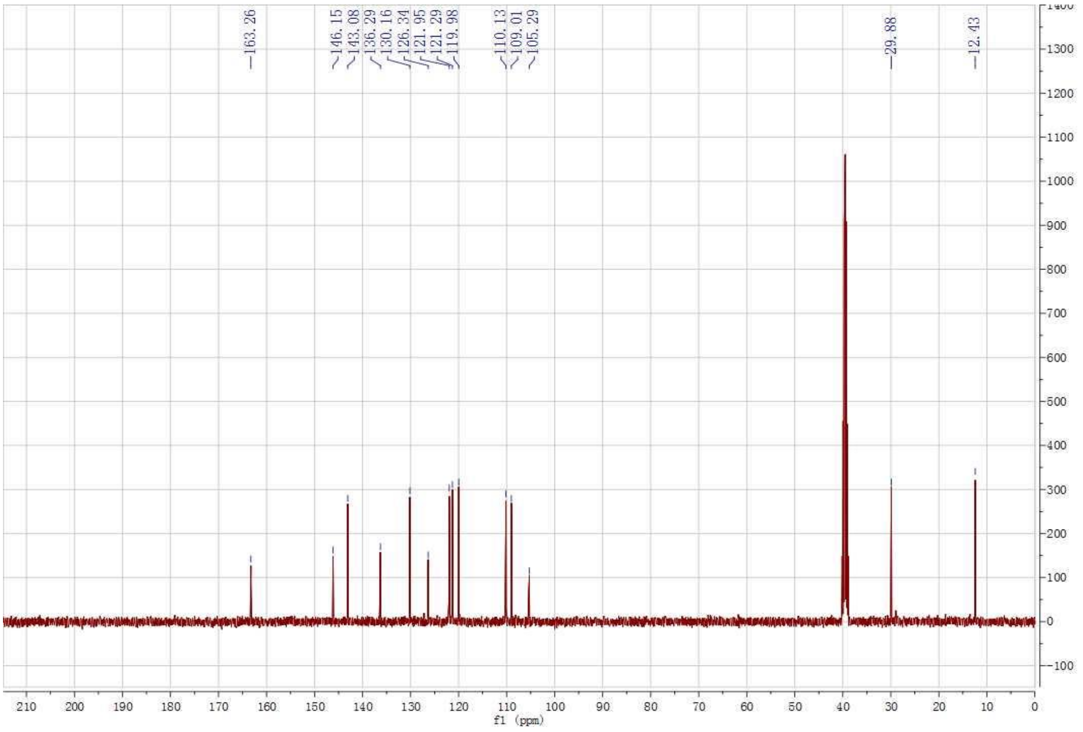

Supplementary Figure 28. ^{1}H and ^{13}C NMR spectrum for **3fa**

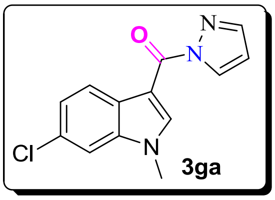

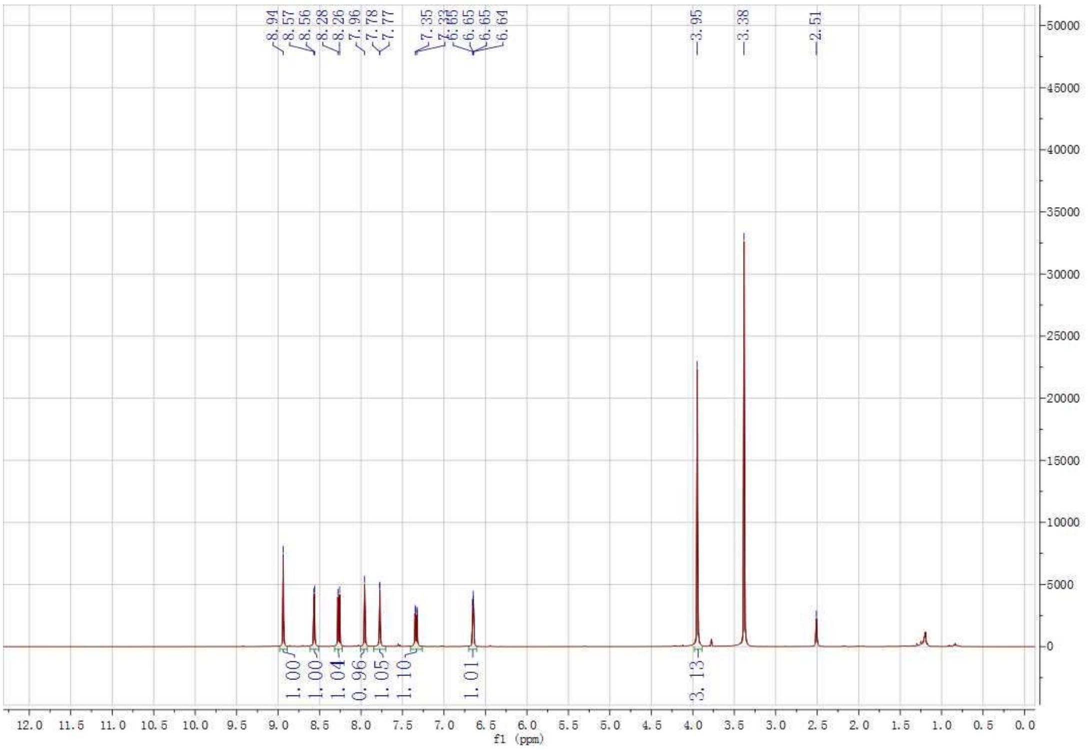

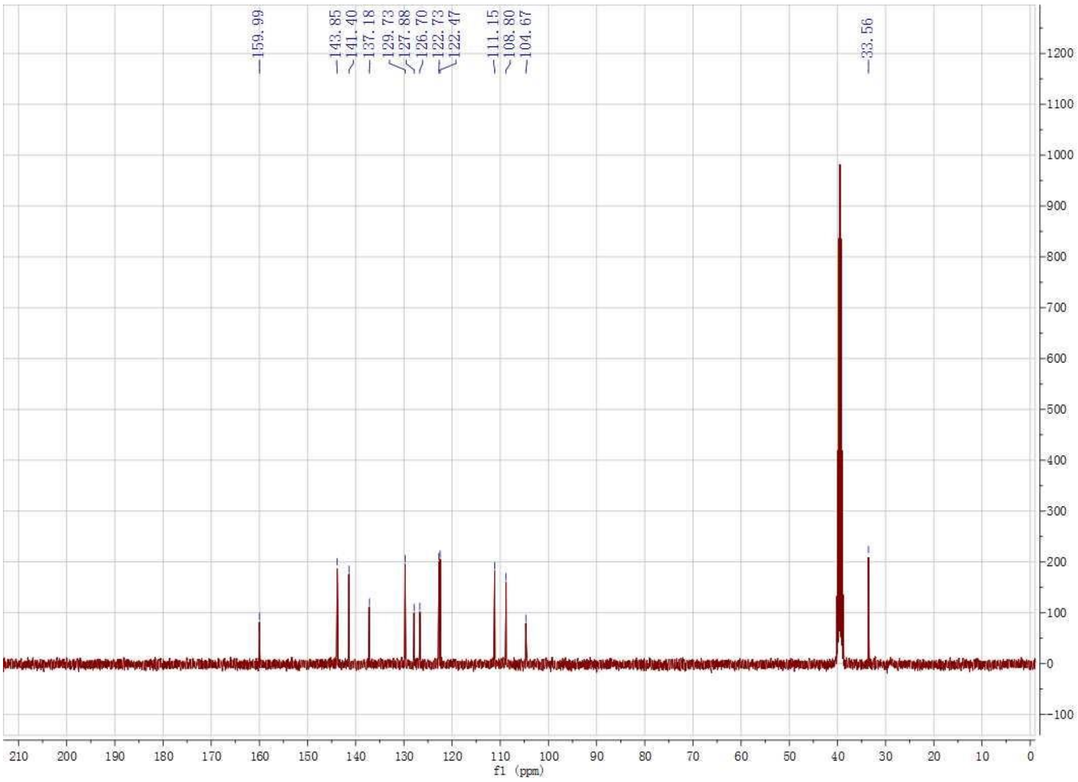

Supplementary Figure 29. ^{1}H and ^{13}C NMR spectrum for **3ga**

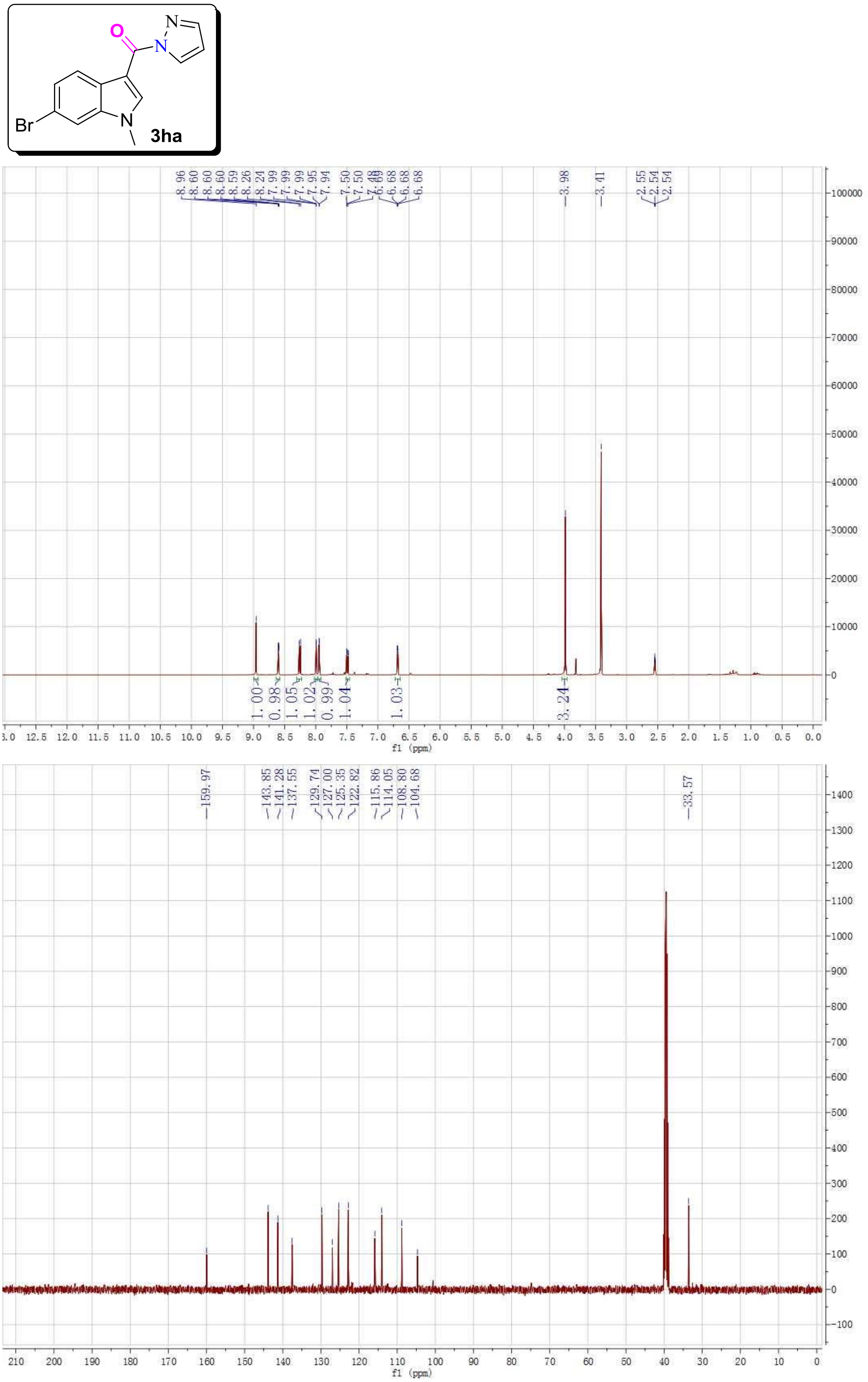

Supplementary Figure 30. ^{1}H and ^{13}C NMR spectrum for **3ha**

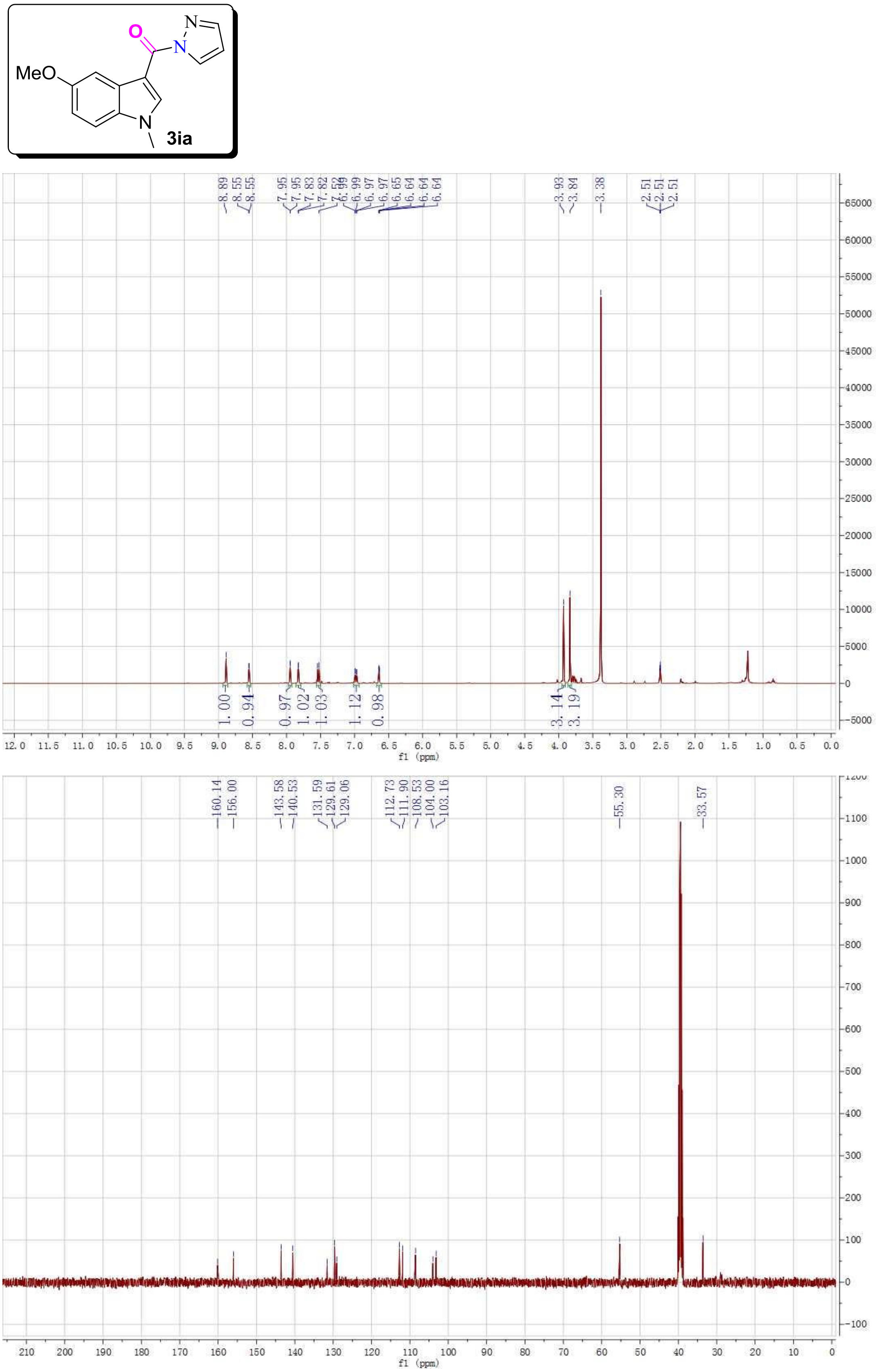

Supplementary Figure 31. ^{1}H and ^{13}C NMR spectrum for **3ia**

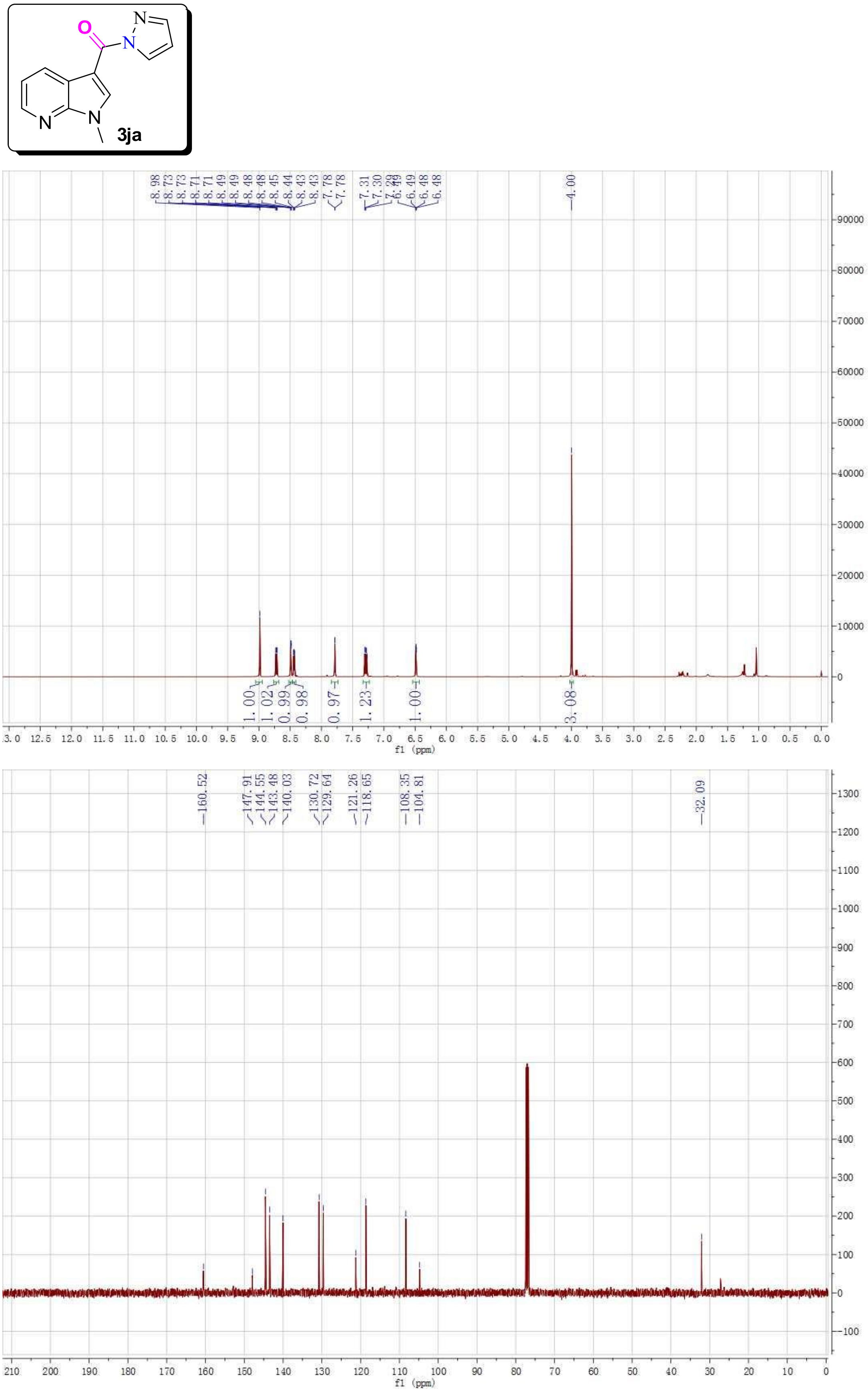

Supplementary Figure 32. ^{1}H and ^{13}C NMR spectrum for **3ja**

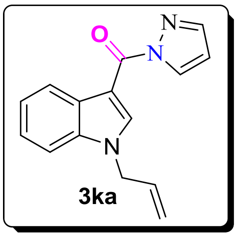

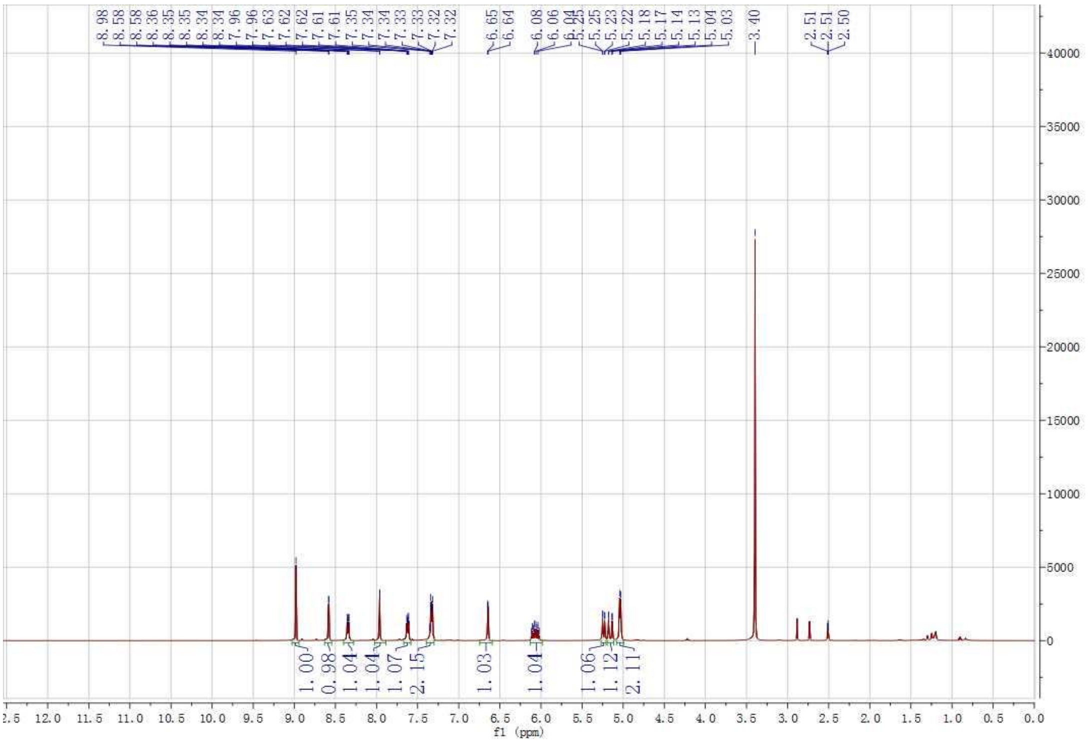

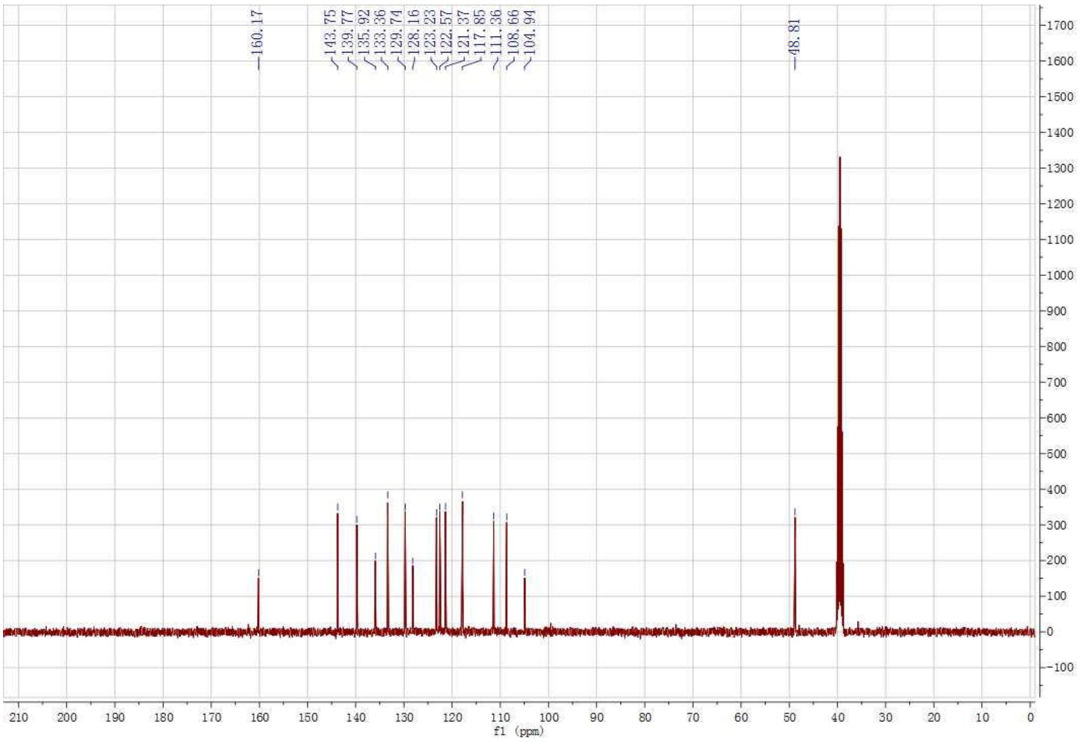

Supplementary Figure 33. ^{1}H and ^{13}C NMR spectrum for **3ka**

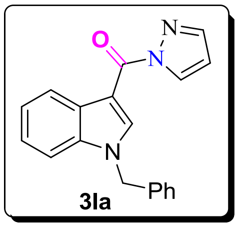

3la

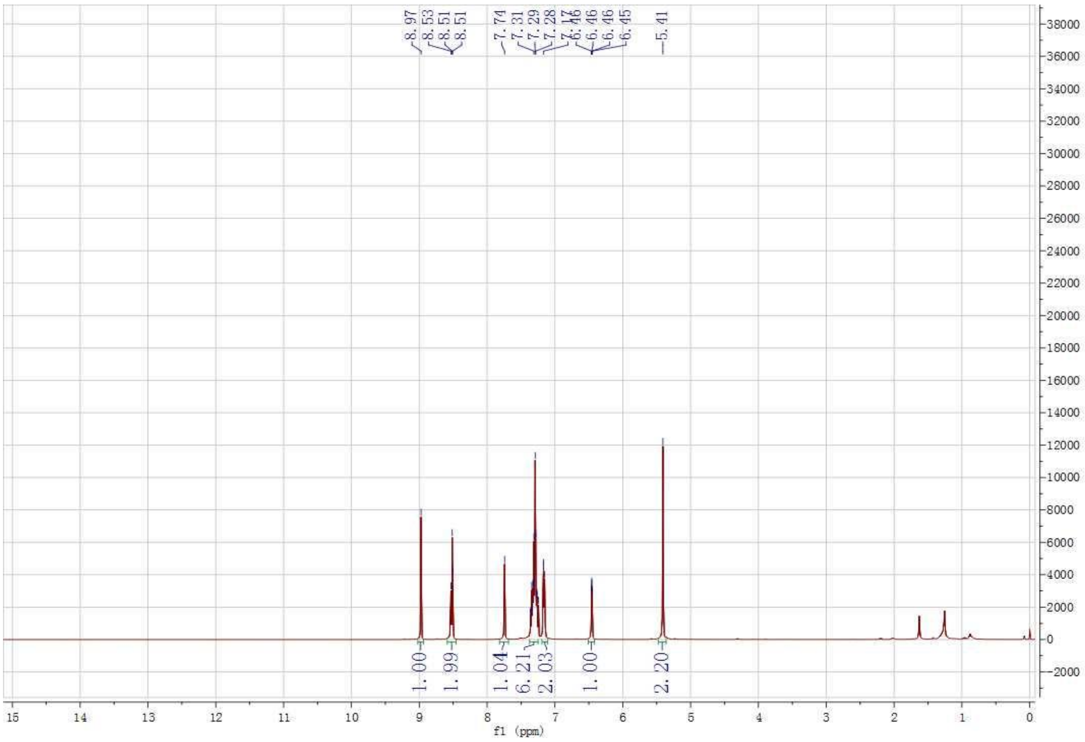

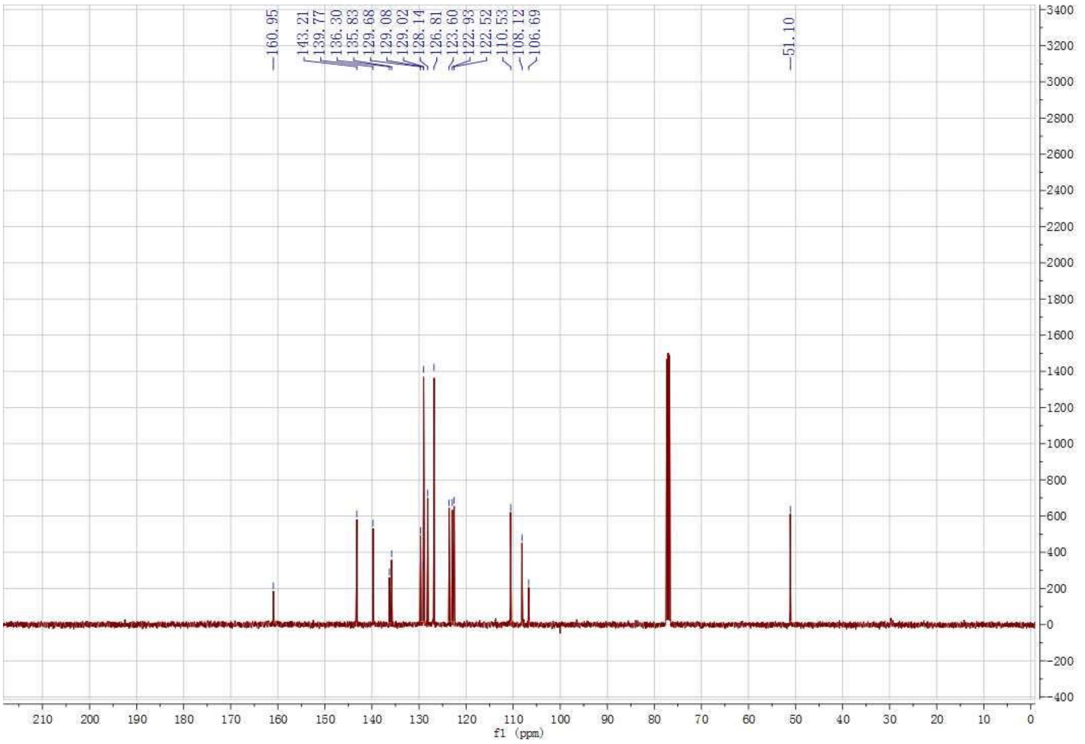

Supplementary Figure 34. ^{1}H and ^{13}C NMR spectrum for **3la**

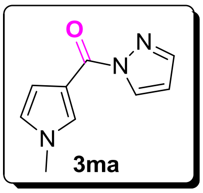

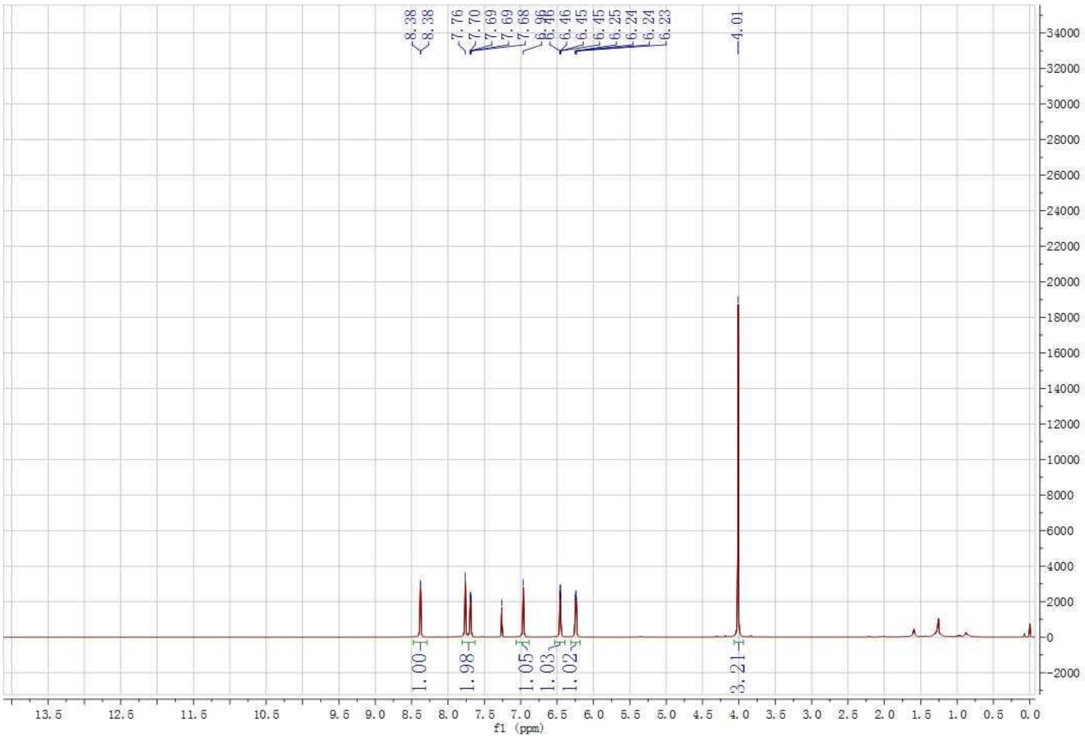

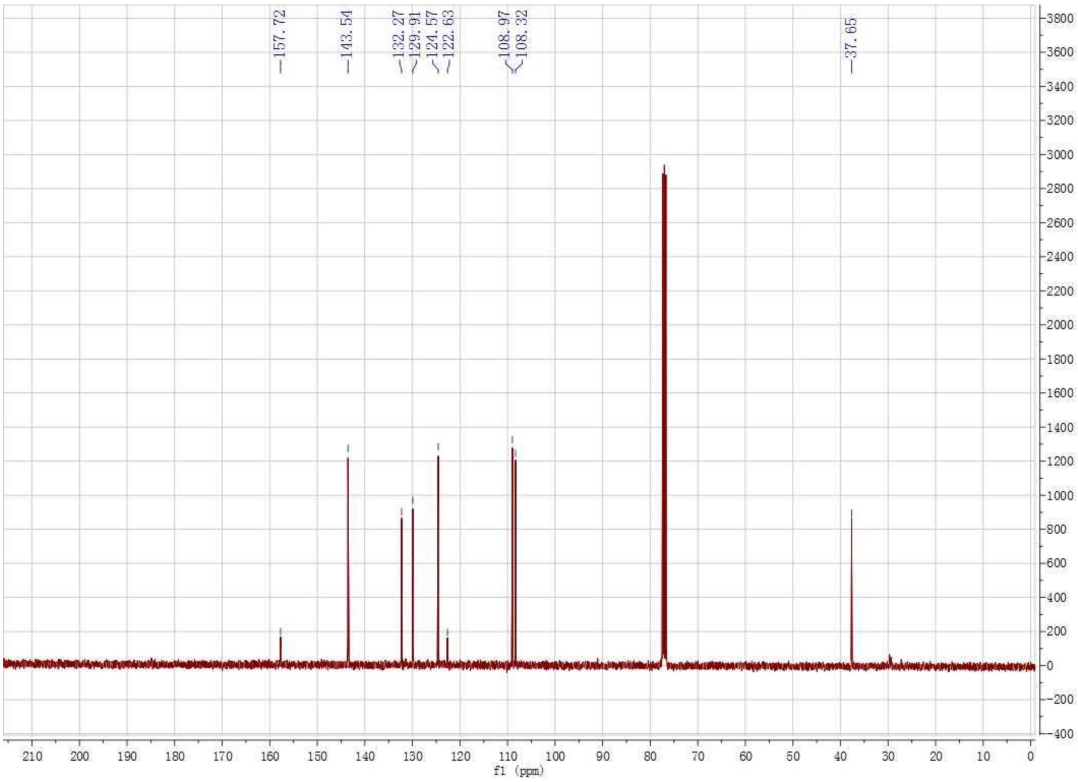

Supplementary Figure 35. ^{1}H and ^{13}C NMR spectrum for **3ma**

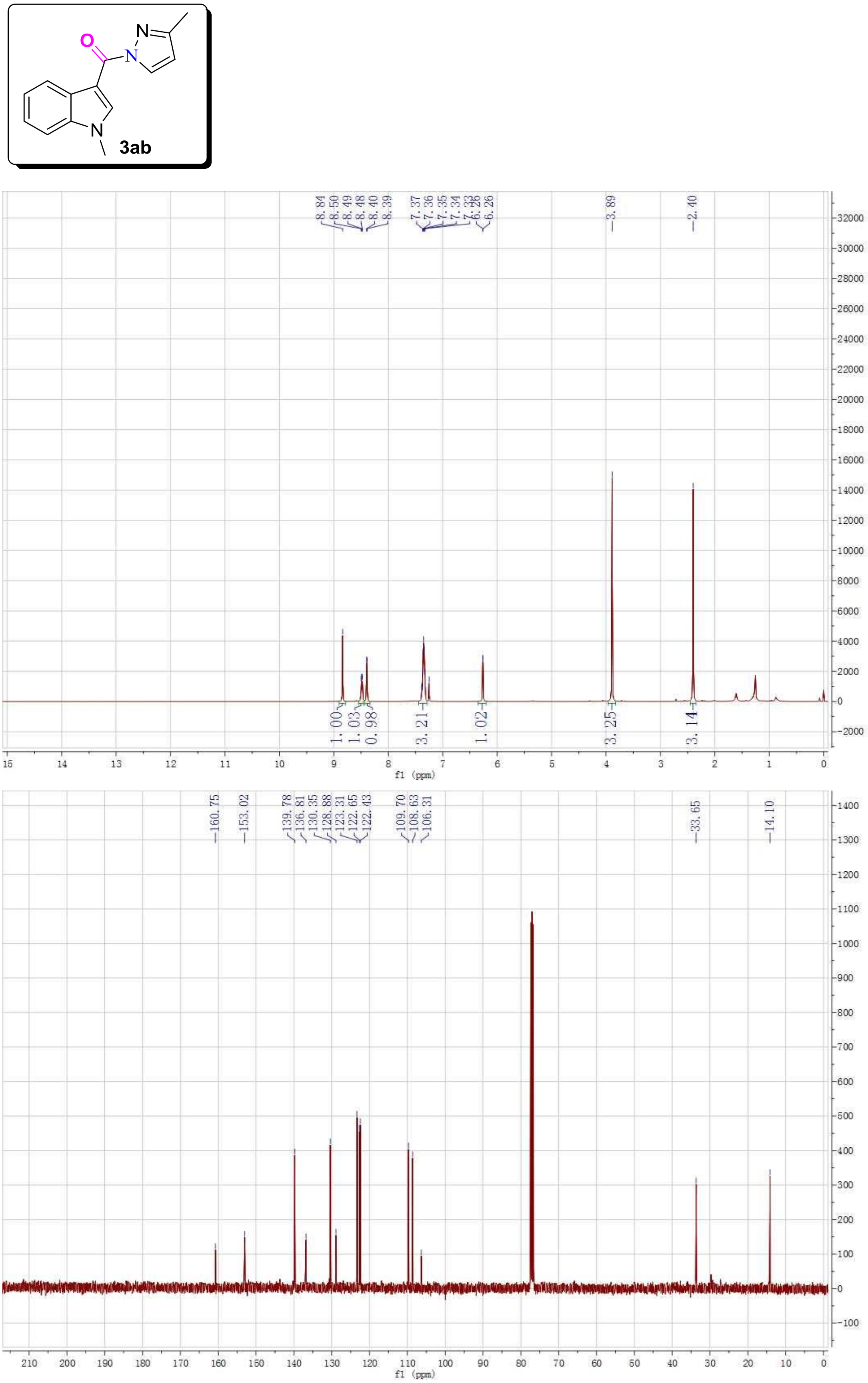

Supplementary Figure 36. ^{1}H and ^{13}C NMR spectrum for **3ab**

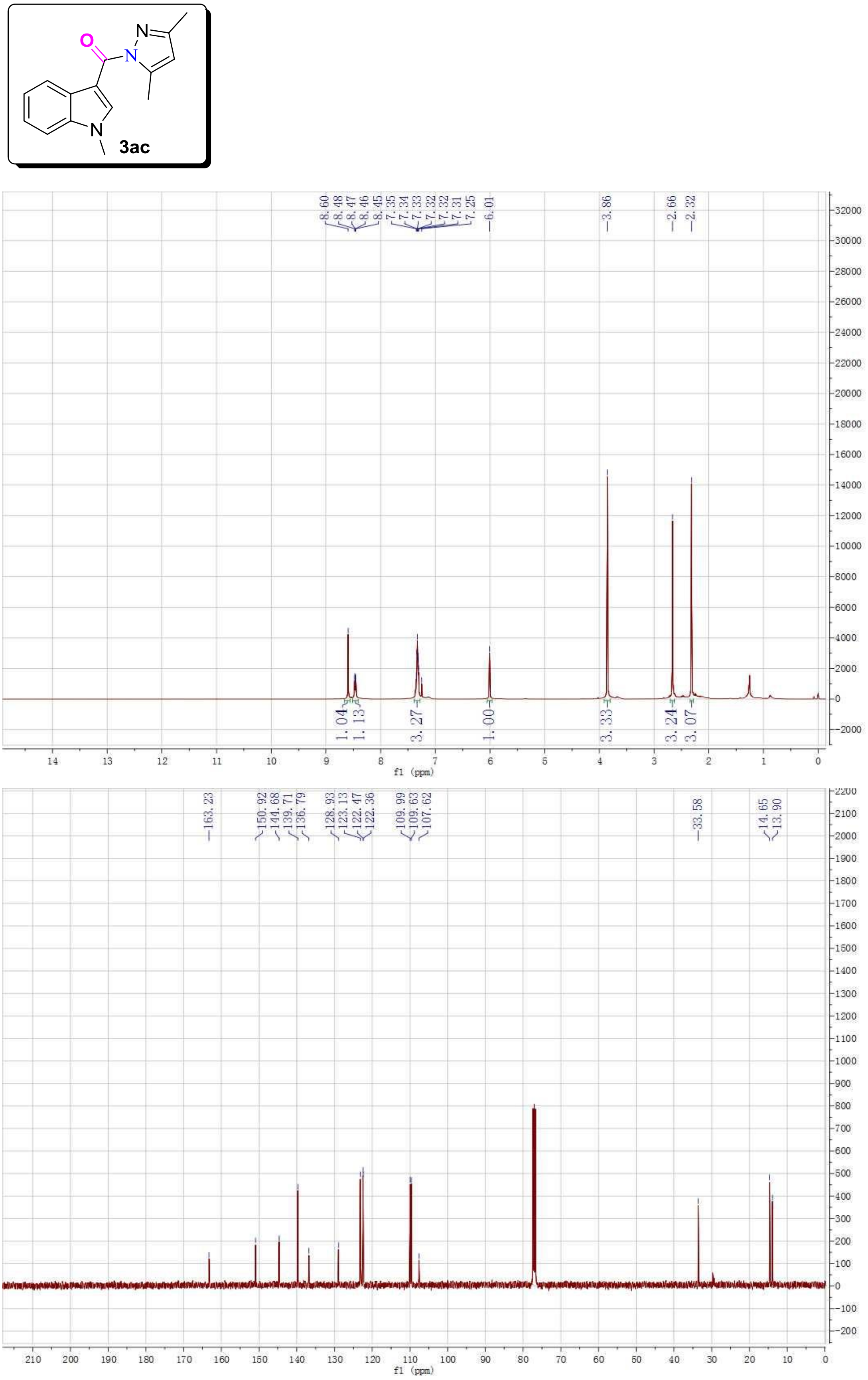

Supplementary Figure 37. ^{1}H and ^{13}C NMR spectrum for **3ac**

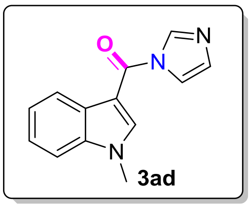

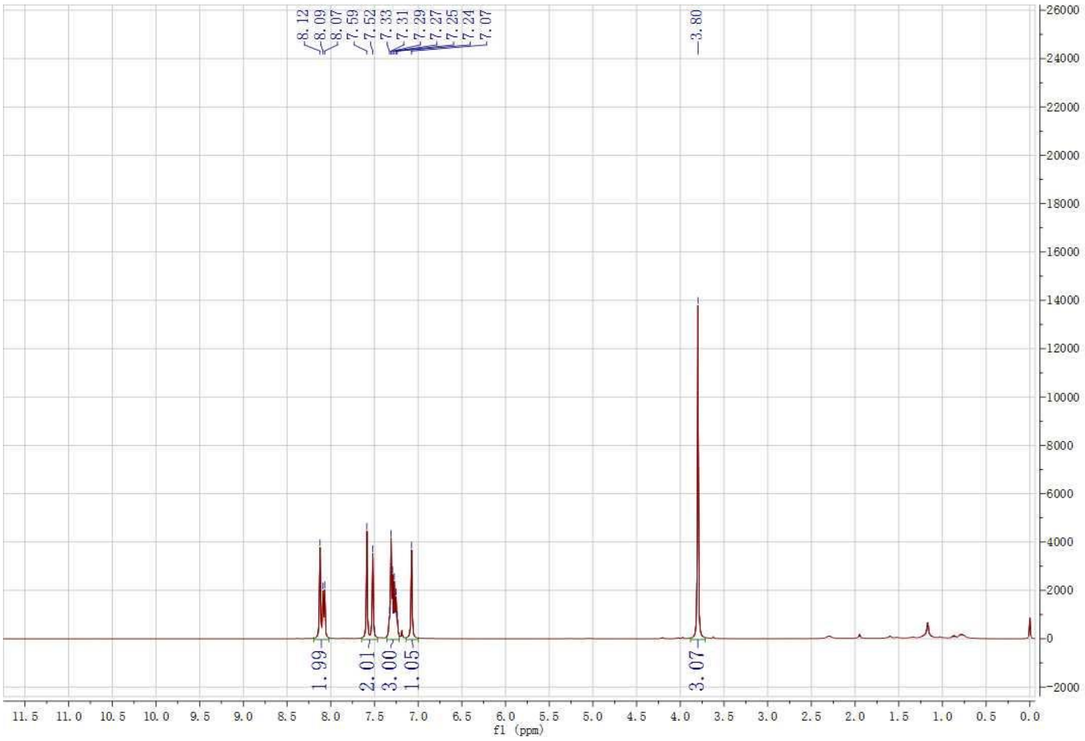

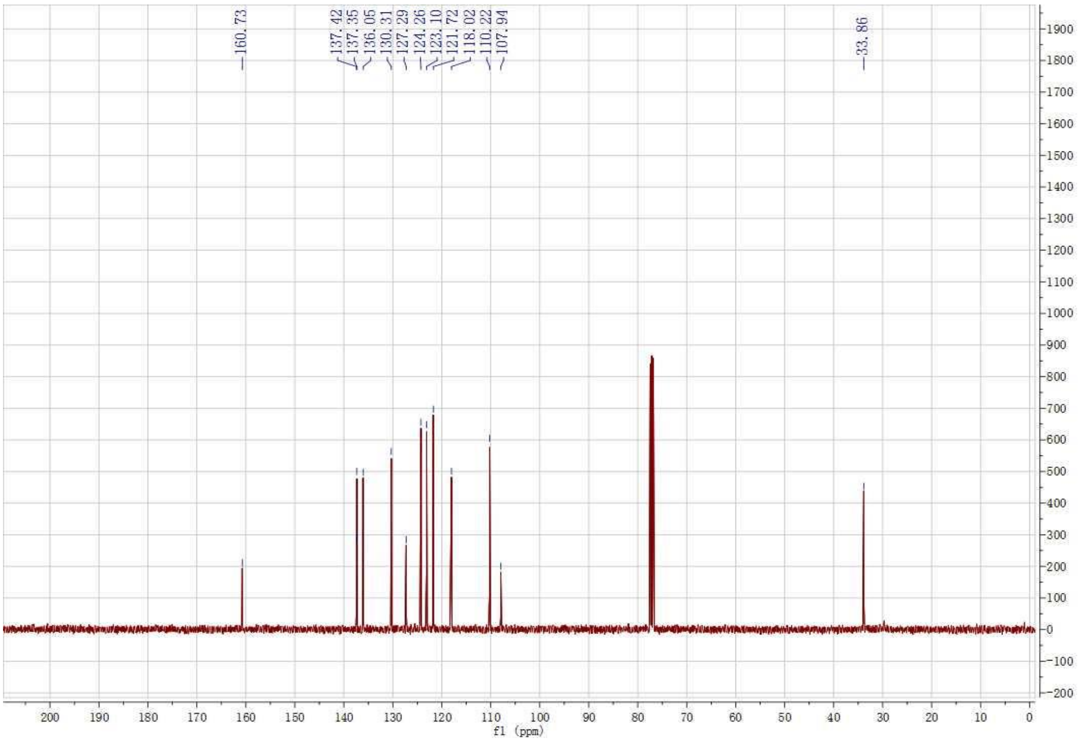

Supplementary Figure 38. ^{1}H and ^{13}C NMR spectrum for **3ad**

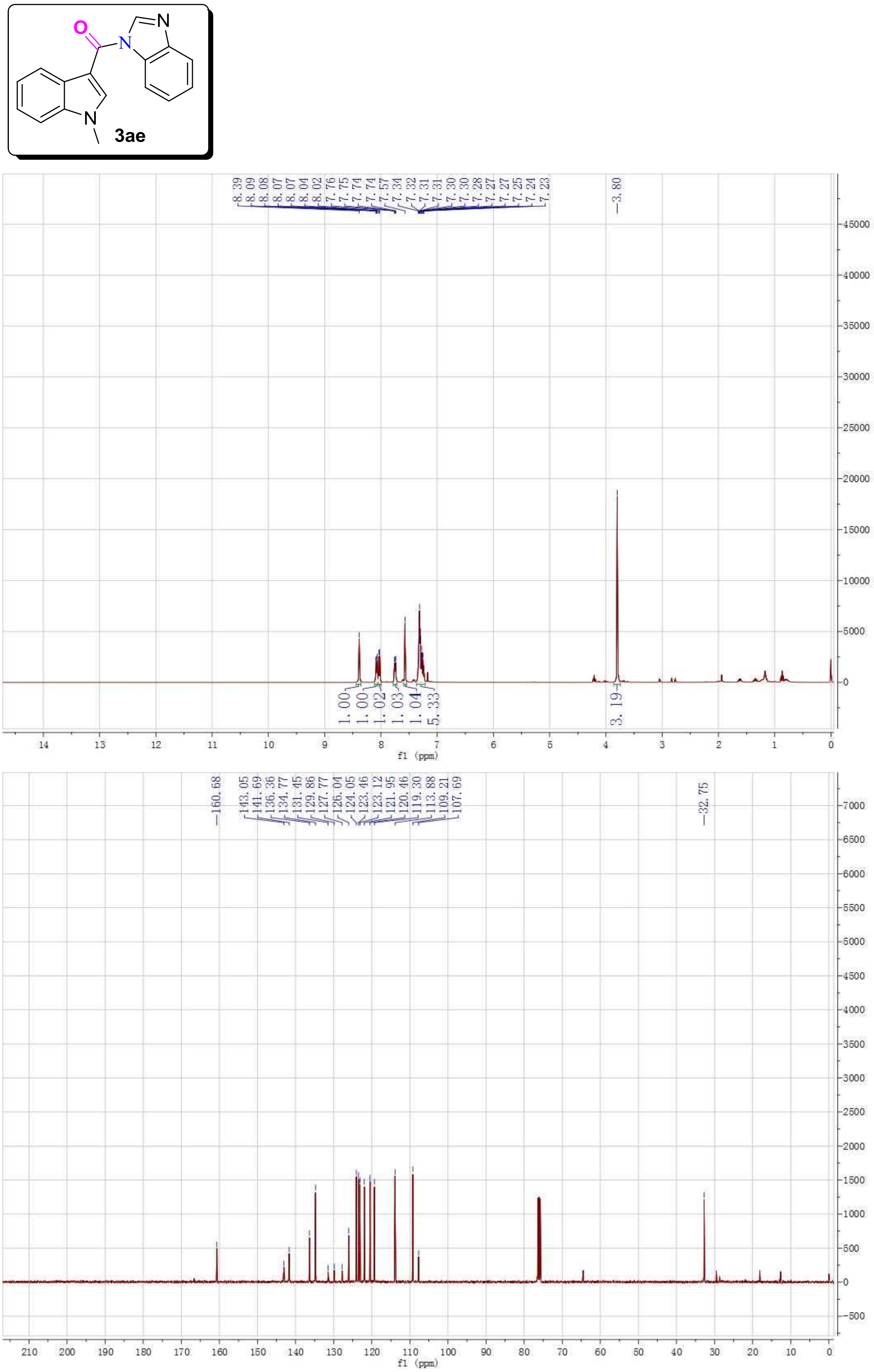

Supplementary Figure 39. ^{1}H and ^{13}C NMR spectrum for **3ae**

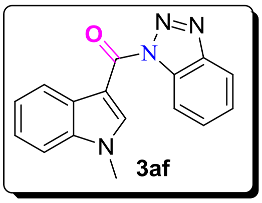

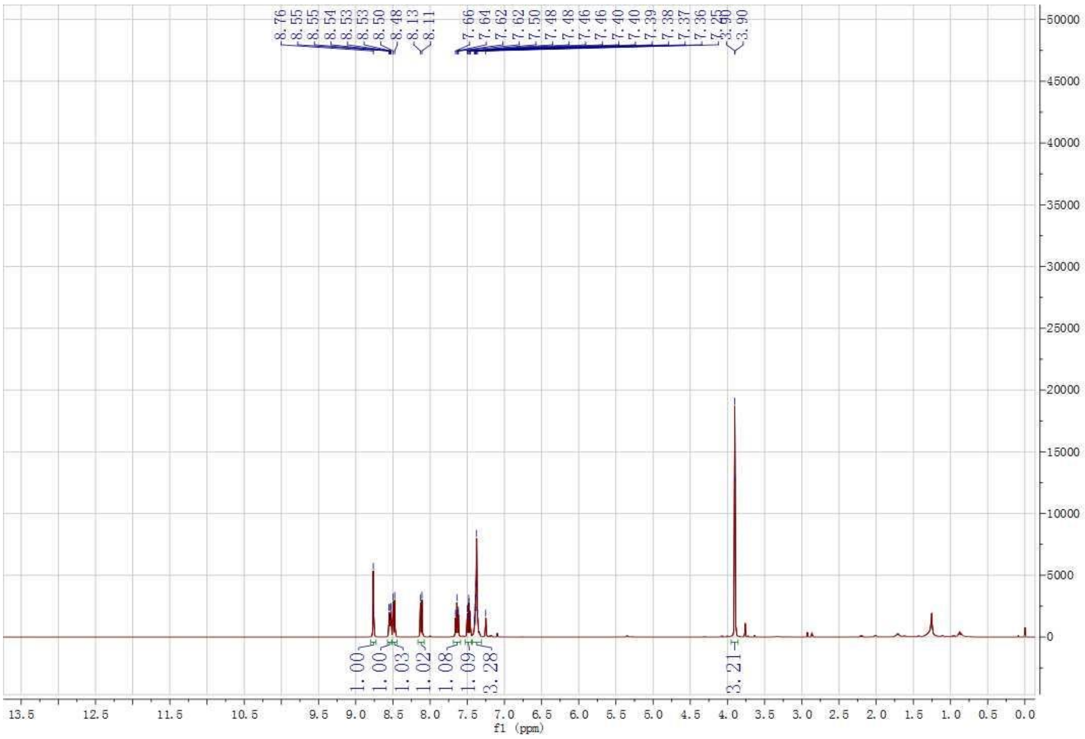

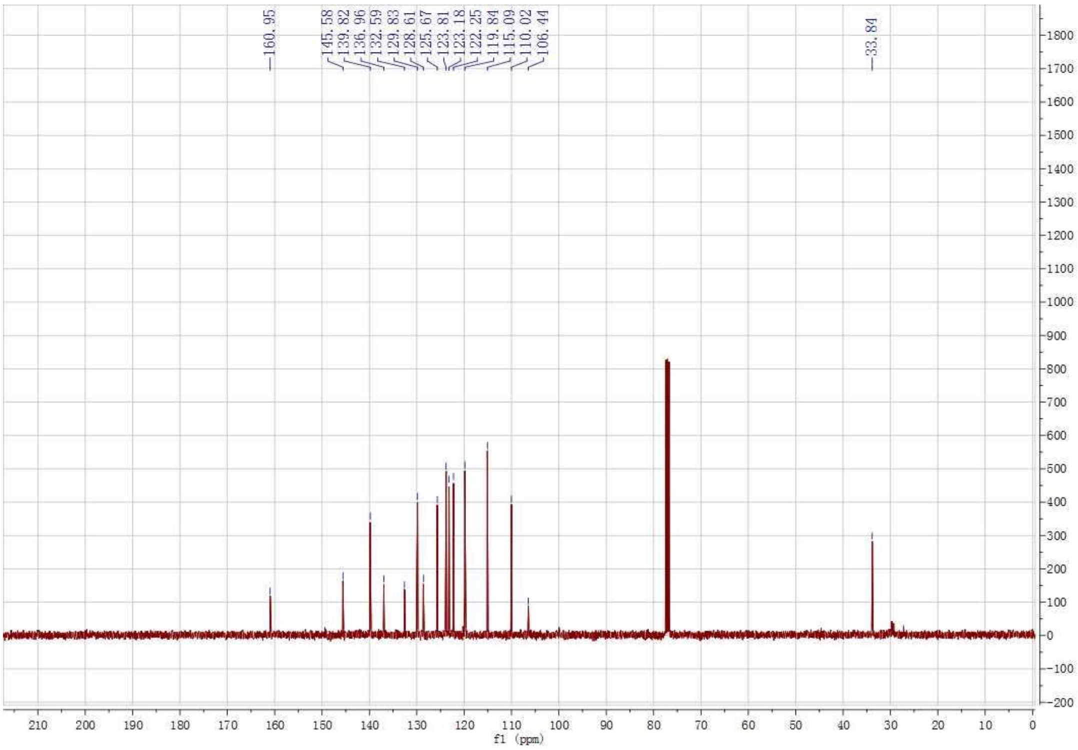

Supplementary Figure 40. ^{1}H and ^{13}C NMR spectrum for **3af**

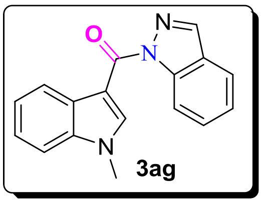

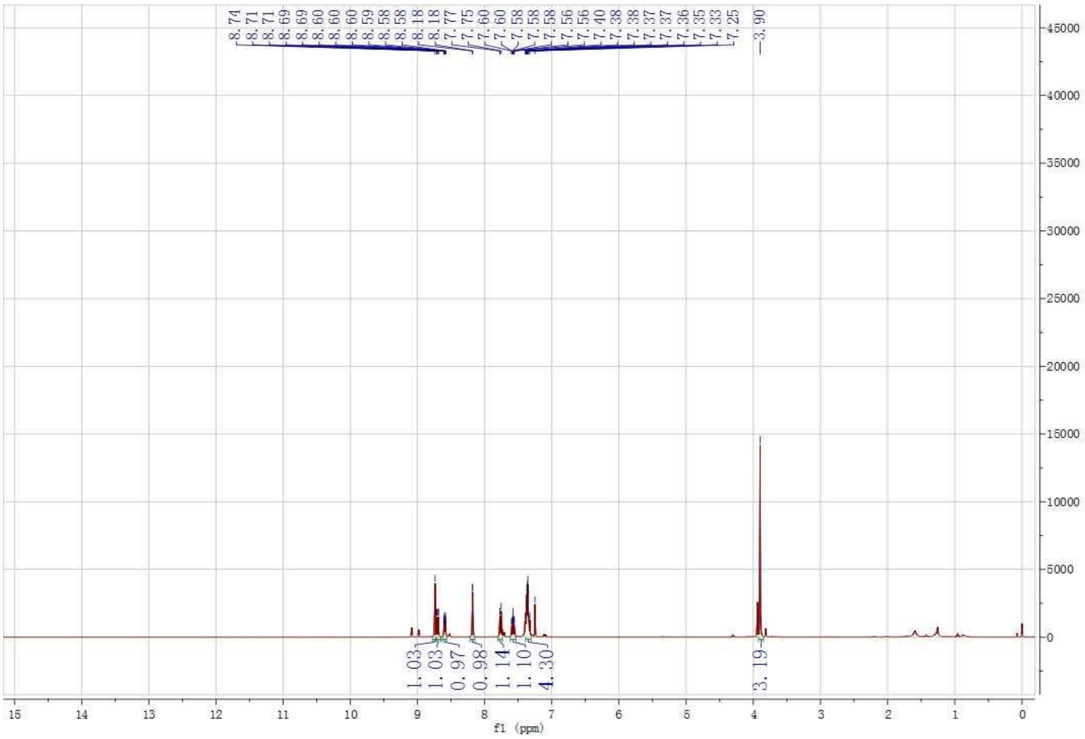

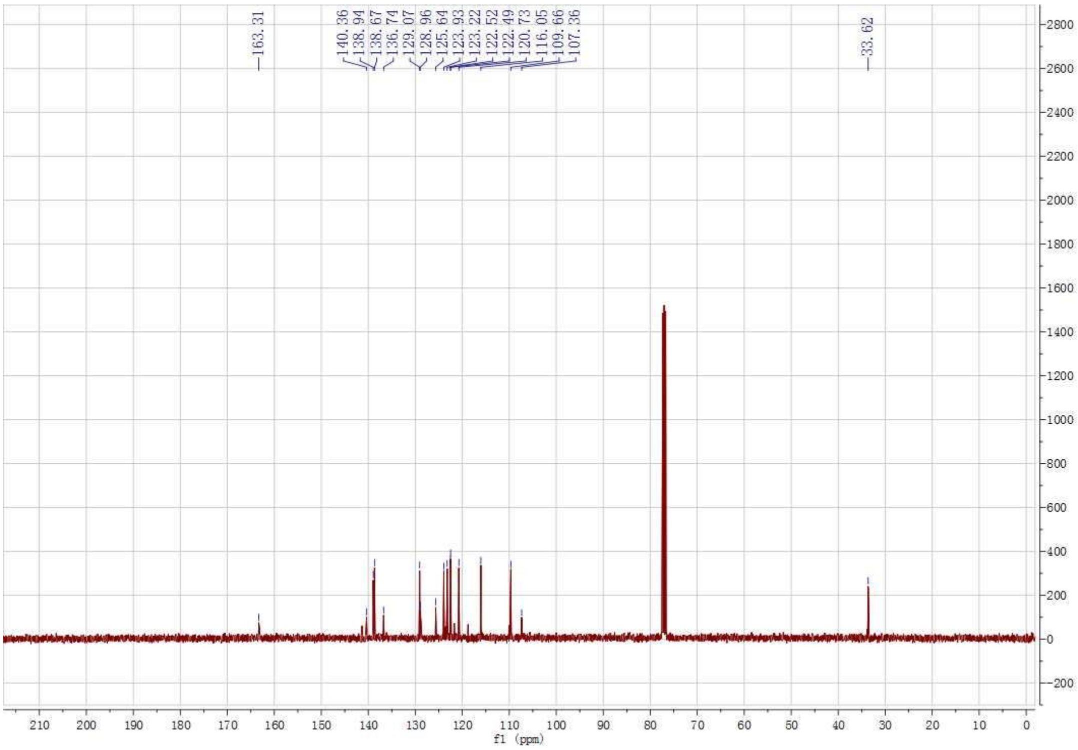

Supplementary Figure 41. ^{1}H and ^{13}C NMR spectrum for **3ag**

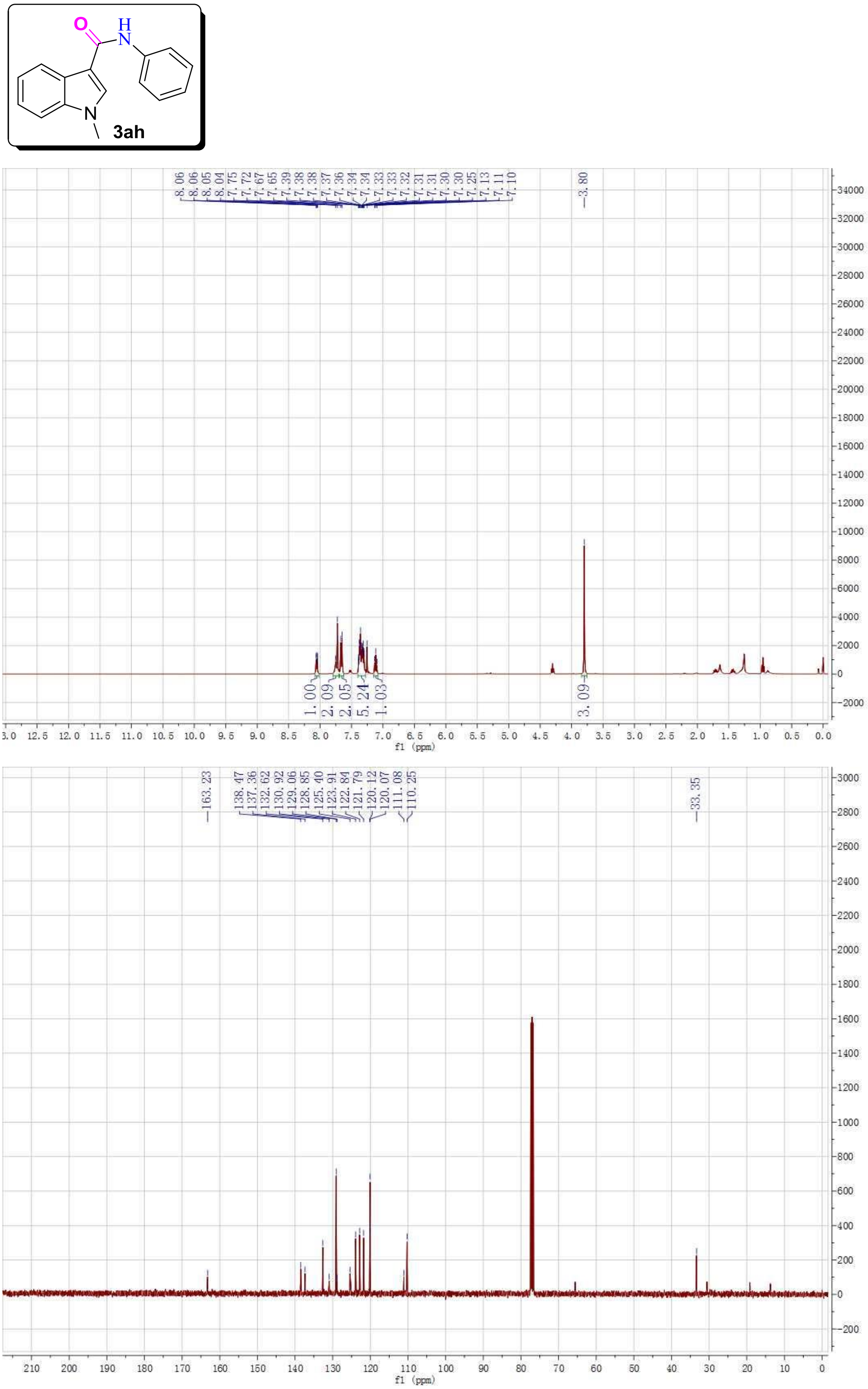

Supplementary Figure 42. ^{1}H and ^{13}C NMR spectrum for **3ah**

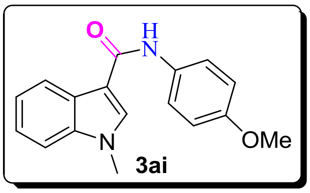

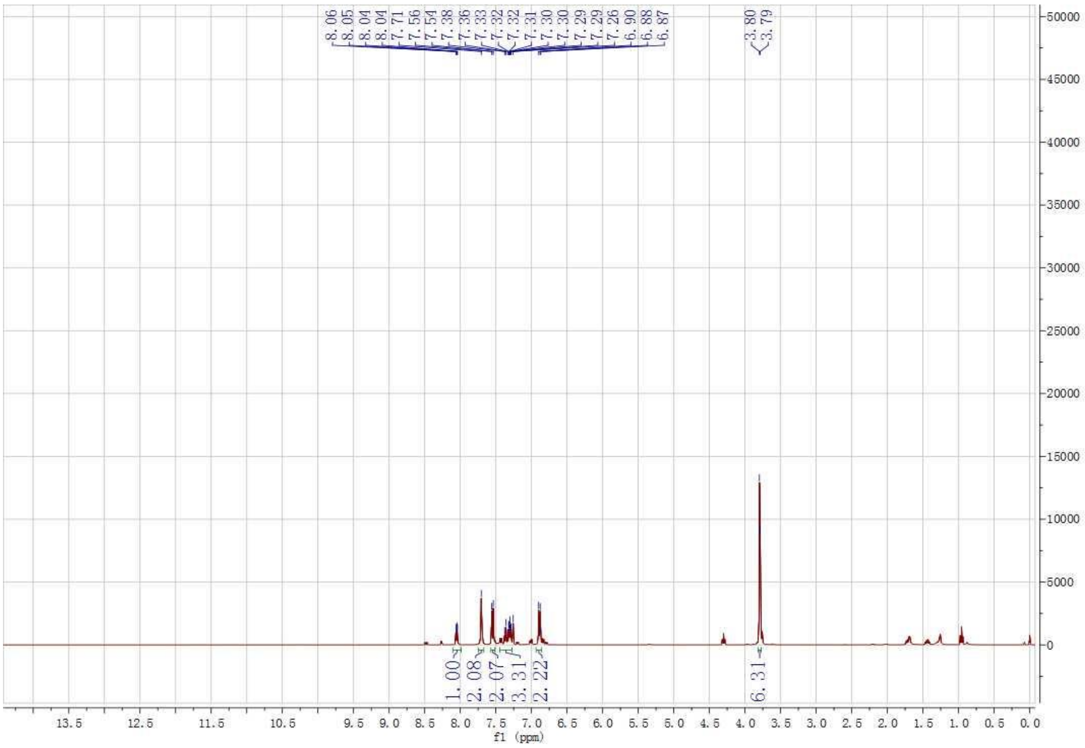

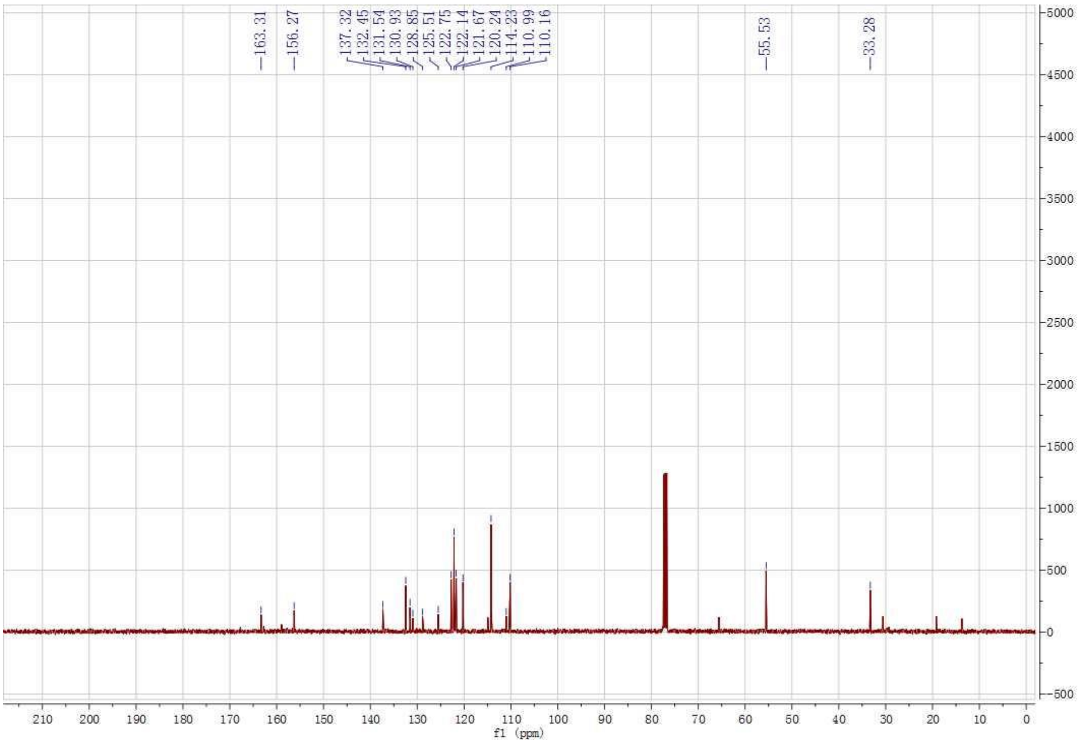

Supplementary Figure 43. ^{1}H and ^{13}C NMR spectrum for **3ai**

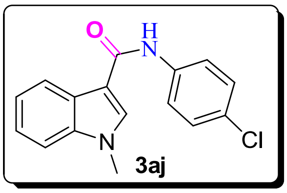

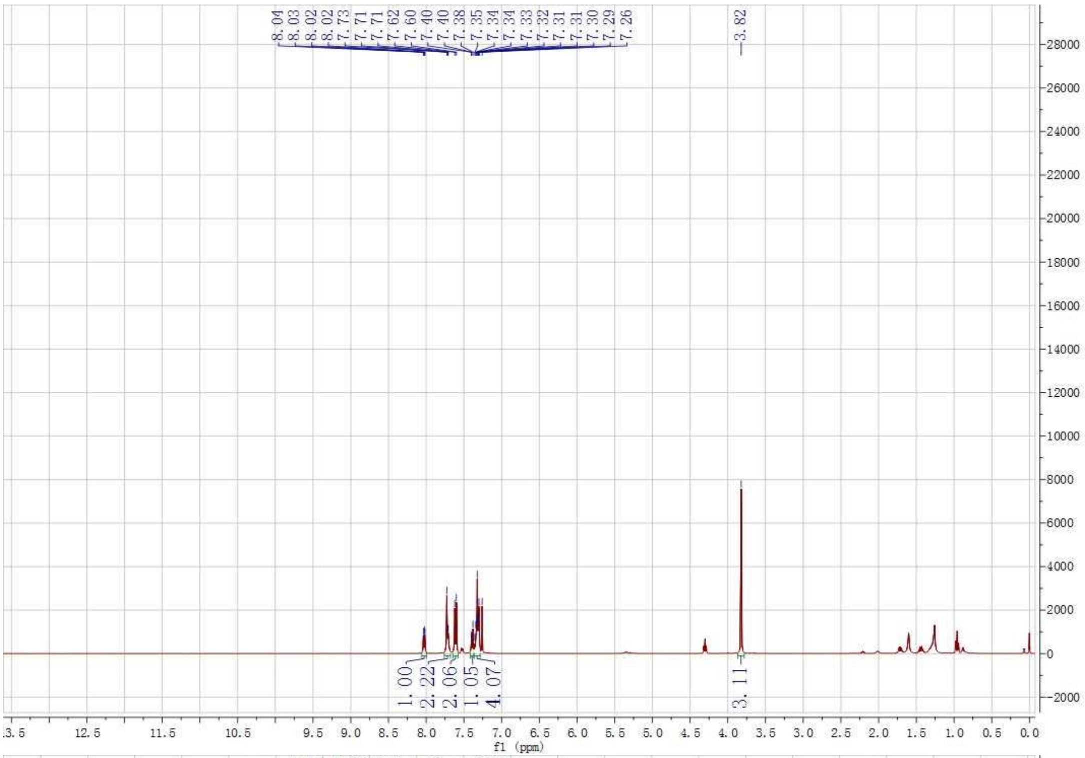

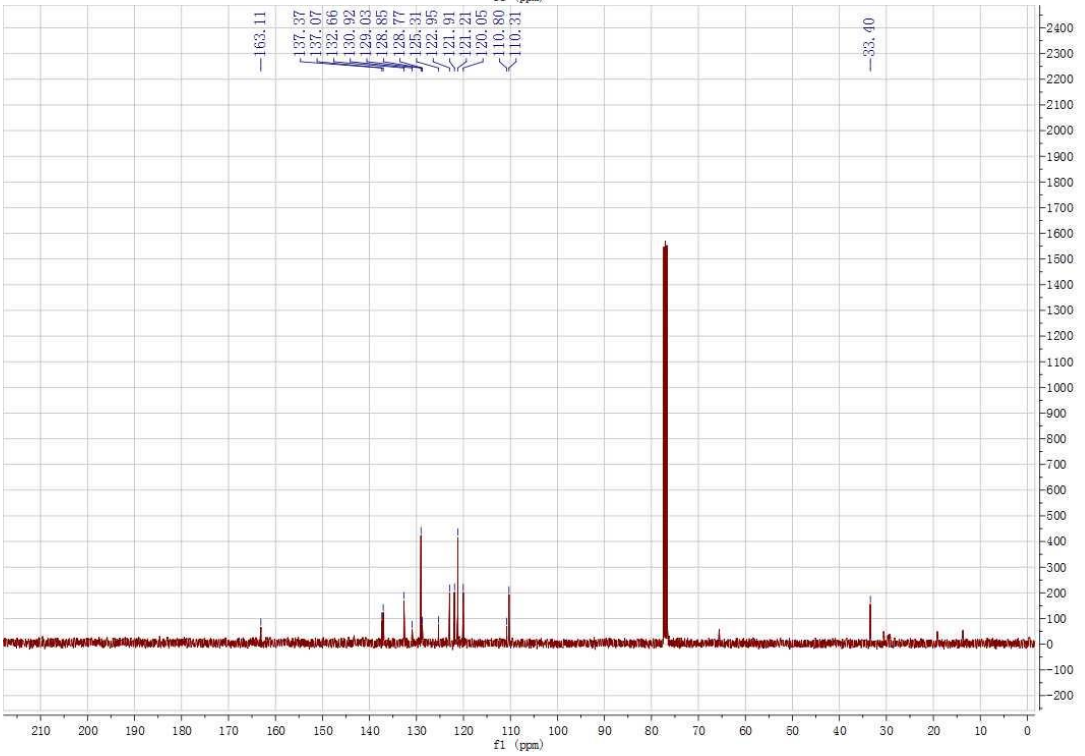

Supplementary Figure 44. ^{1}H and ^{13}C NMR spectrum for **3aj**

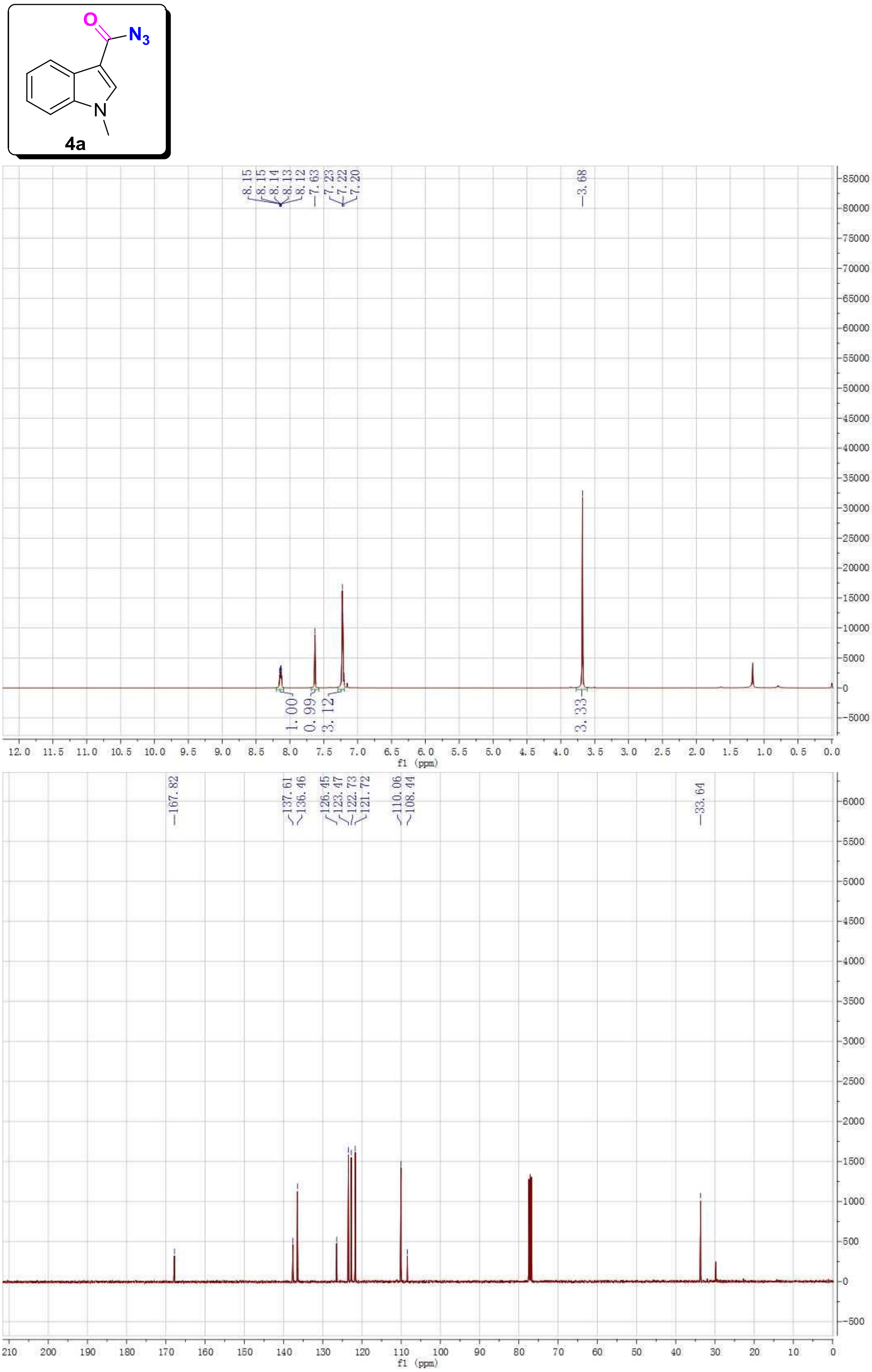

Supplementary Figure 45. ^{1}H and ^{13}C NMR spectrum for **4a**

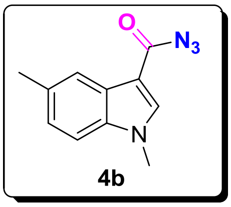

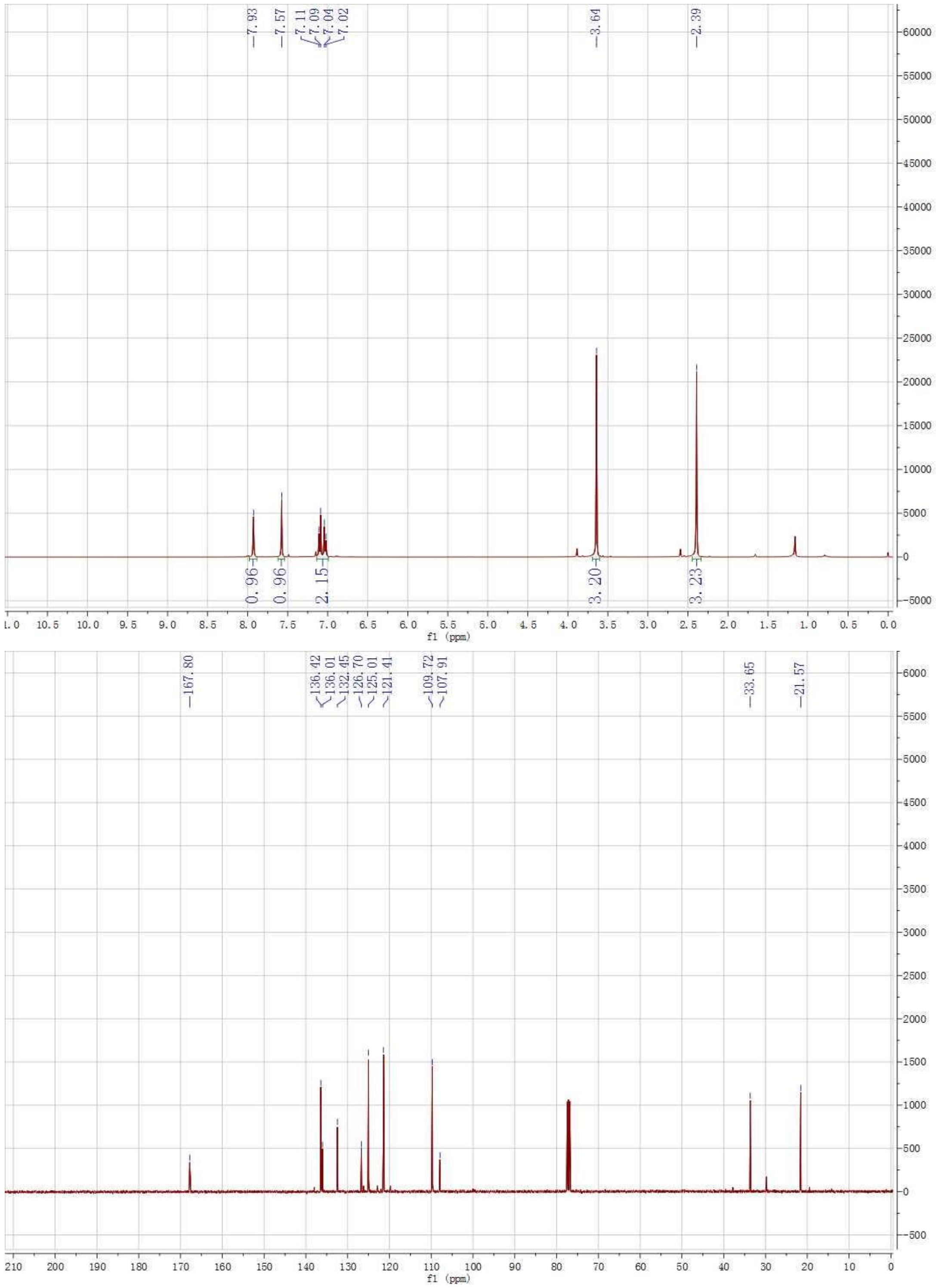

Supplementary Figure 46. ^{1}H and ^{13}C NMR spectrum for **4b**

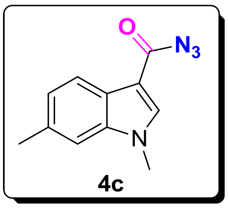

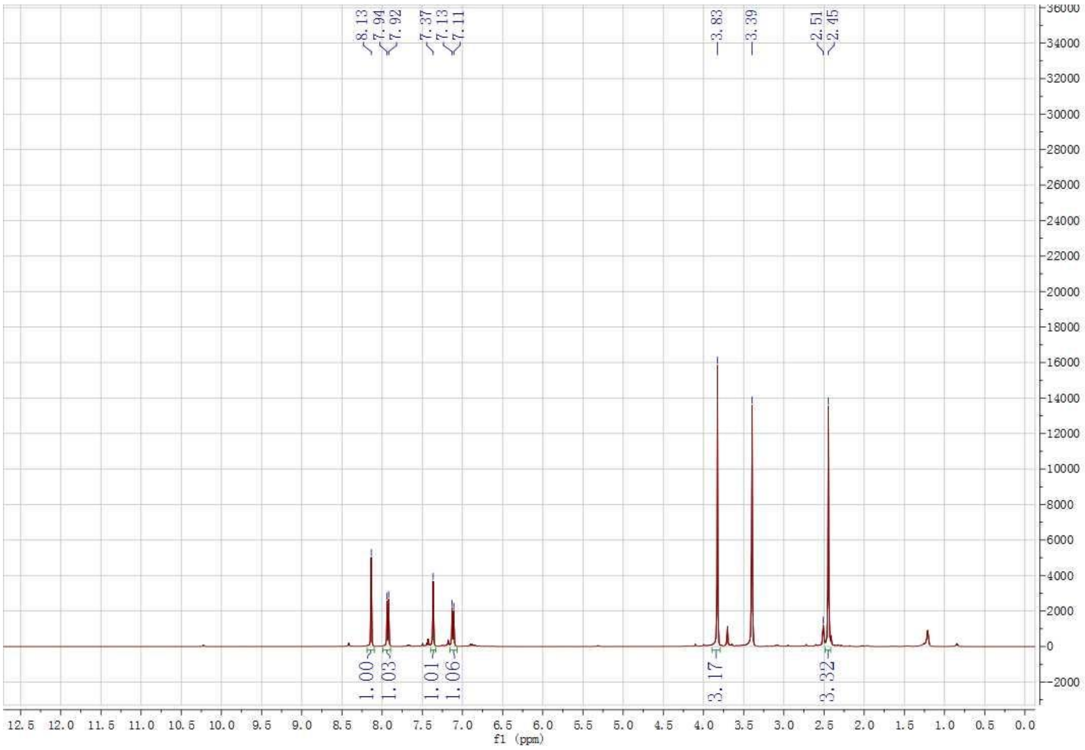

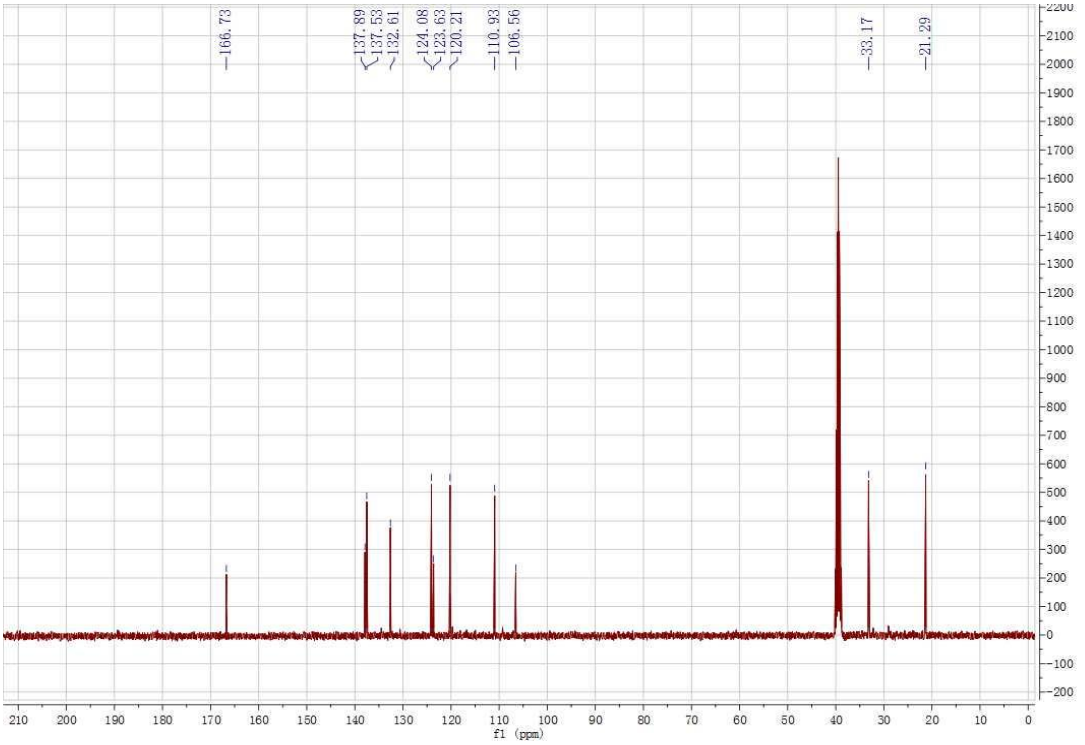

Supplementary Figure 47. ^{1}H and ^{13}C NMR spectrum for **4c**

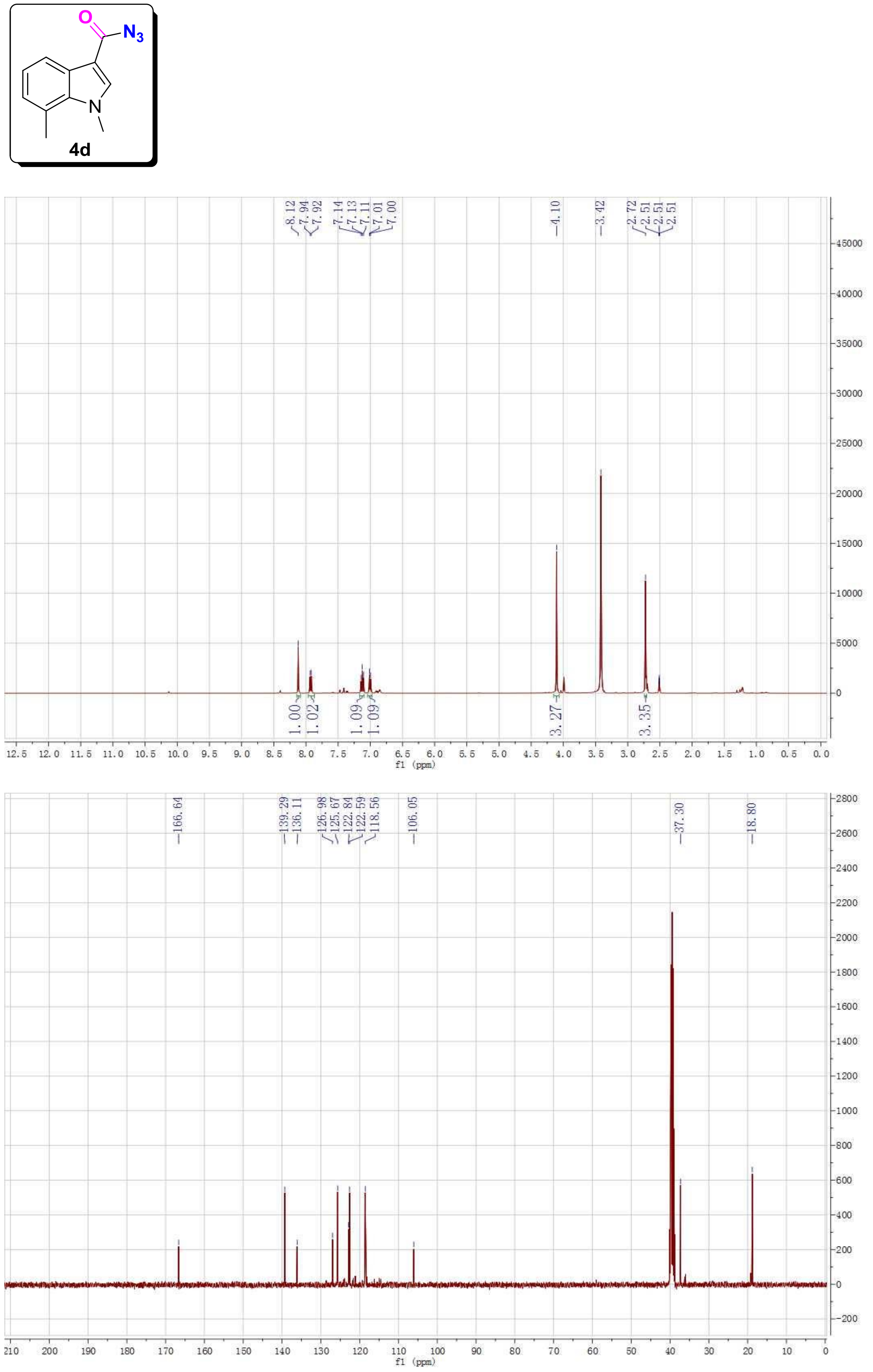

Supplementary Figure 48. ^{1}H and ^{13}C NMR spectrum for **4d**

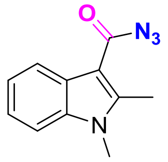

4e

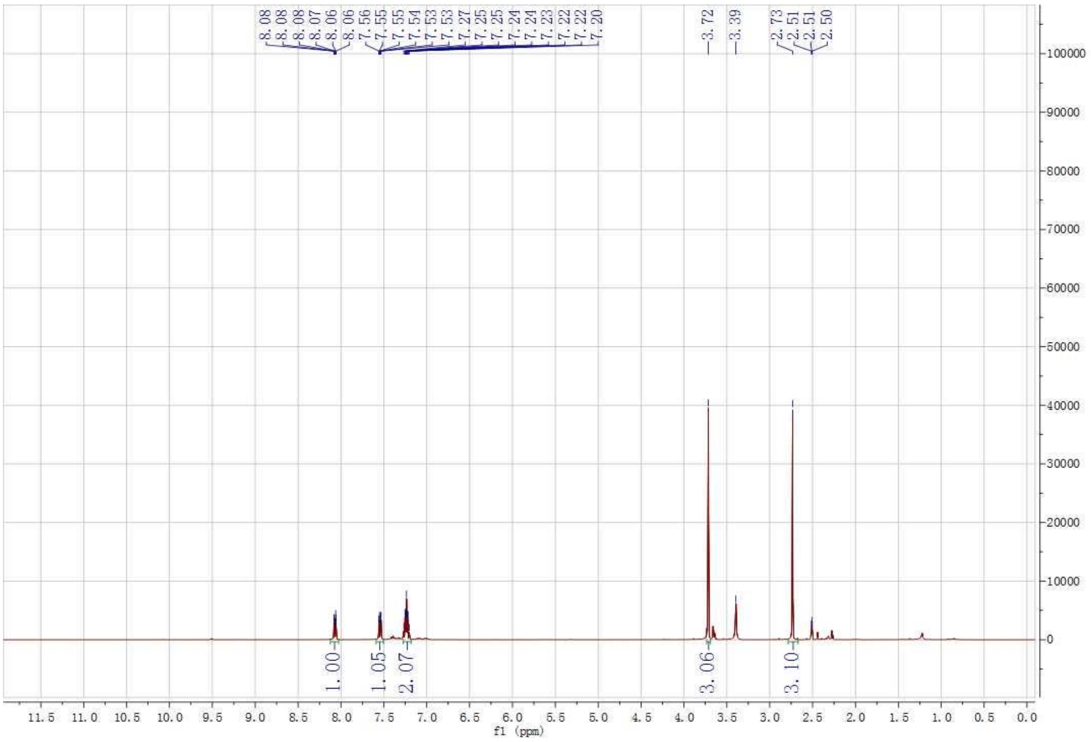

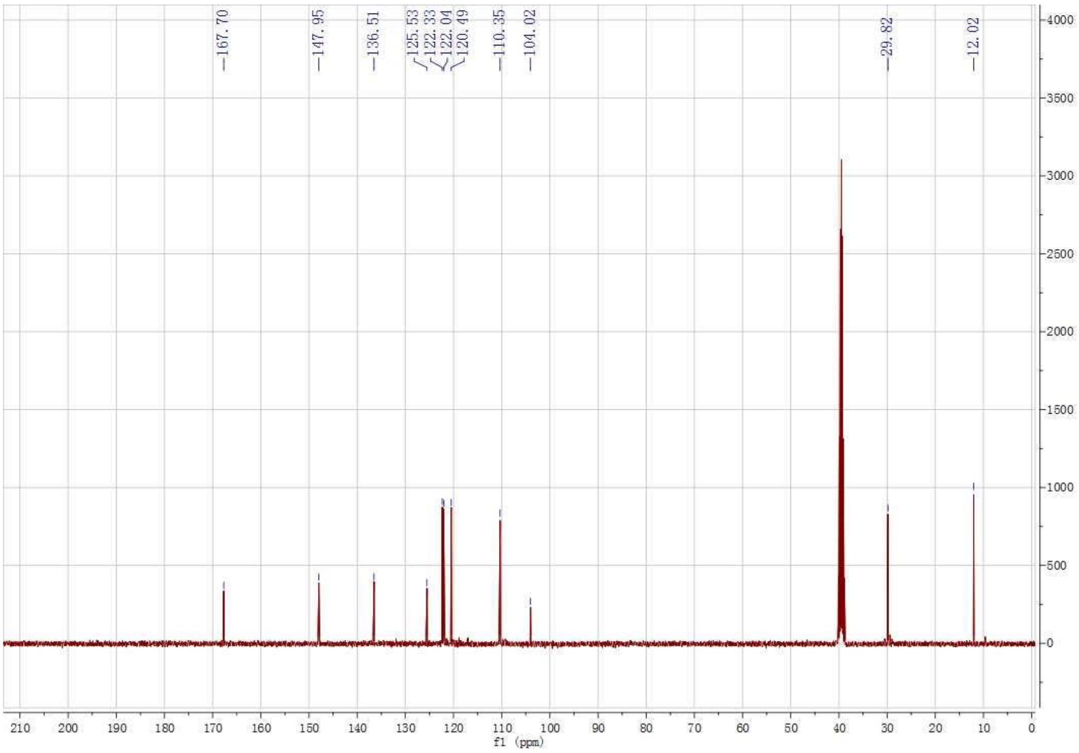

Supplementary Figure 49. ^{1}H and ^{13}C NMR spectrum for **4e**

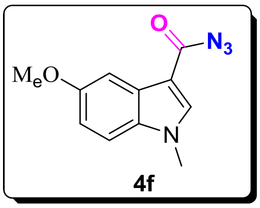

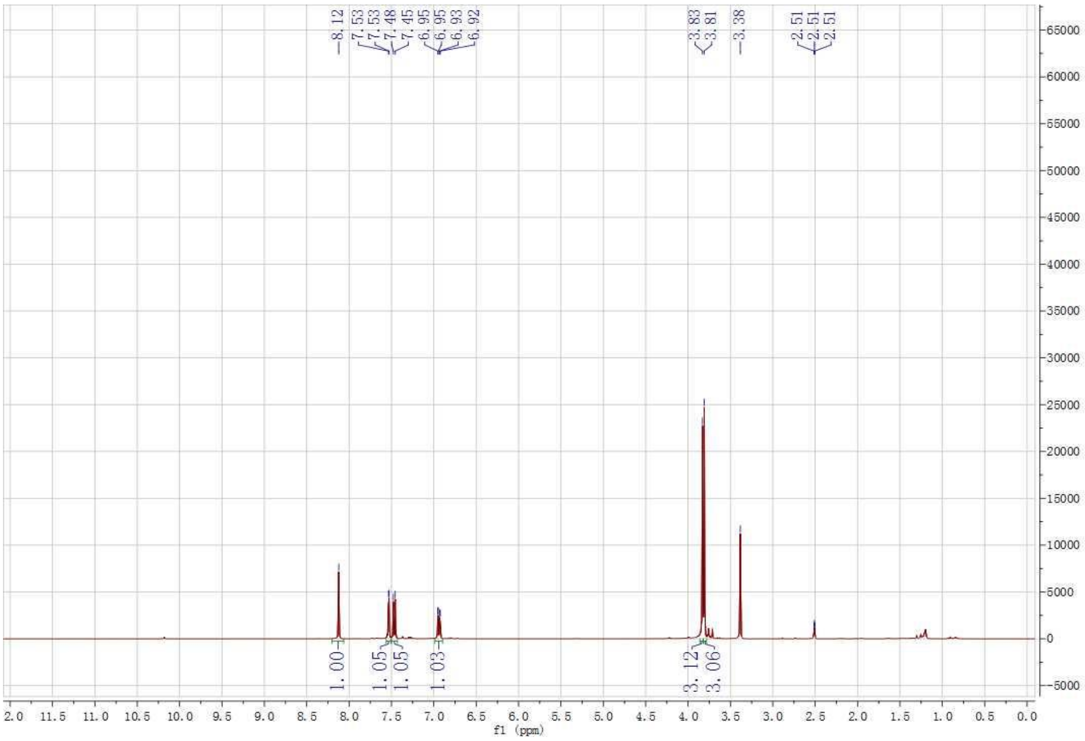

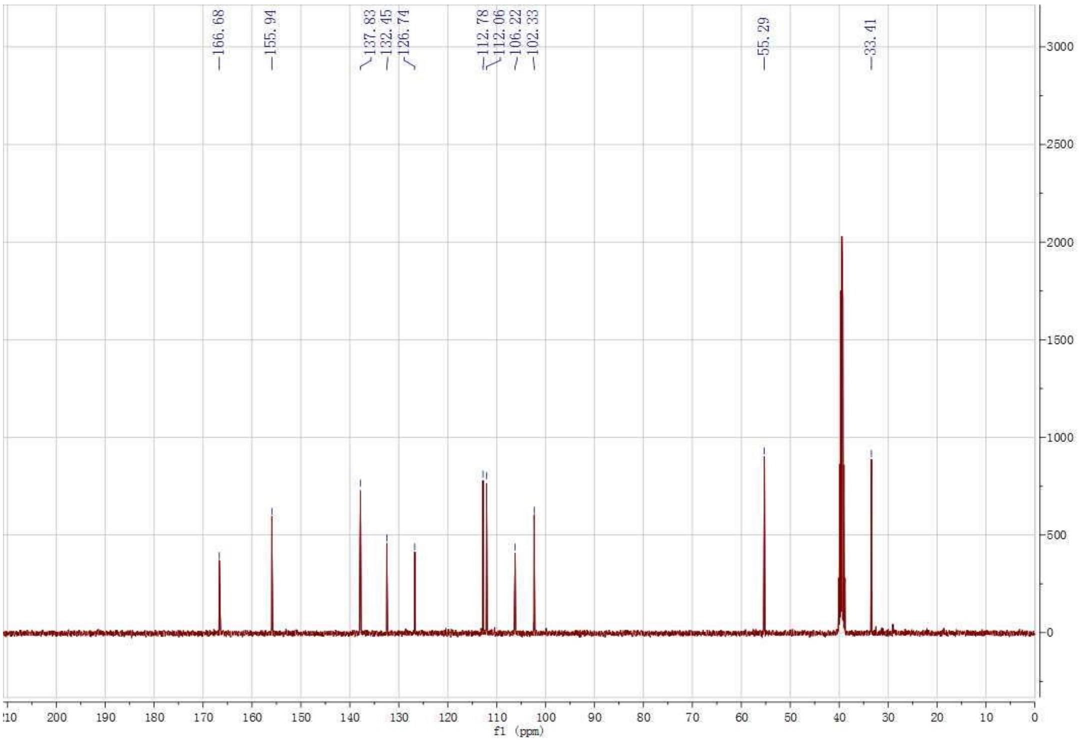

Supplementary Figure 50. ^{1}H and ^{13}C NMR spectrum for **4f**

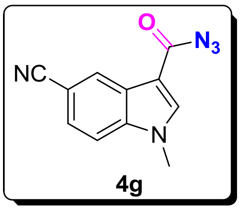

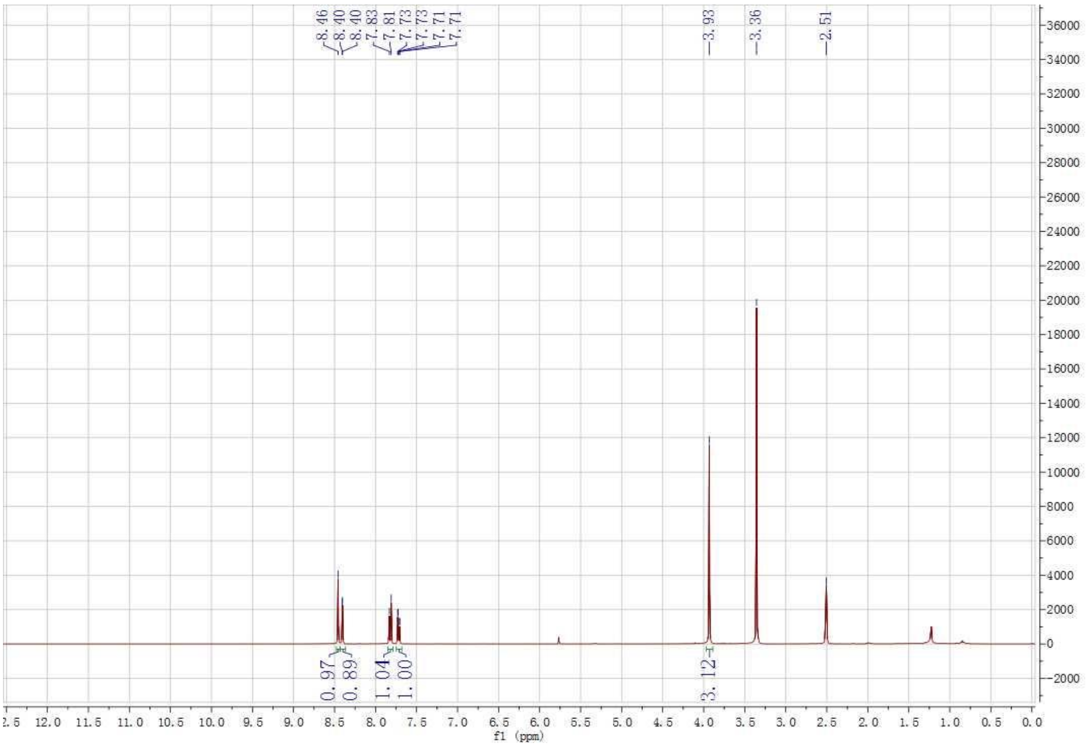

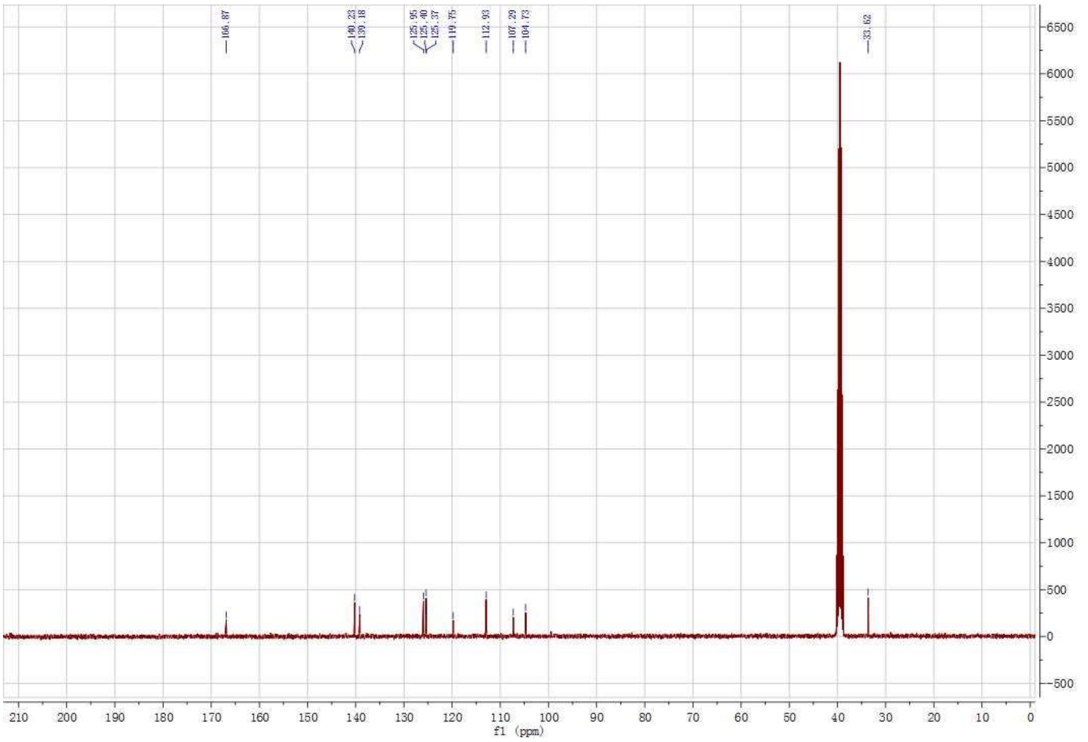

Supplementary Figure 51. ^{1}H and ^{13}C NMR spectrum for **4g**

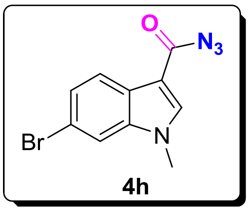

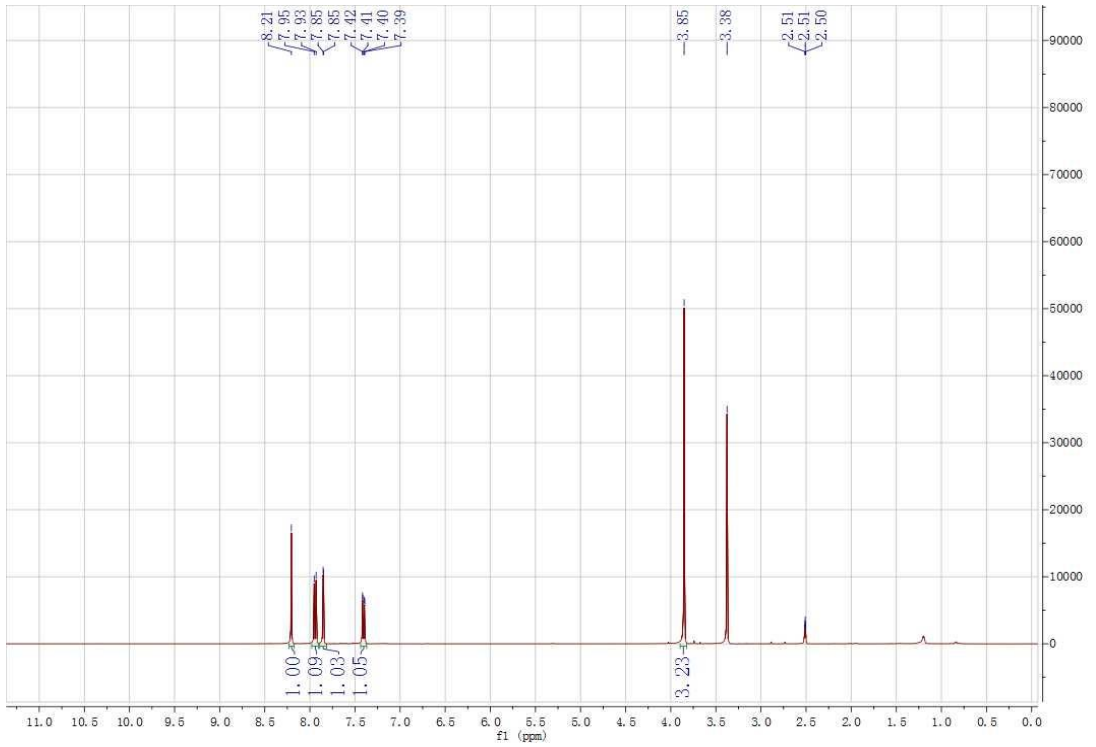

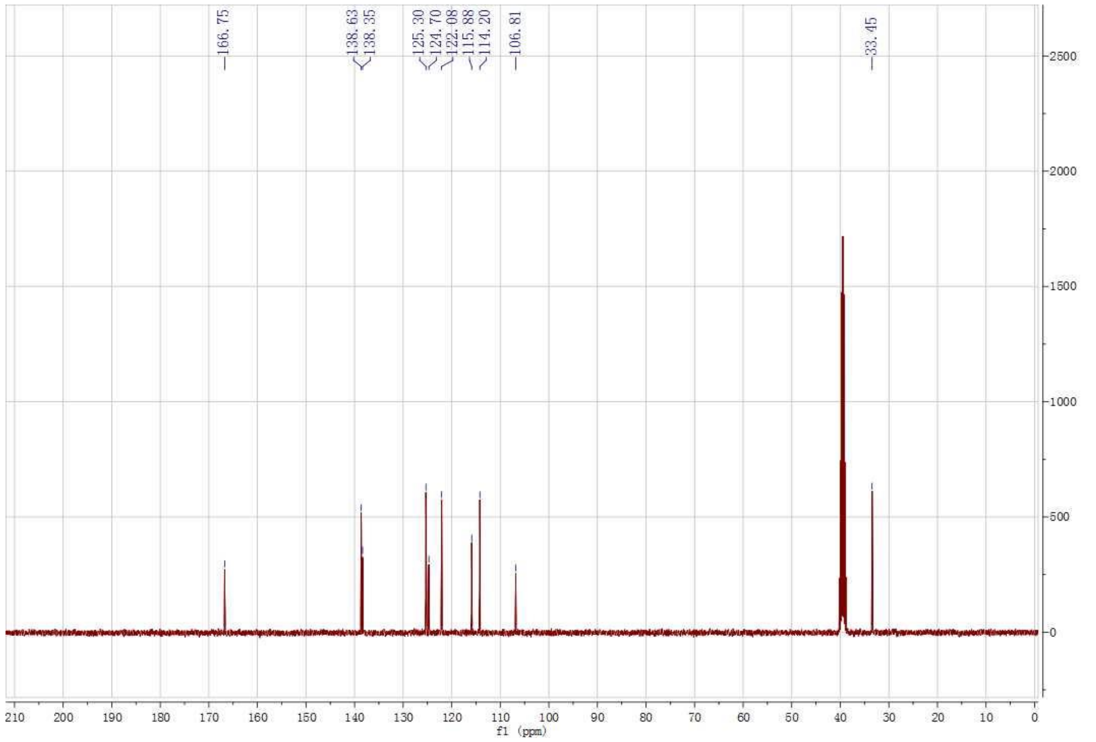

Supplementary Figure 52. ^{1}H and ^{13}C NMR spectrum for **4h**

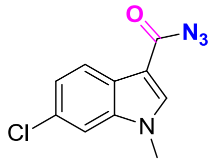

4i

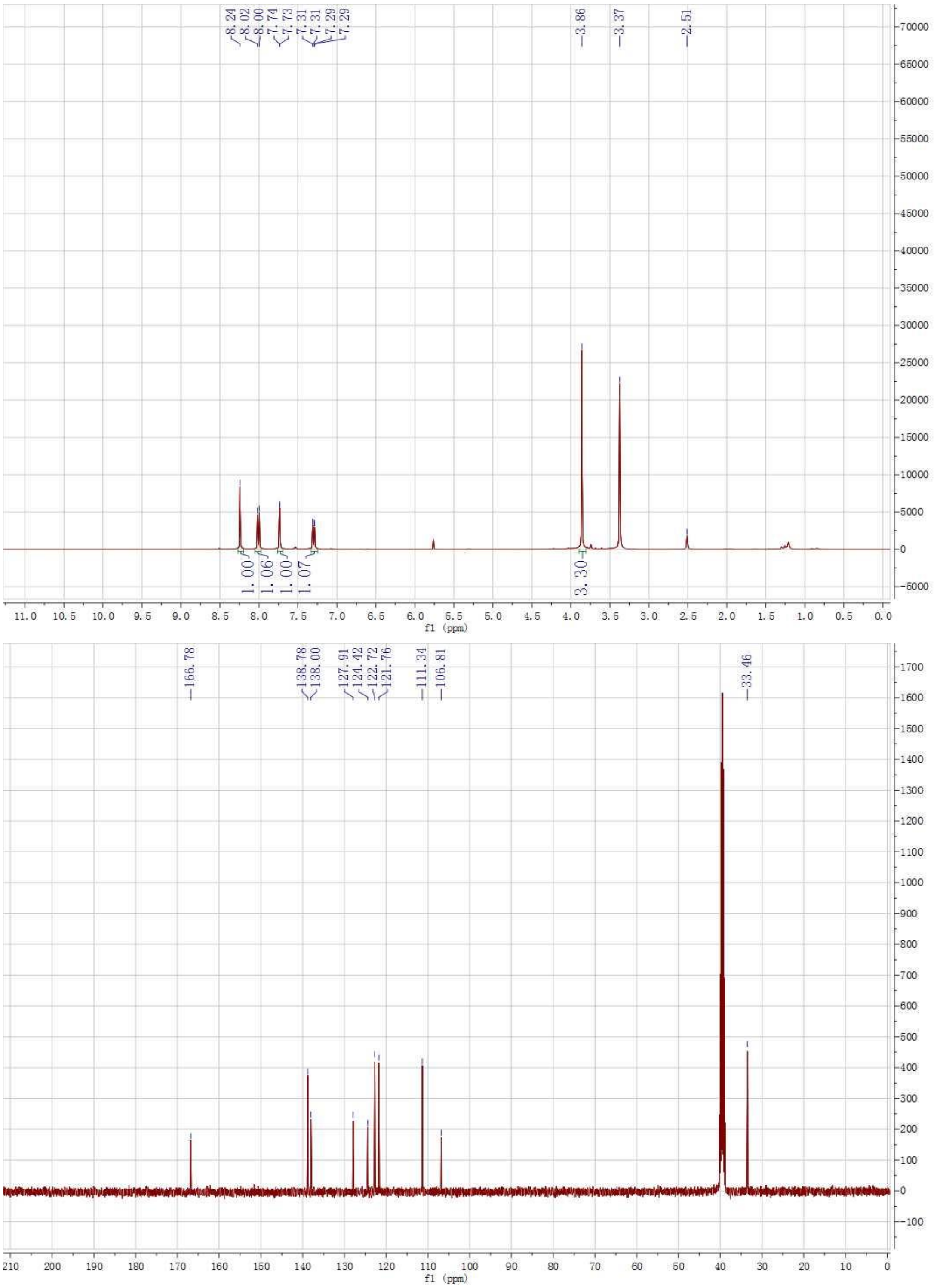

Supplementary Figure 53. ^{1}H and ^{13}C NMR spectrum for **4i**

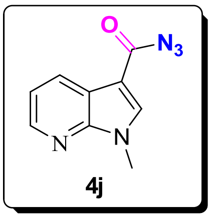

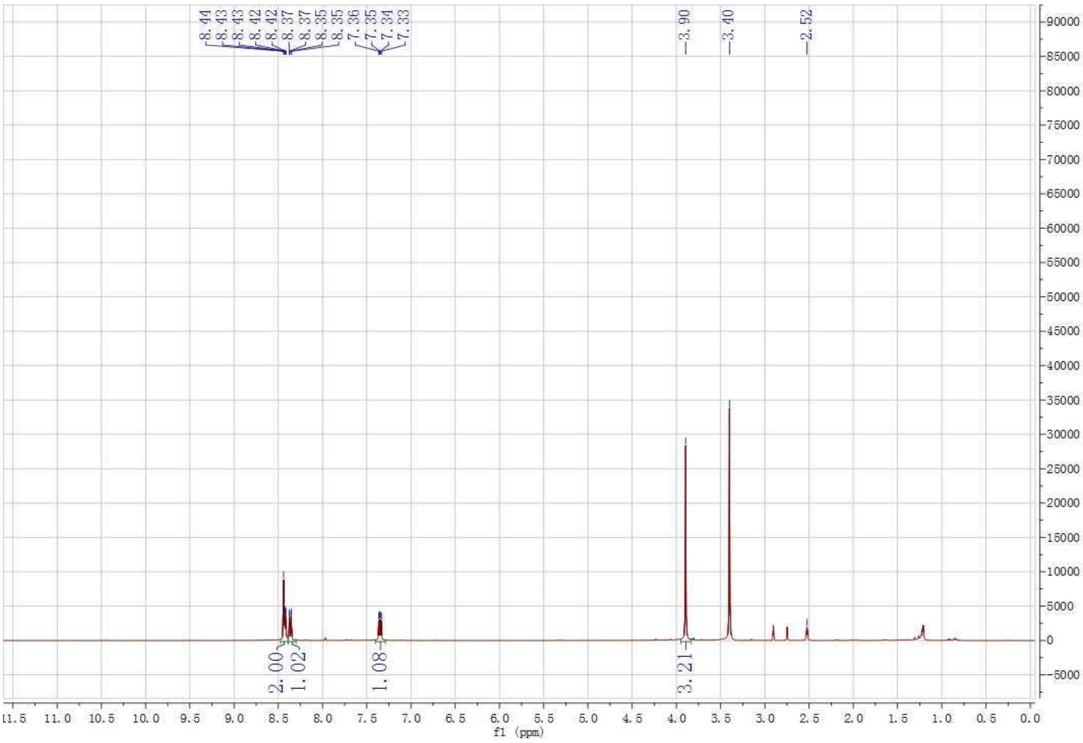

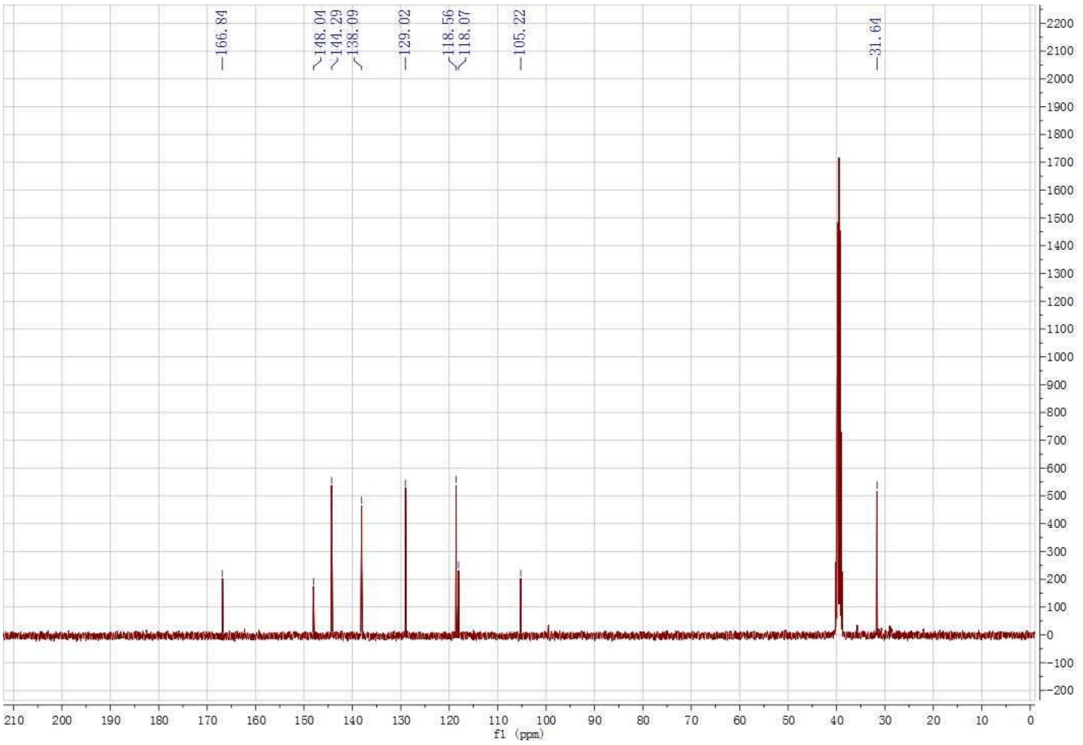

Supplementary Figure 54. ^{1}H and ^{13}C NMR spectrum for **4j**

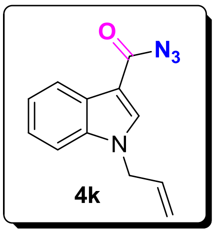

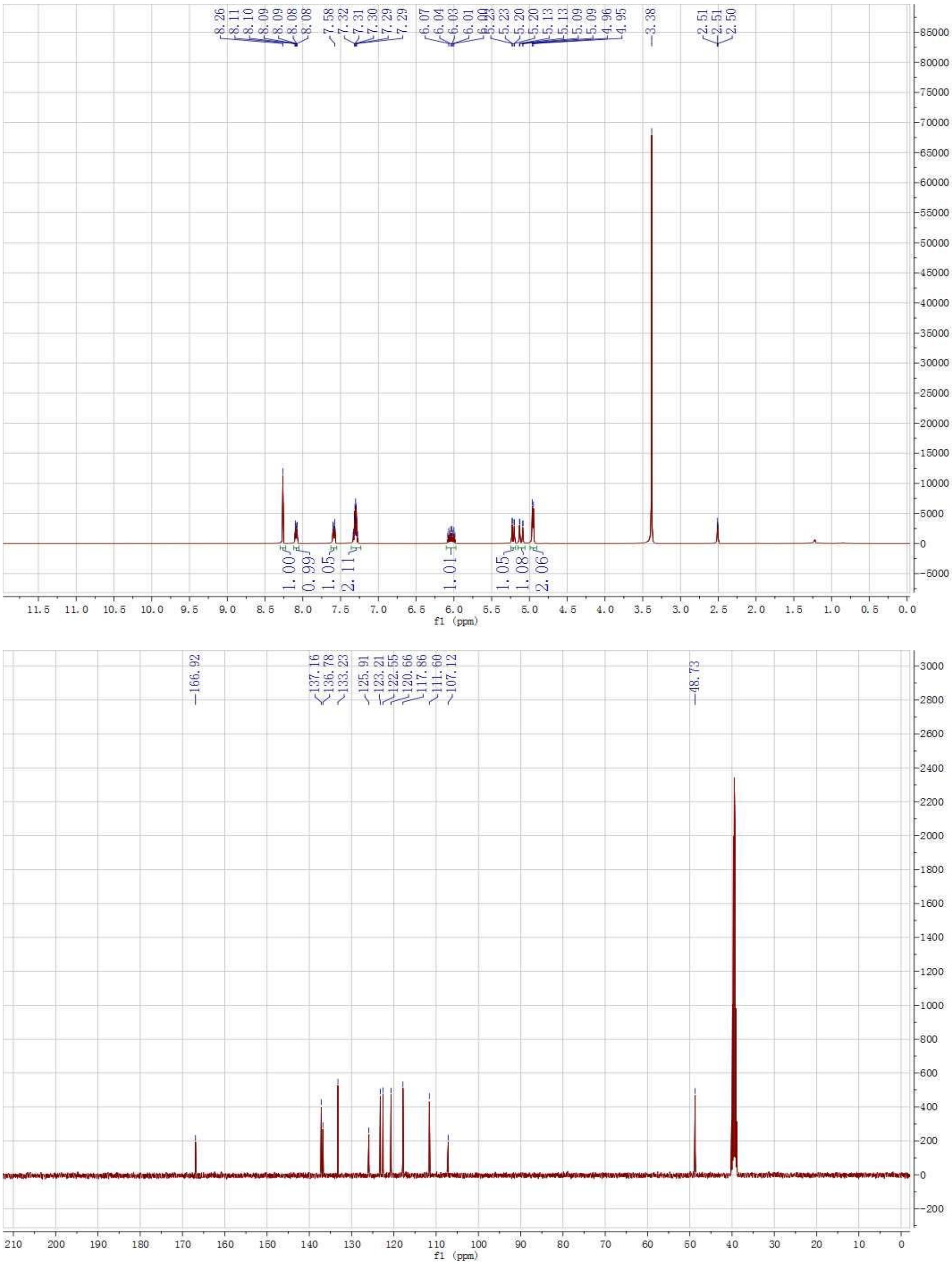

Supplementary Figure 55. ^{1}H and ^{13}C NMR spectrum for **4k**

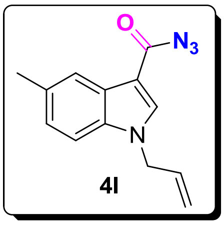

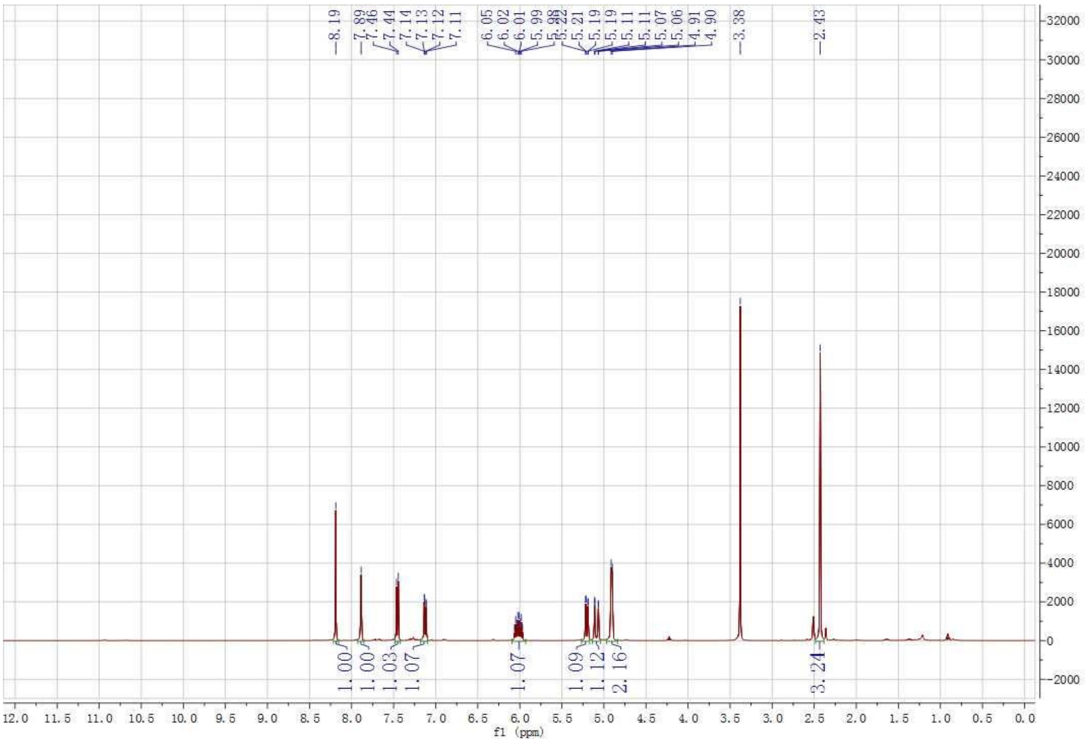

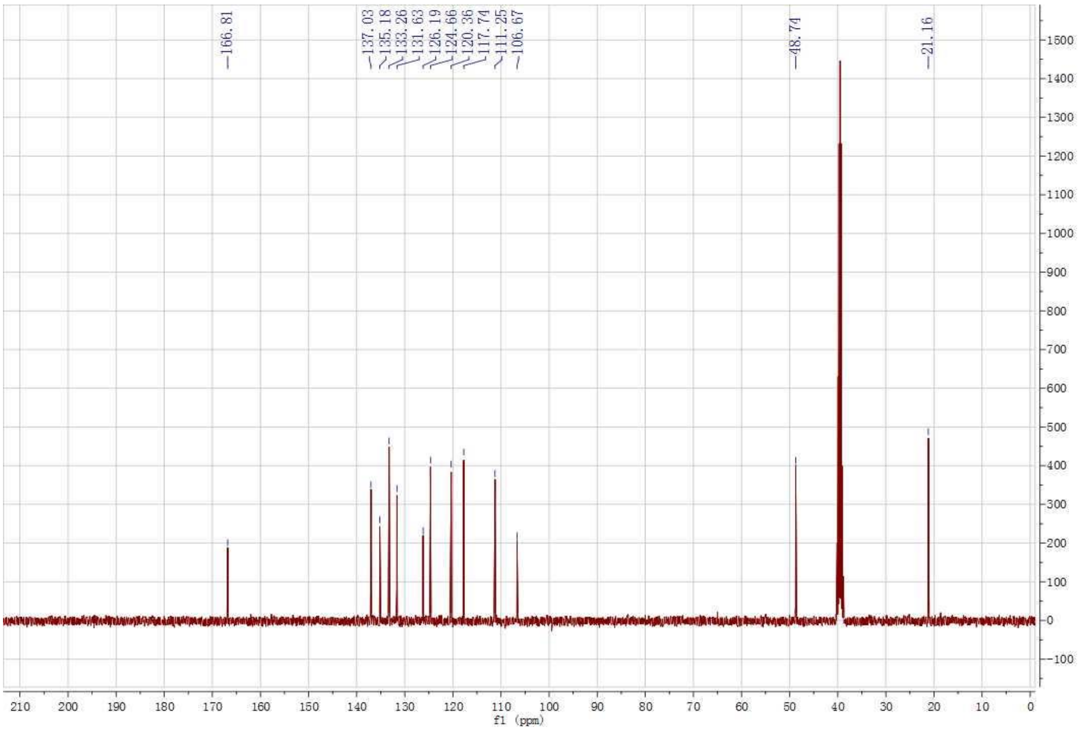

Supplementary Figure 56. ^{1}H and ^{13}C NMR spectrum for **4l**

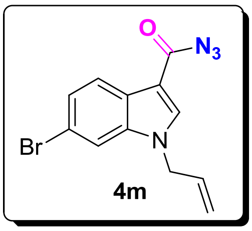

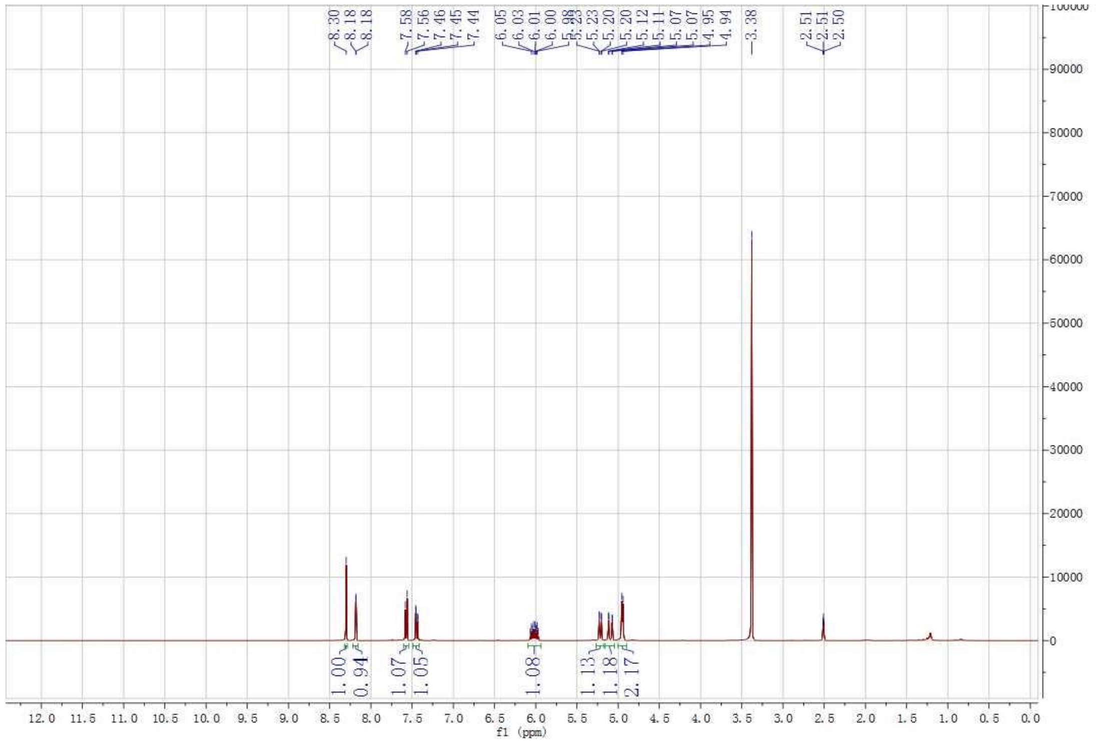

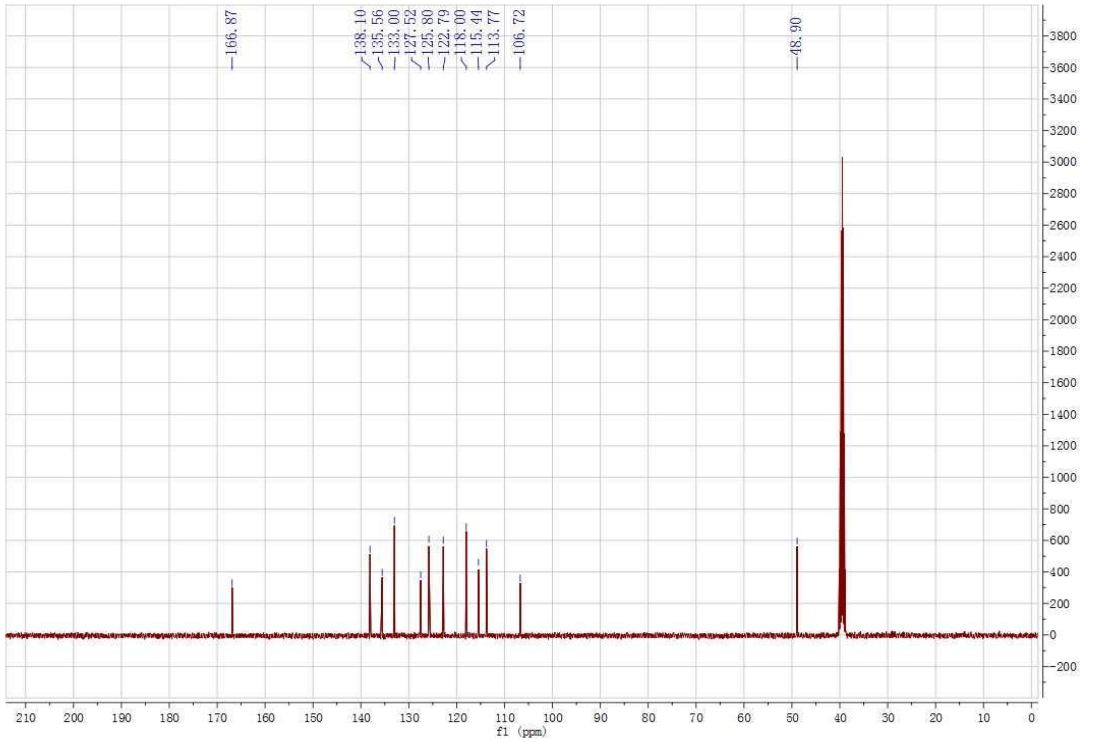

Supplementary Figure 57. ^{1}H and ^{13}C NMR spectrum for **4m**

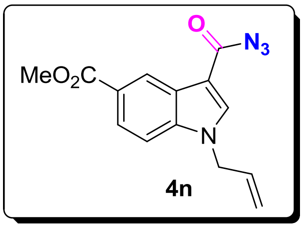

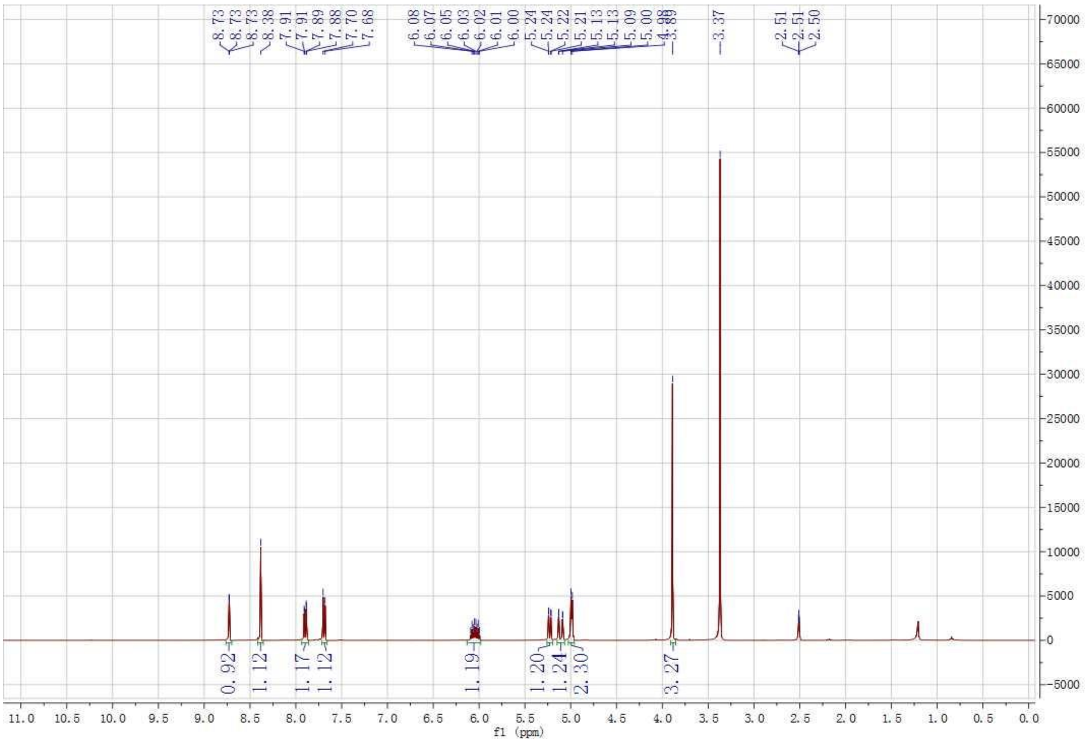

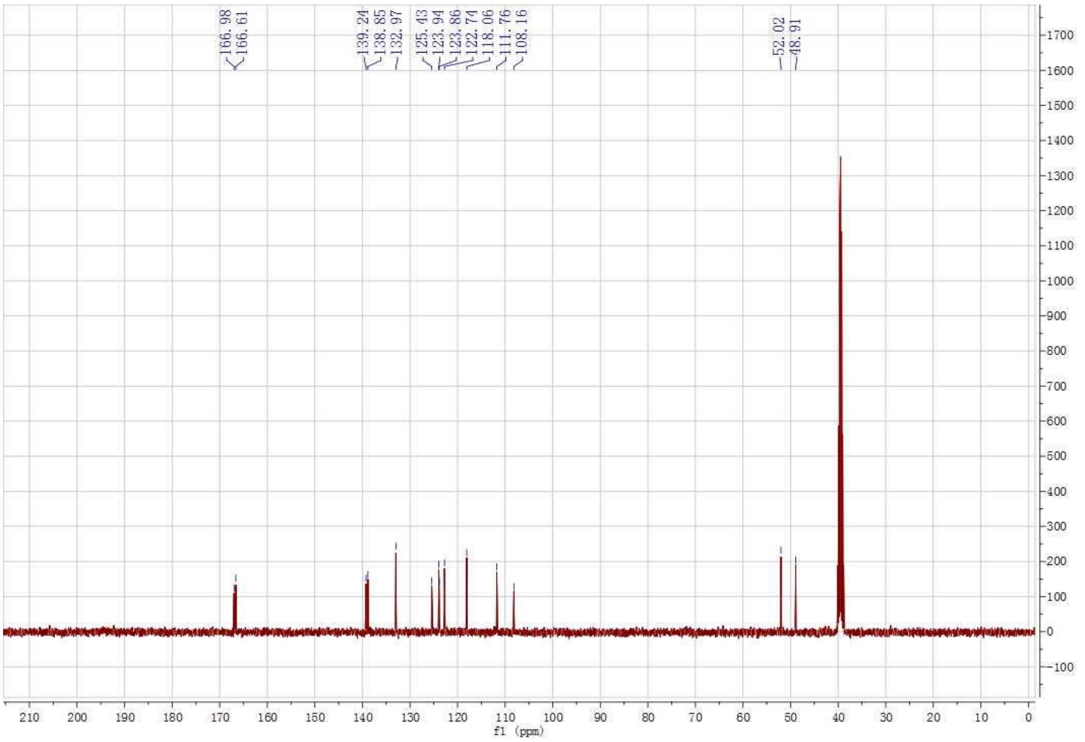

Supplementary Figure 58. ^{1}H and ^{13}C NMR spectrum for **4n**

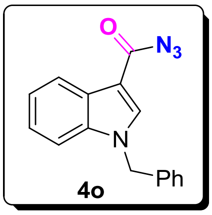

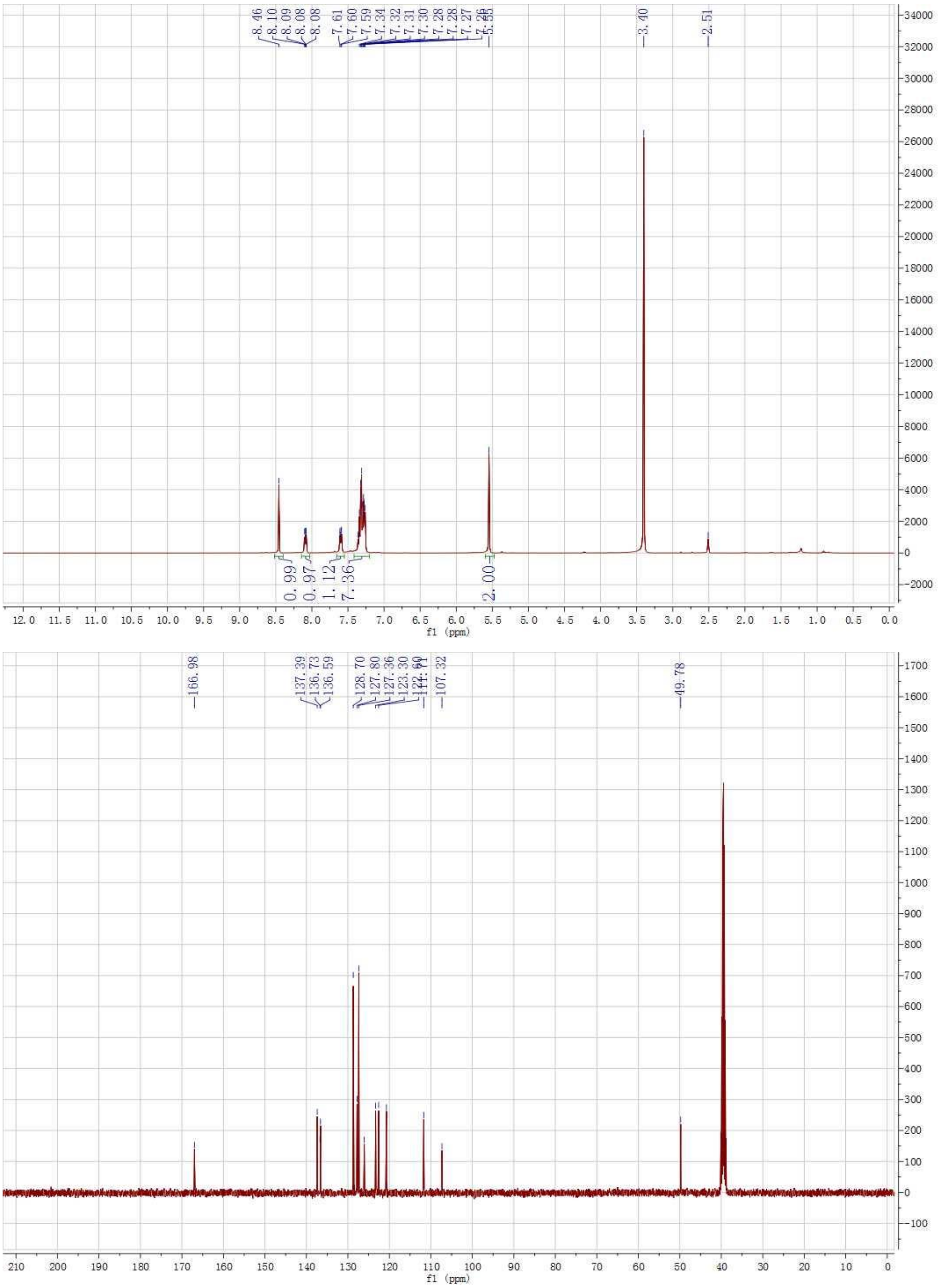

Supplementary Figure 59. ^{1}H and ^{13}C NMR spectrum for **4o**

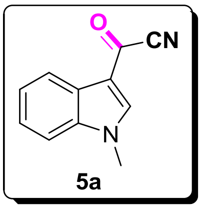

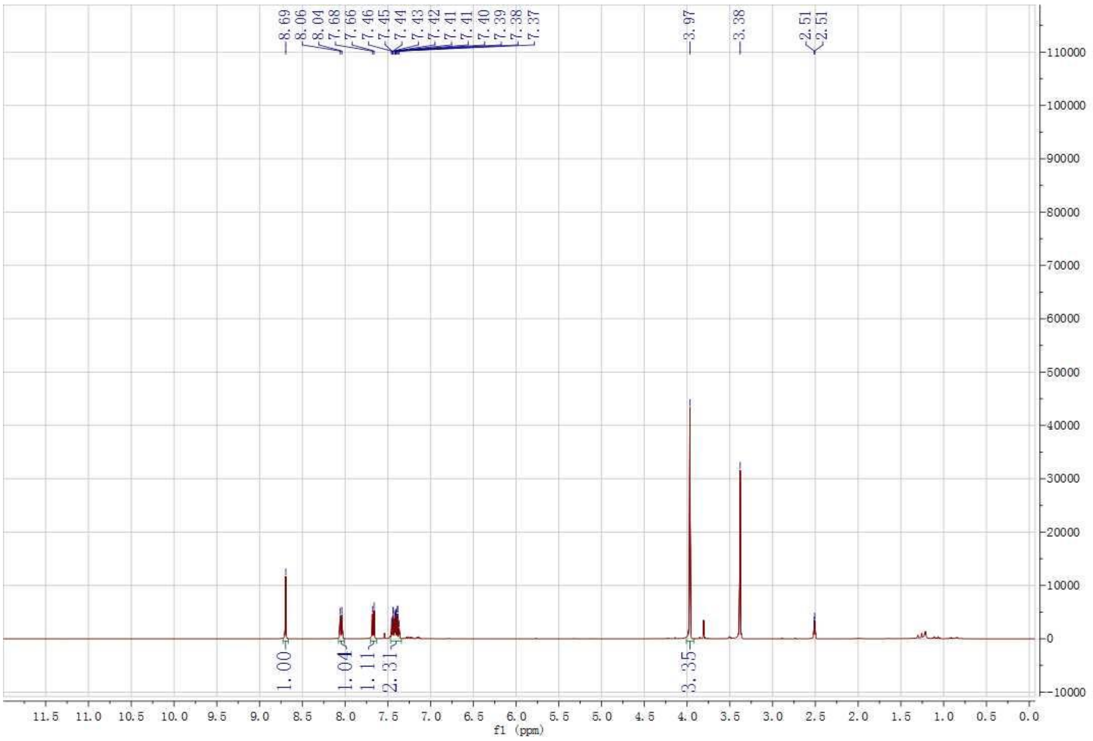

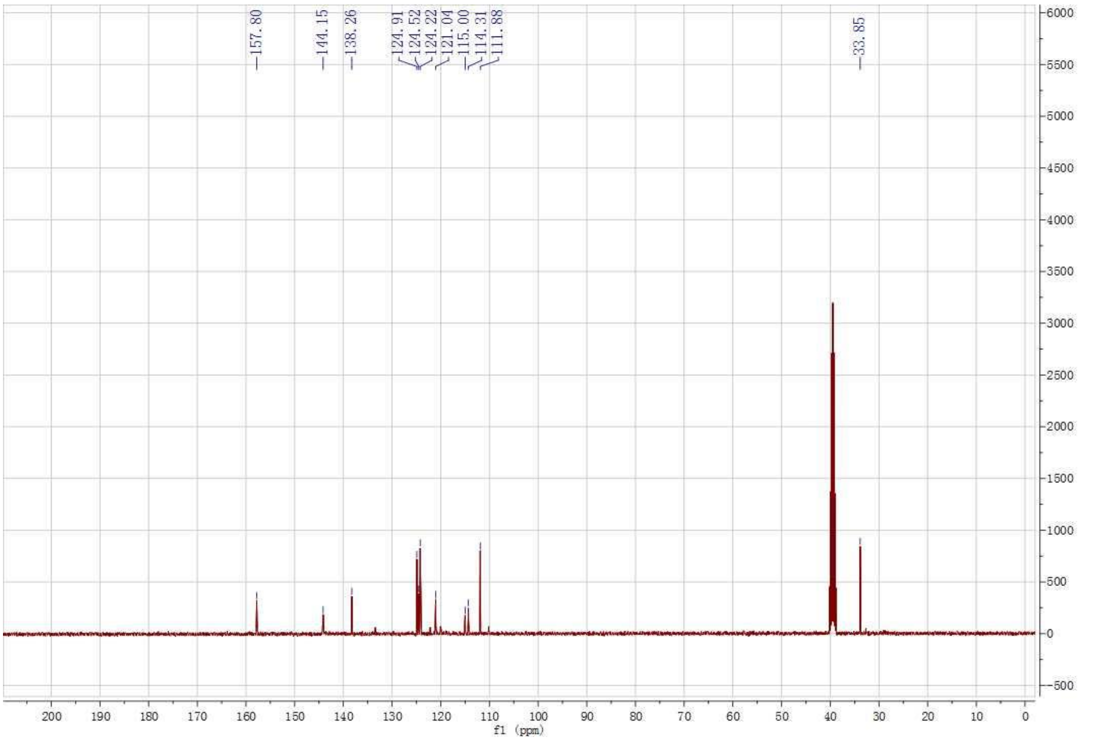

Supplementary Figure 60. ^{1}H and ^{13}C NMR spectrum for **5a**

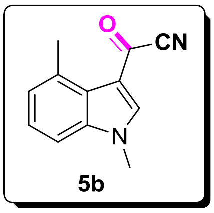

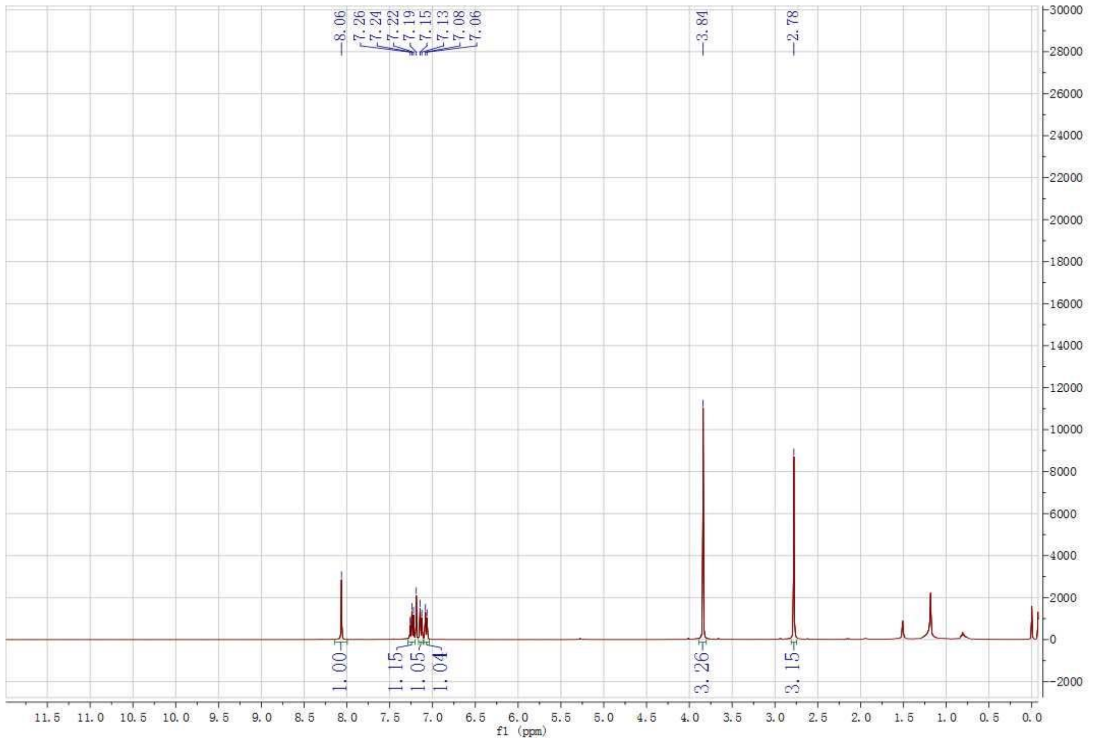

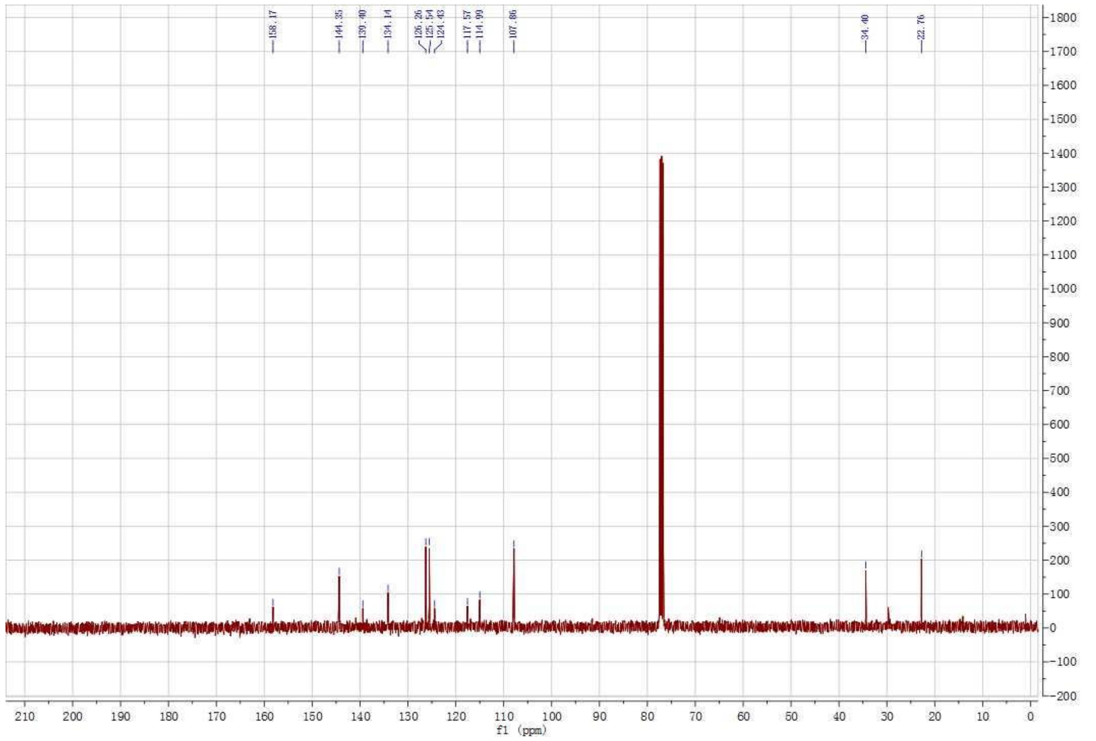

Supplementary Figure 61. ^{1}H and ^{13}C NMR spectrum for **5b**

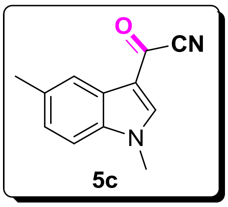

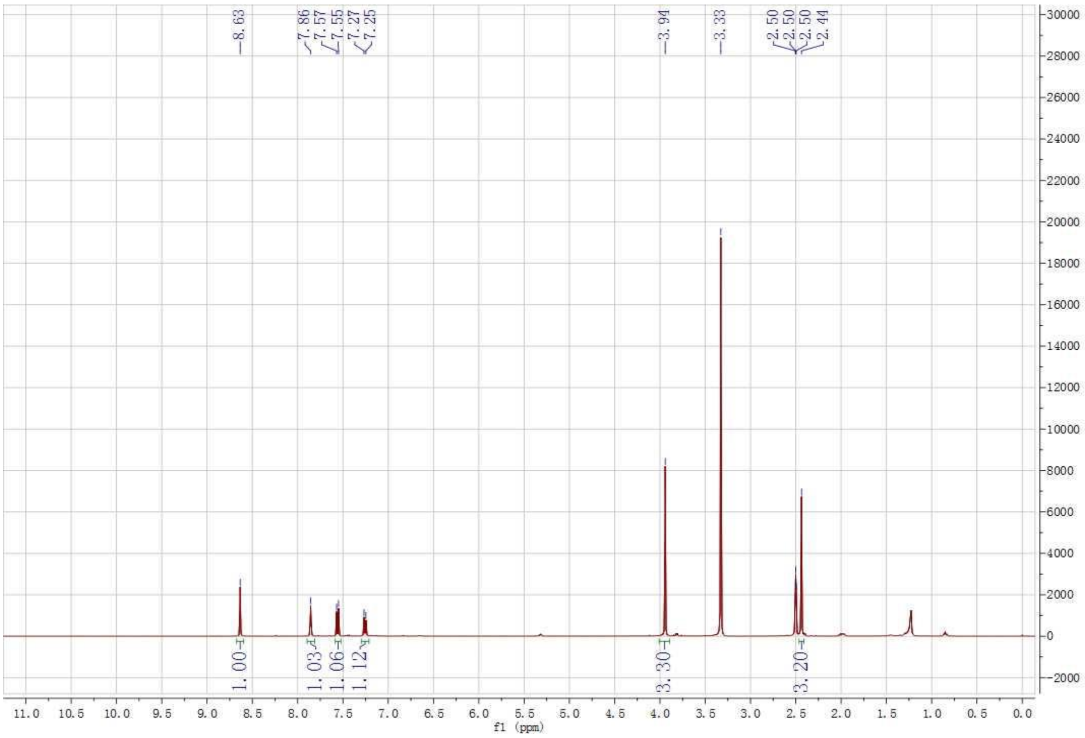

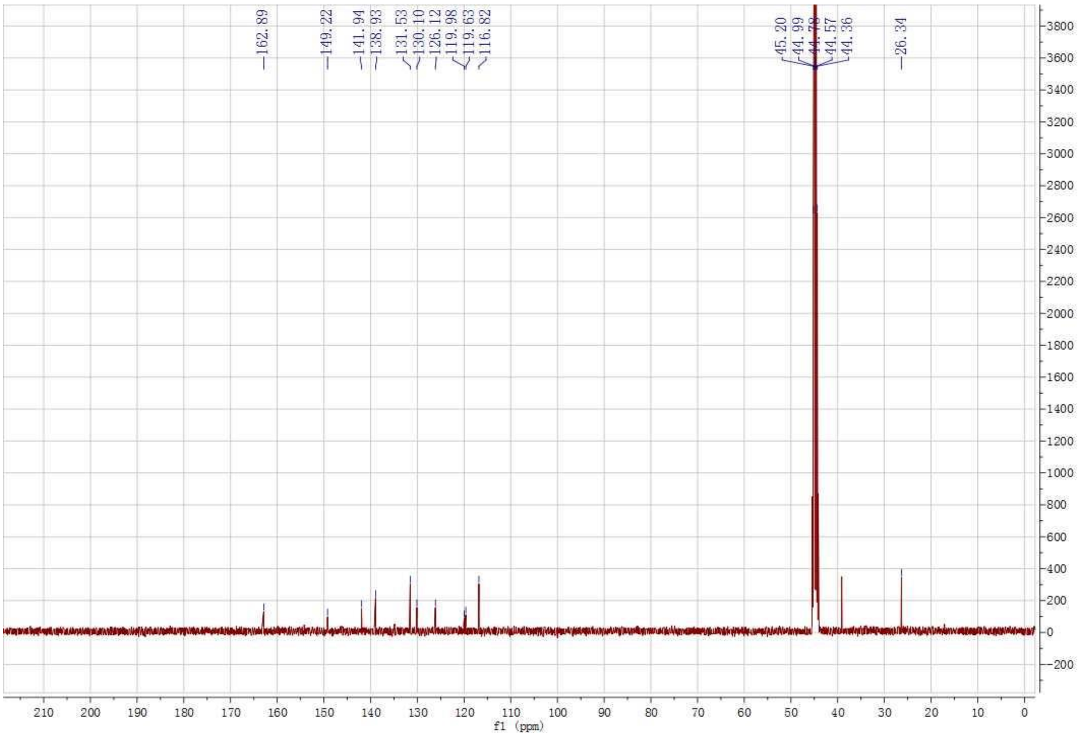

Supplementary Figure 62. ^{1}H and ^{13}C NMR spectrum for **5c**

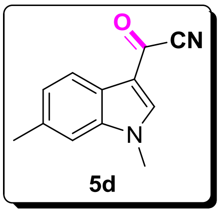

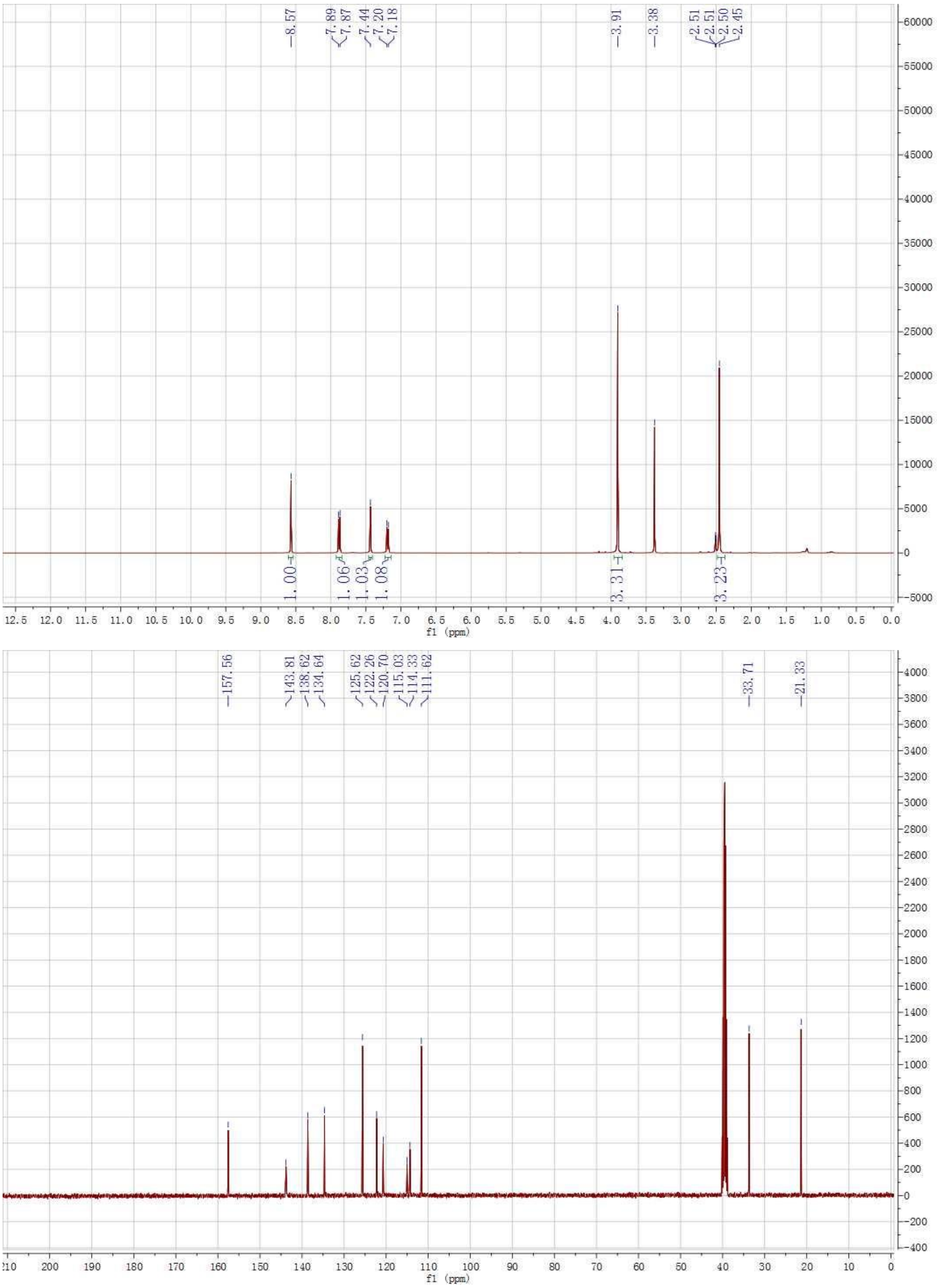

Supplementary Figure 63. ^{1}H and ^{13}C NMR spectrum for **5d**

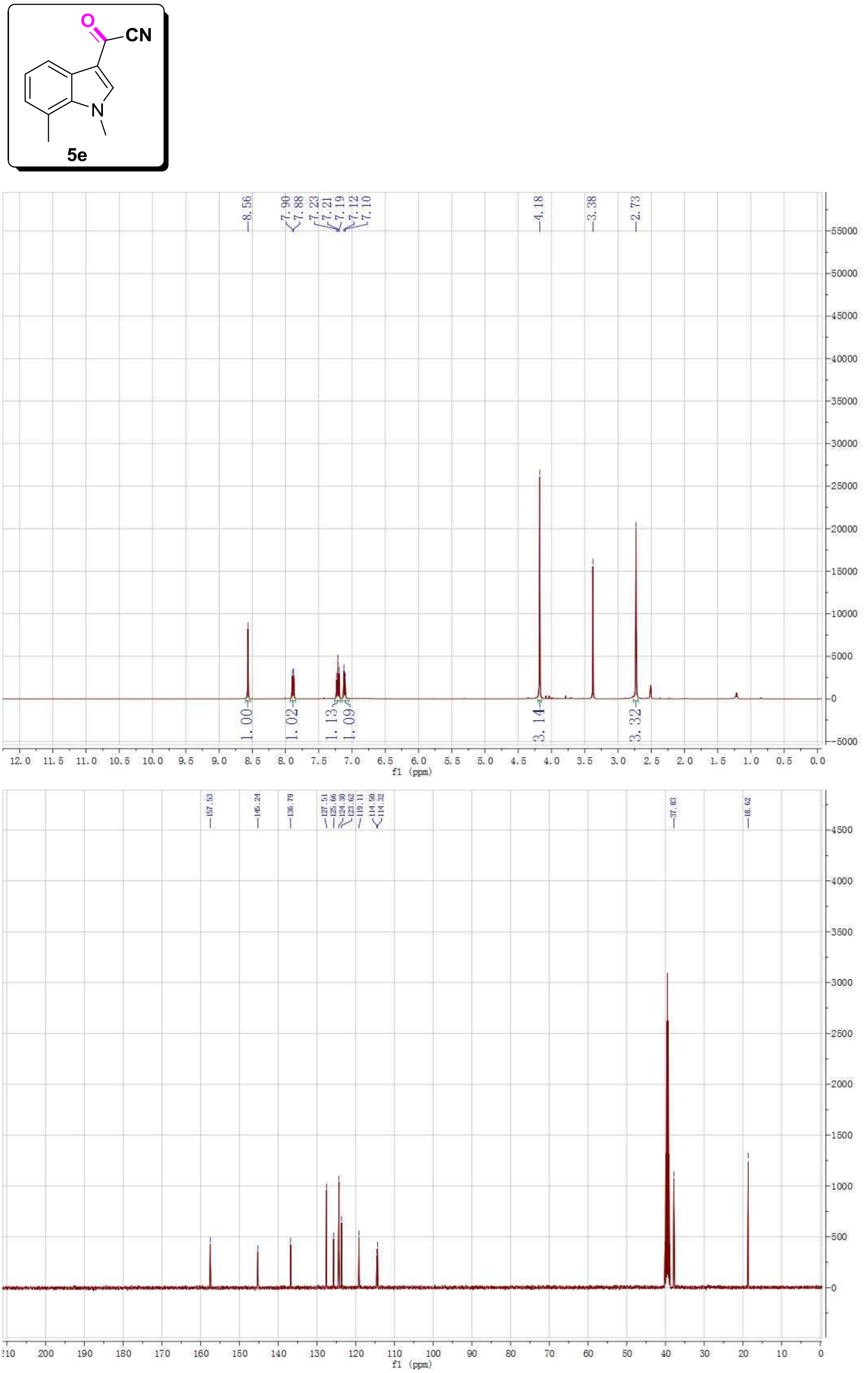

Supplementary Figure 64. ^{1}H and ^{13}C NMR spectrum for **5e**

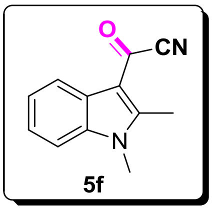

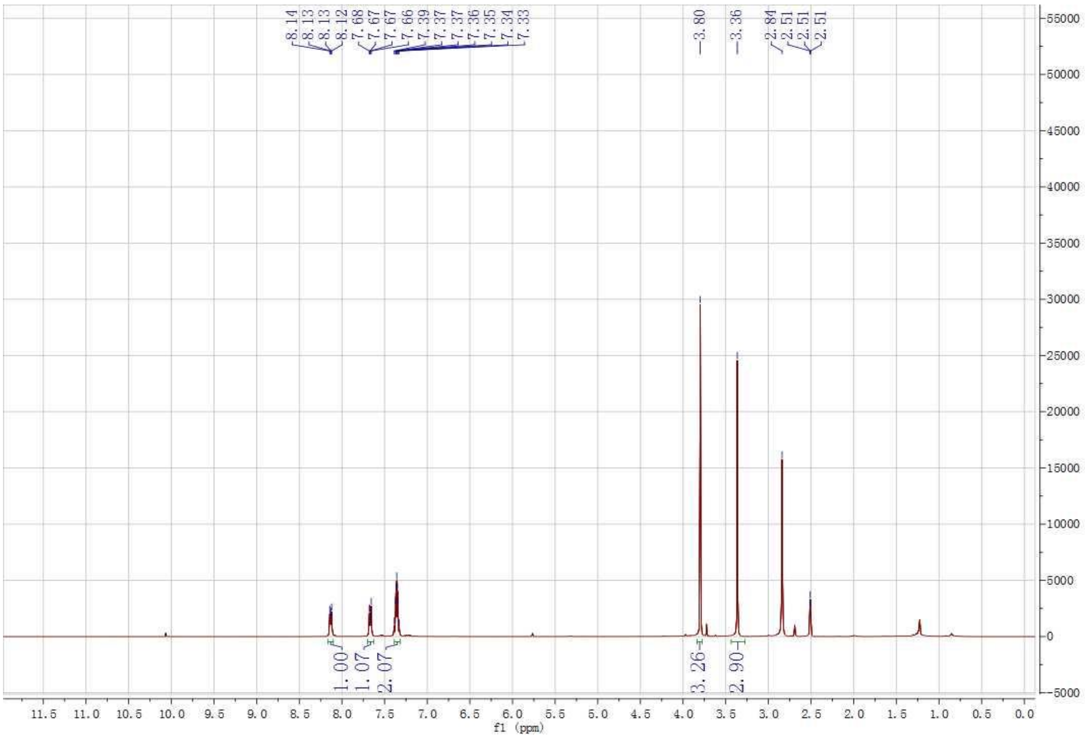

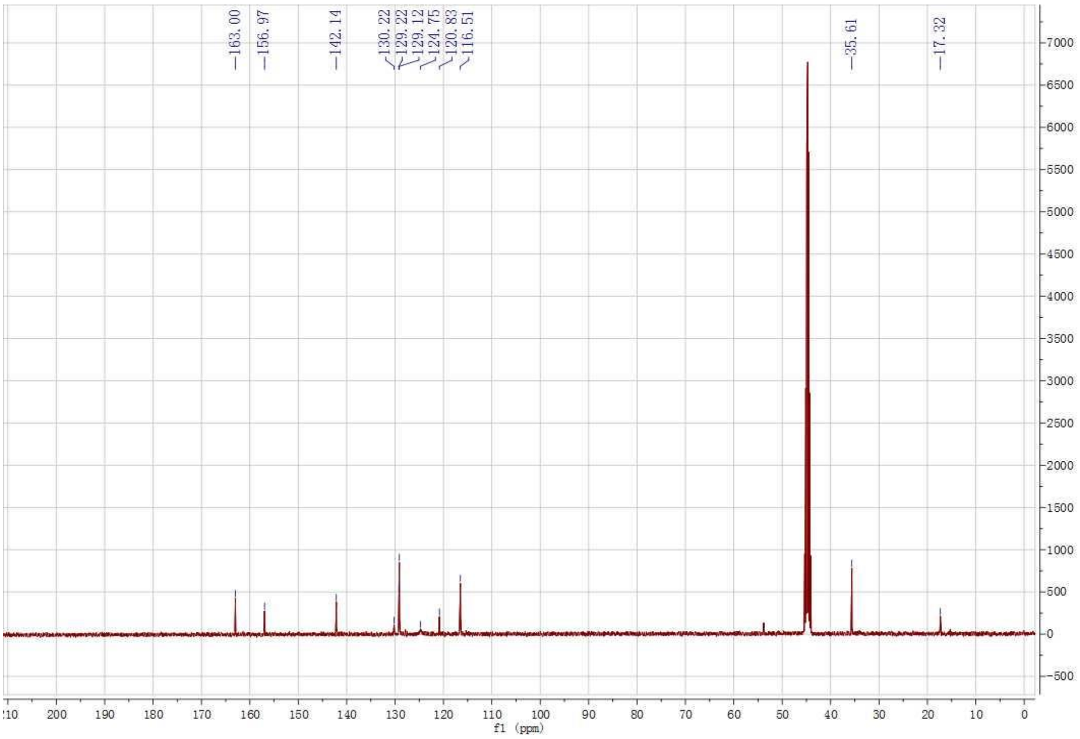

Supplementary Figure 65. ^{1}H and ^{13}C NMR spectrum for **5f**

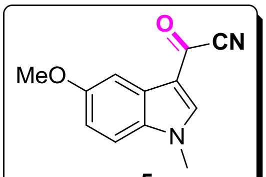

5g

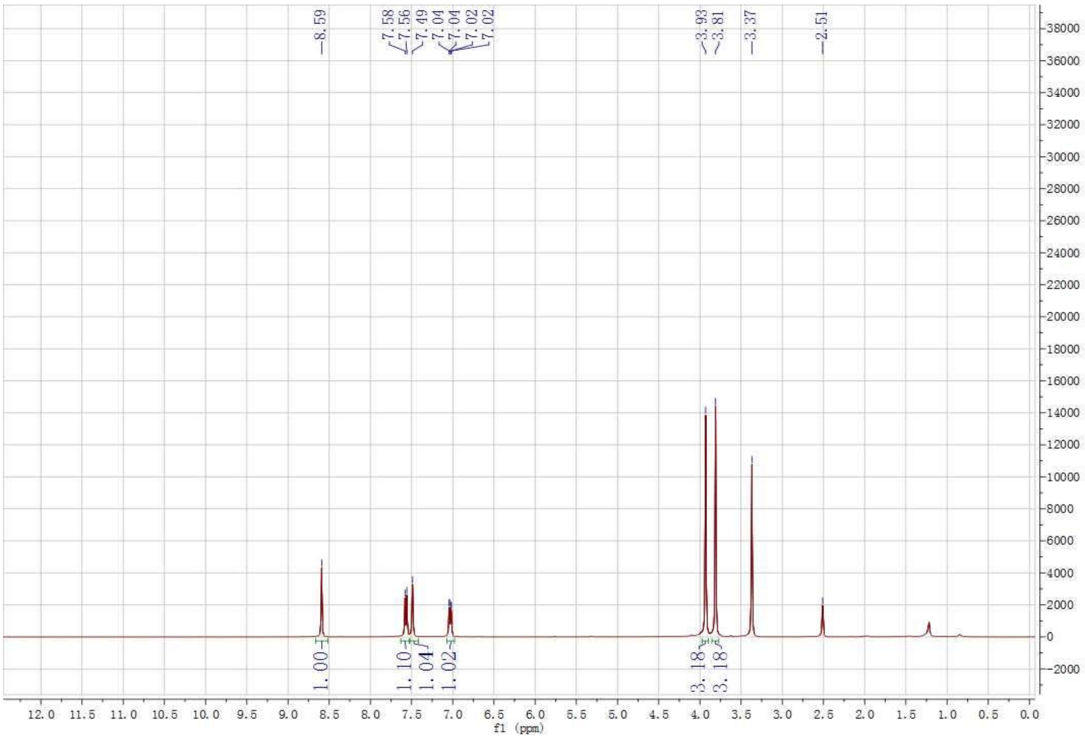

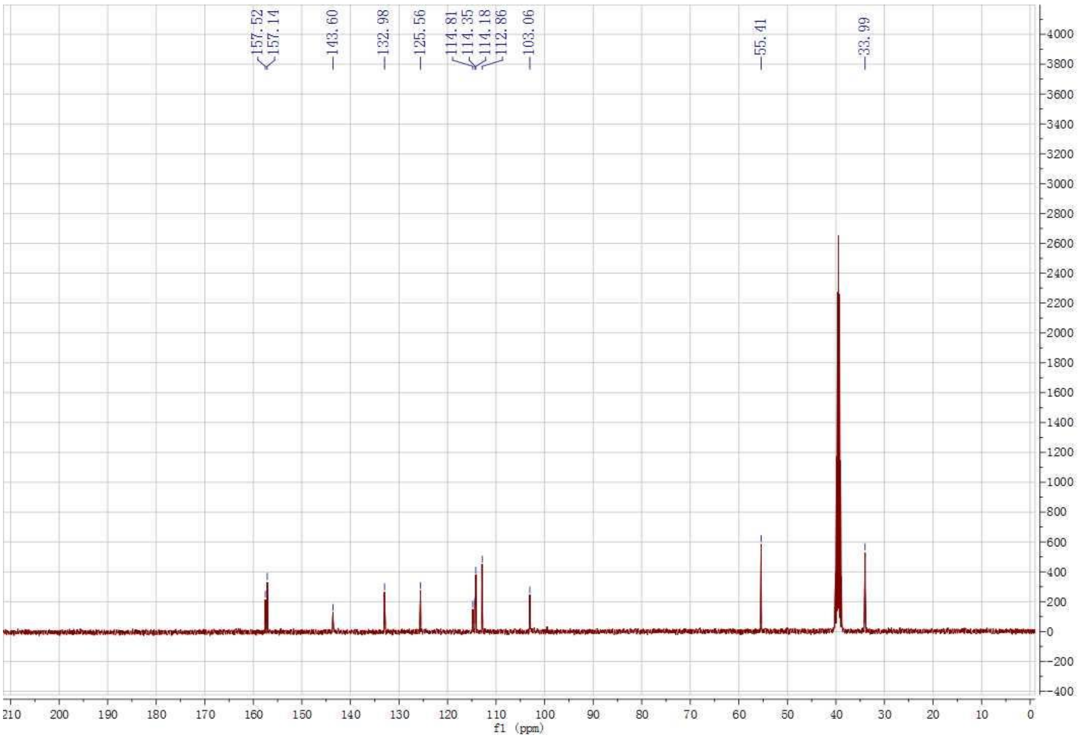

Supplementary Figure 66. ^{1}H and ^{13}C NMR spectrum for **5g**

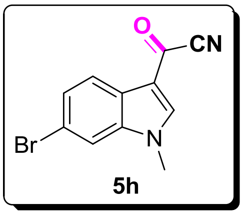

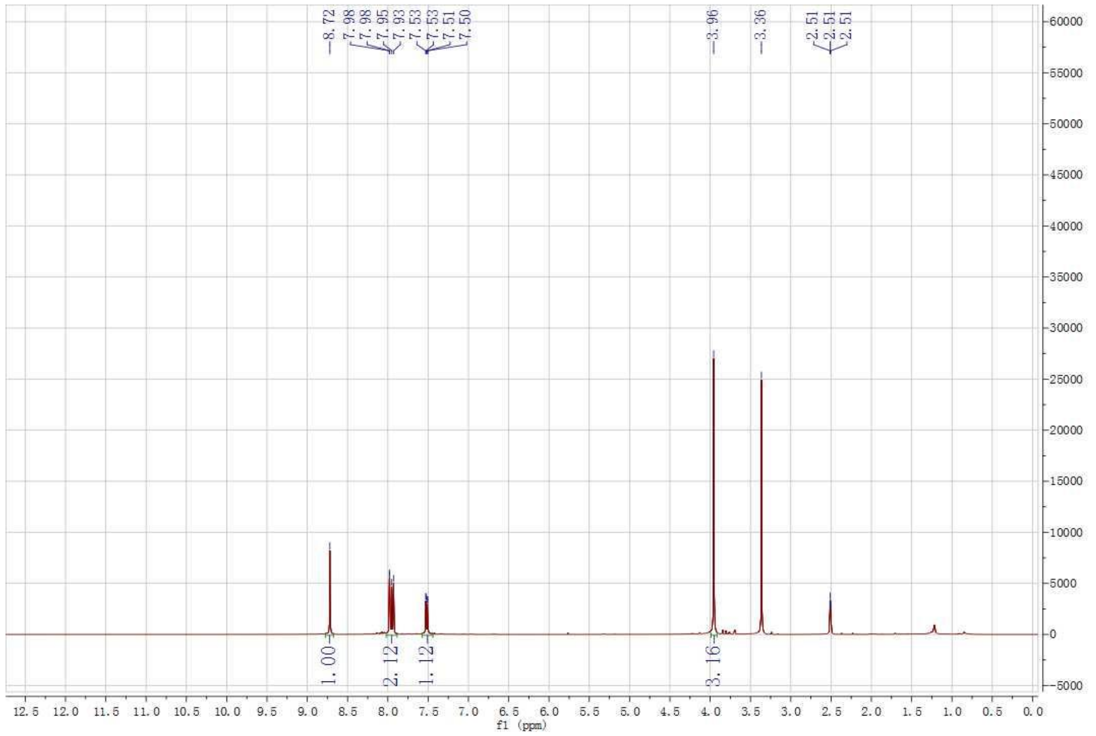

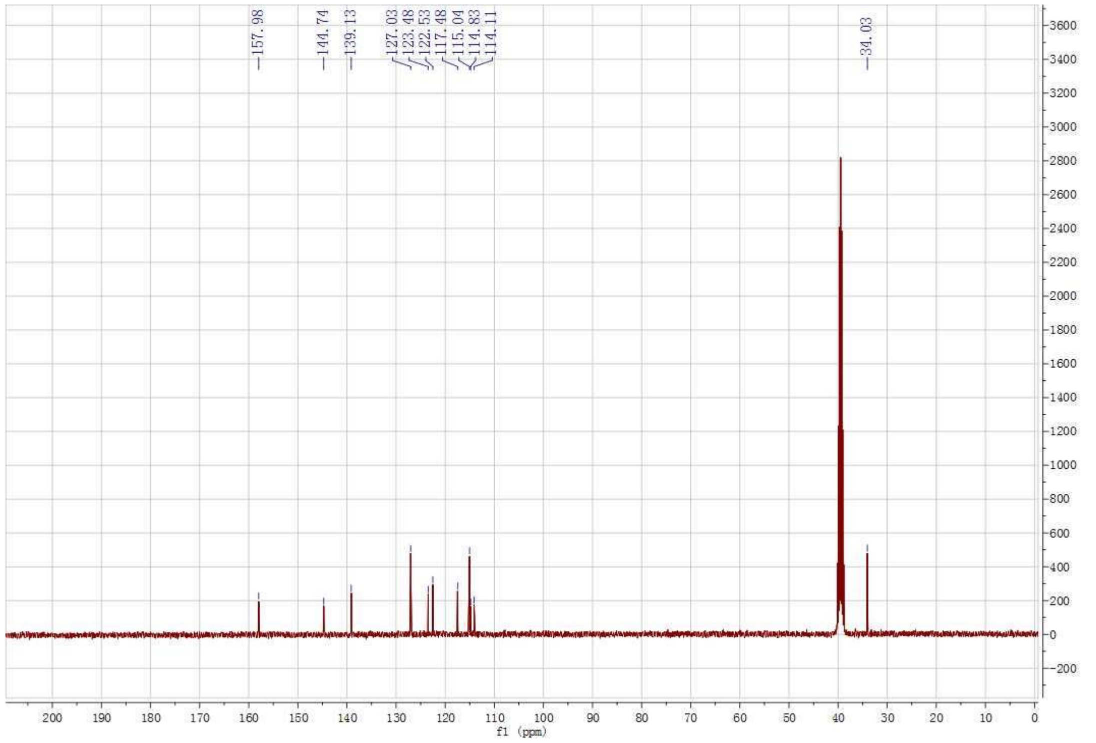

Supplementary Figure 67. ^{1}H and ^{13}C NMR spectrum for **5h**

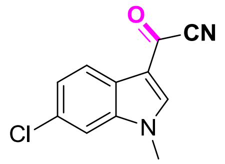

5i

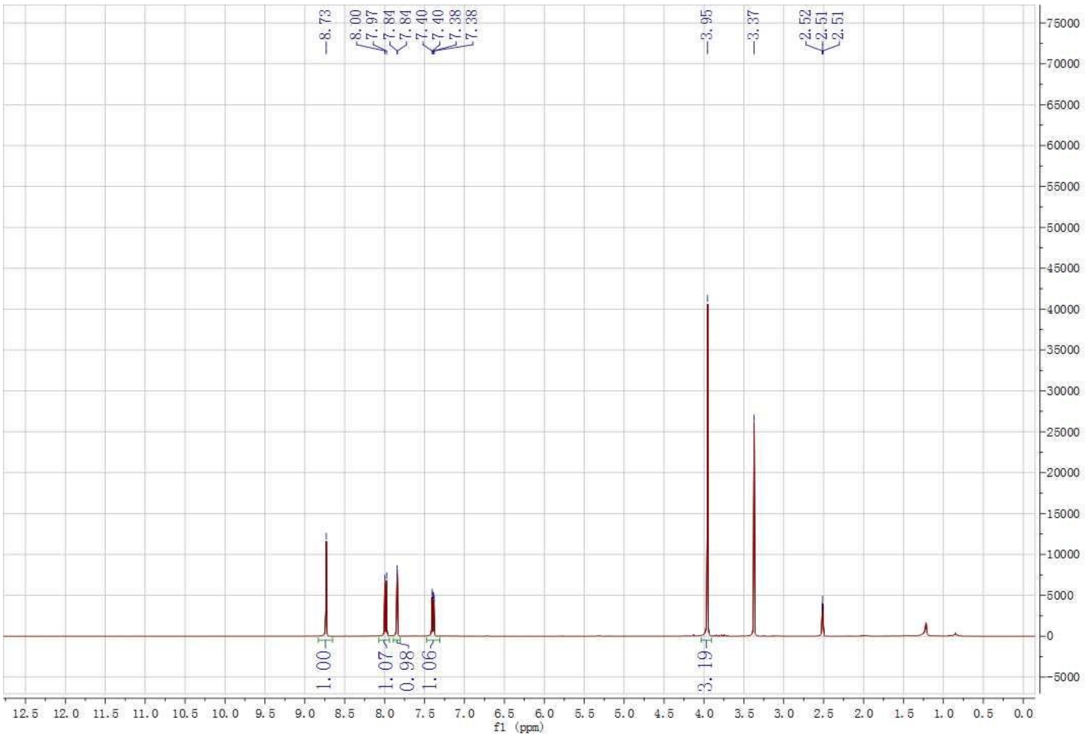

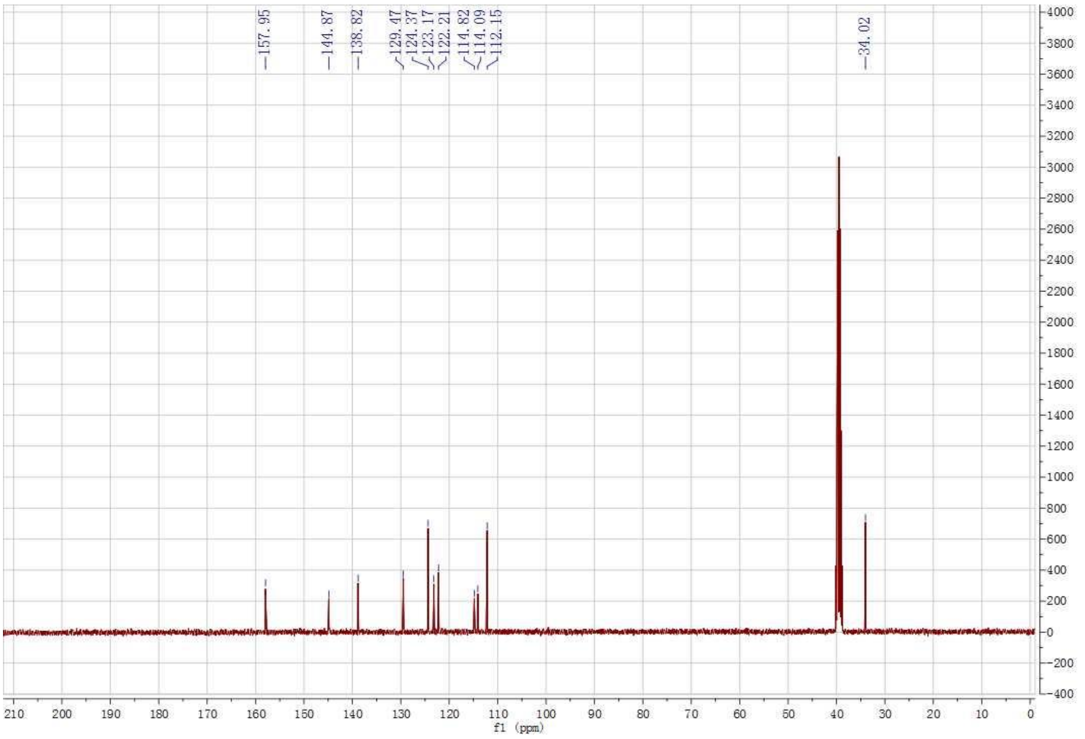

Supplementary Figure 68. ^{1}H and ^{13}C NMR spectrum for **5i**

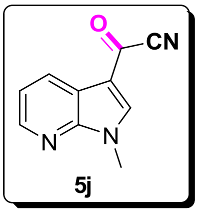

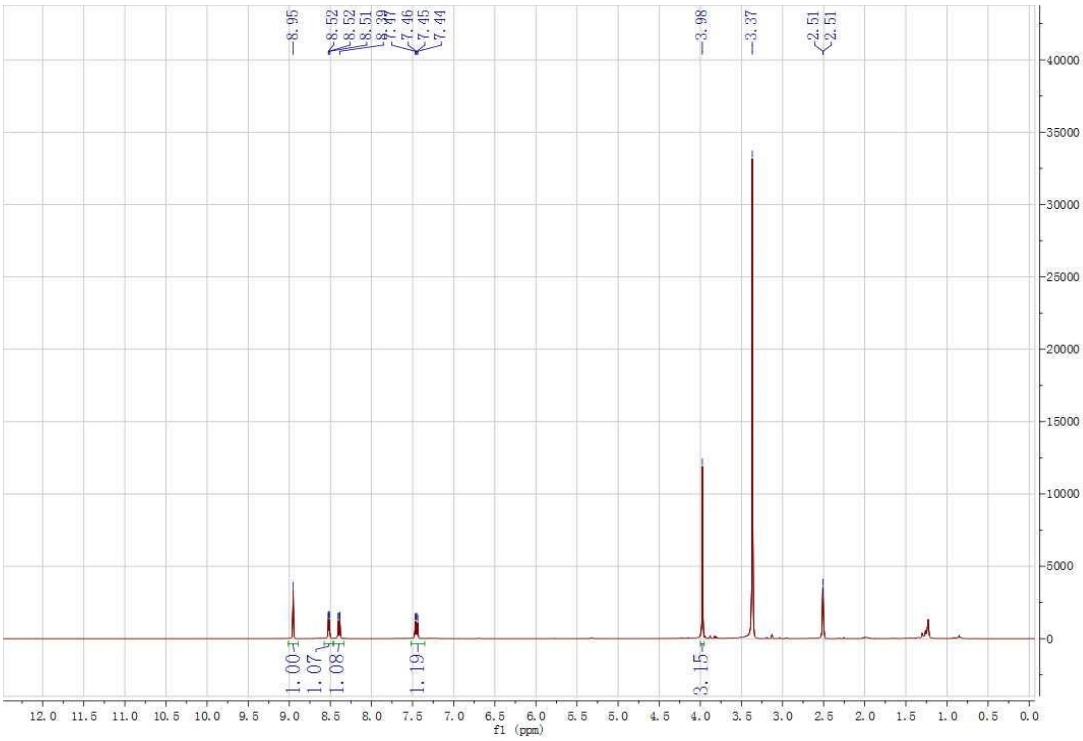

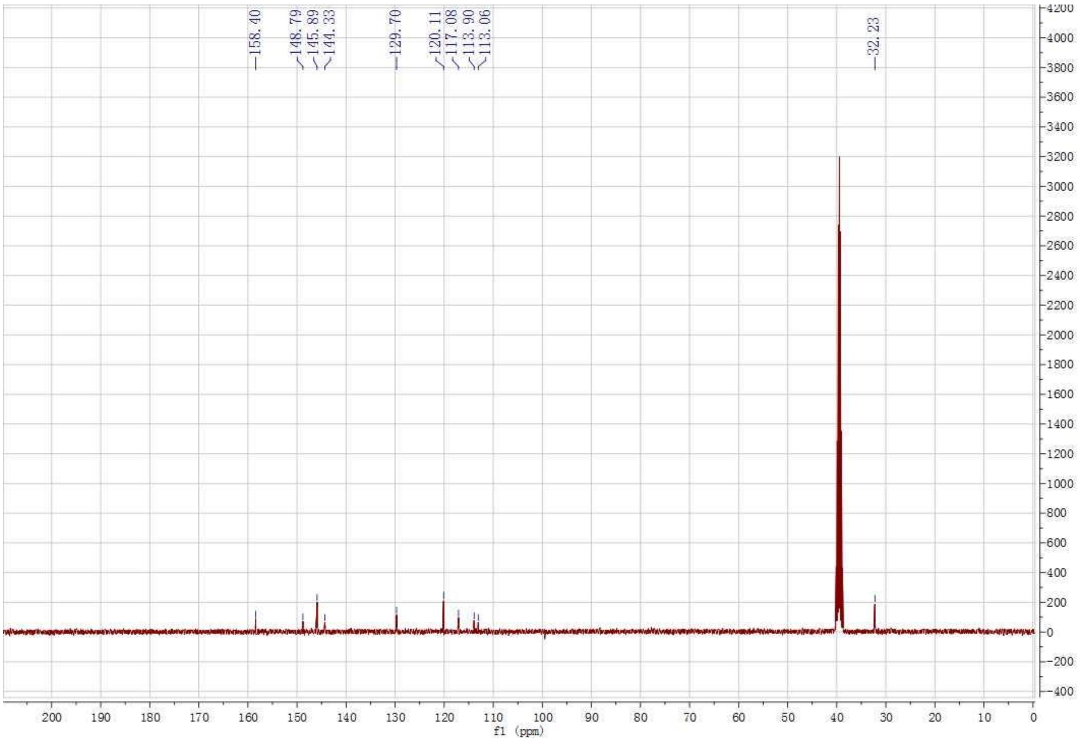

Supplementary Figure 69. ^{1}H and ^{13}C NMR spectrum for **5j**

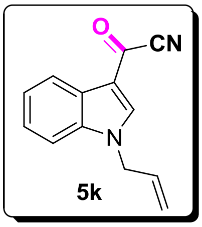

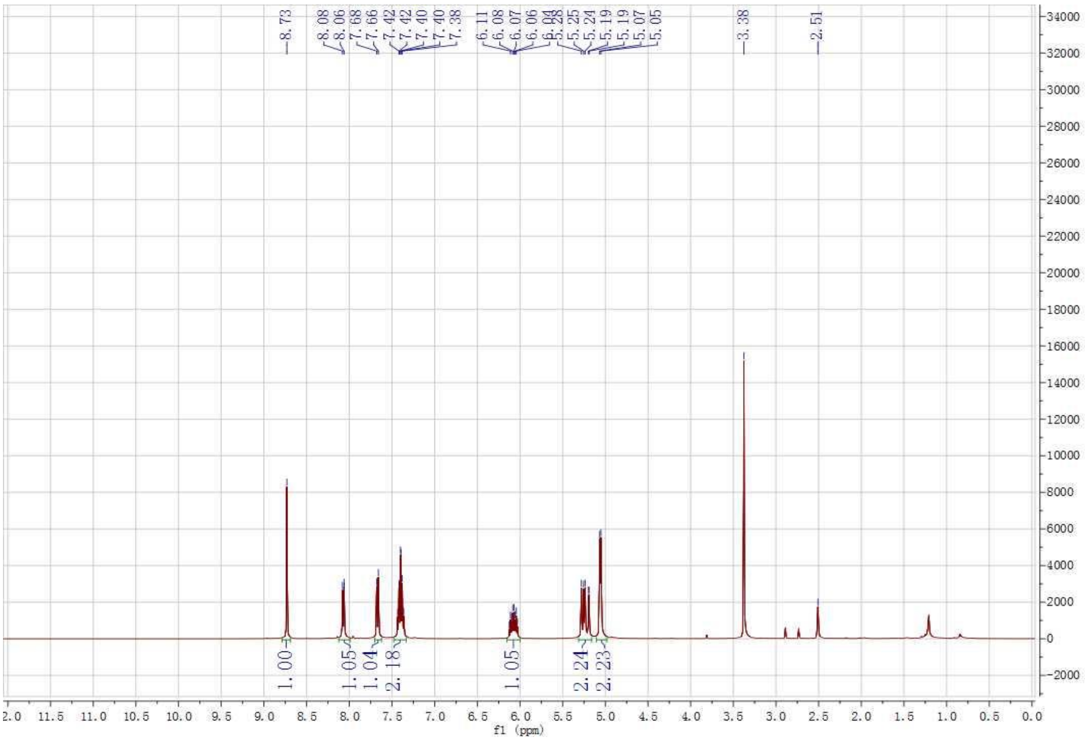

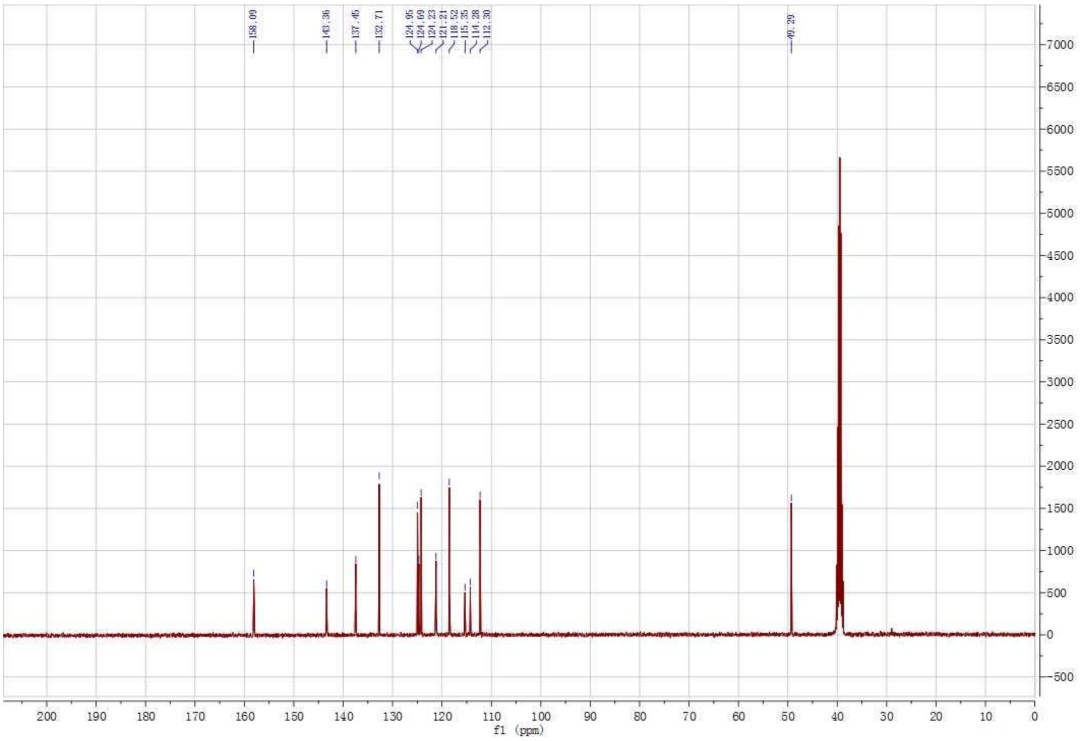

Supplementary Figure 70. ^{1}H and ^{13}C NMR spectrum for **5k**

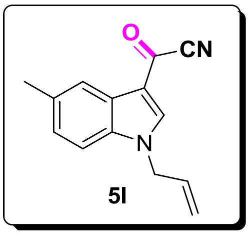

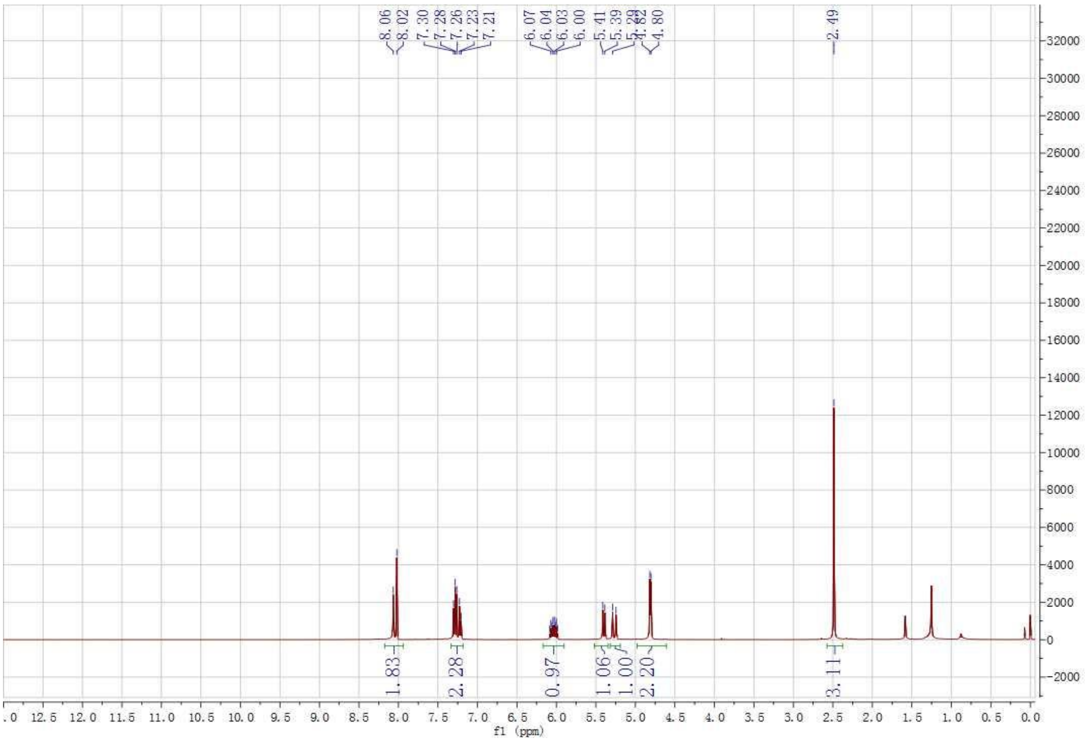

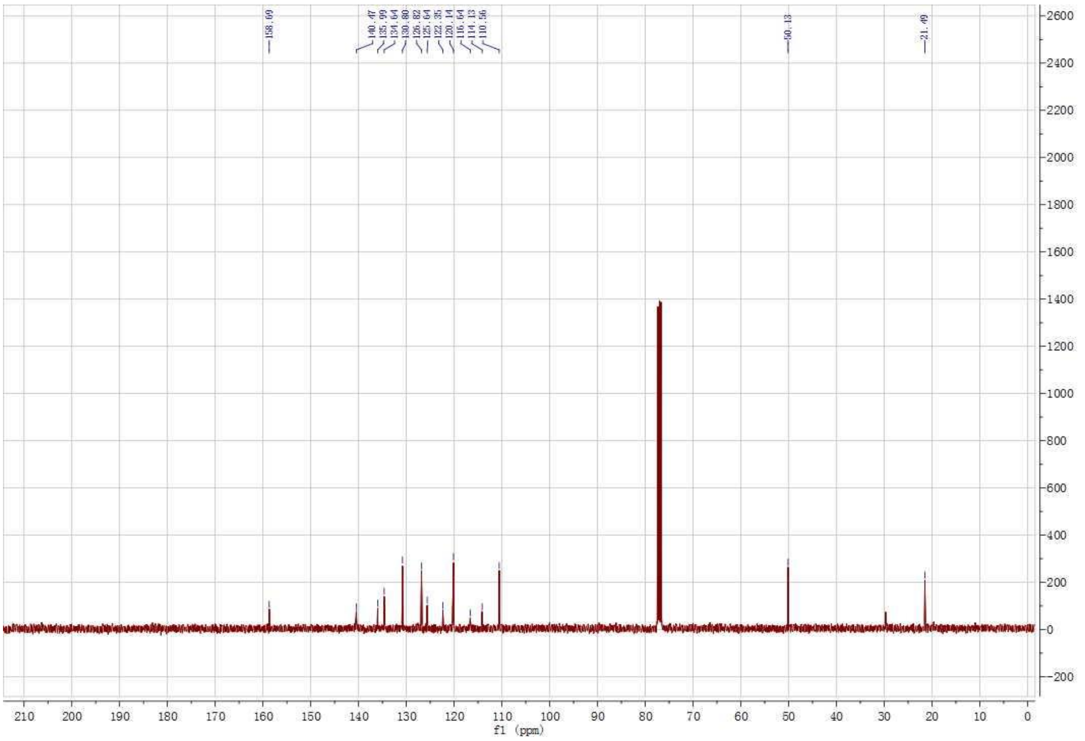

Supplementary Figure 71. ^{1}H and ^{13}C NMR spectrum for **5l**

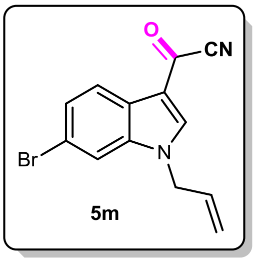

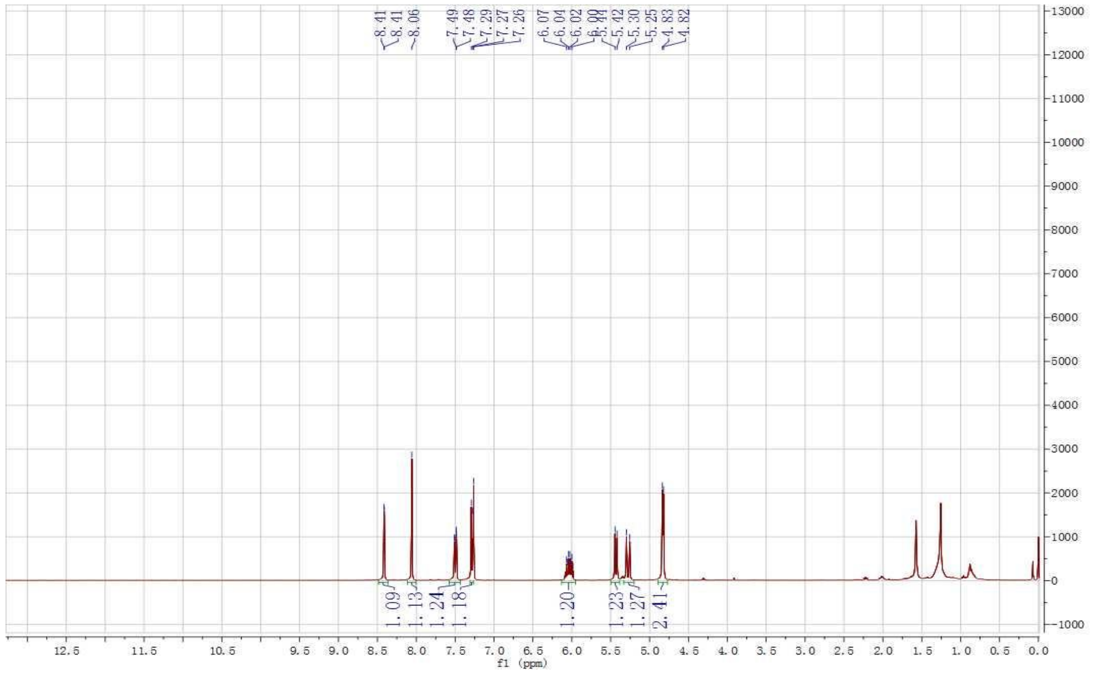

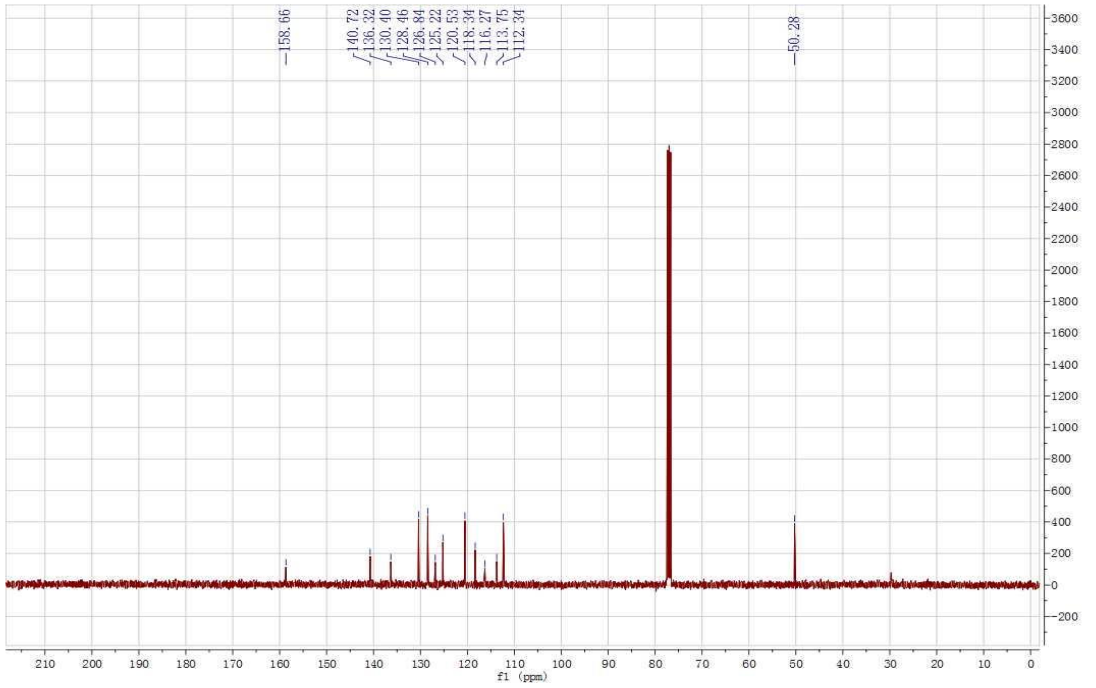

Supplementary Figure 72. ^{1}H and ^{13}C NMR spectrum for **5m**

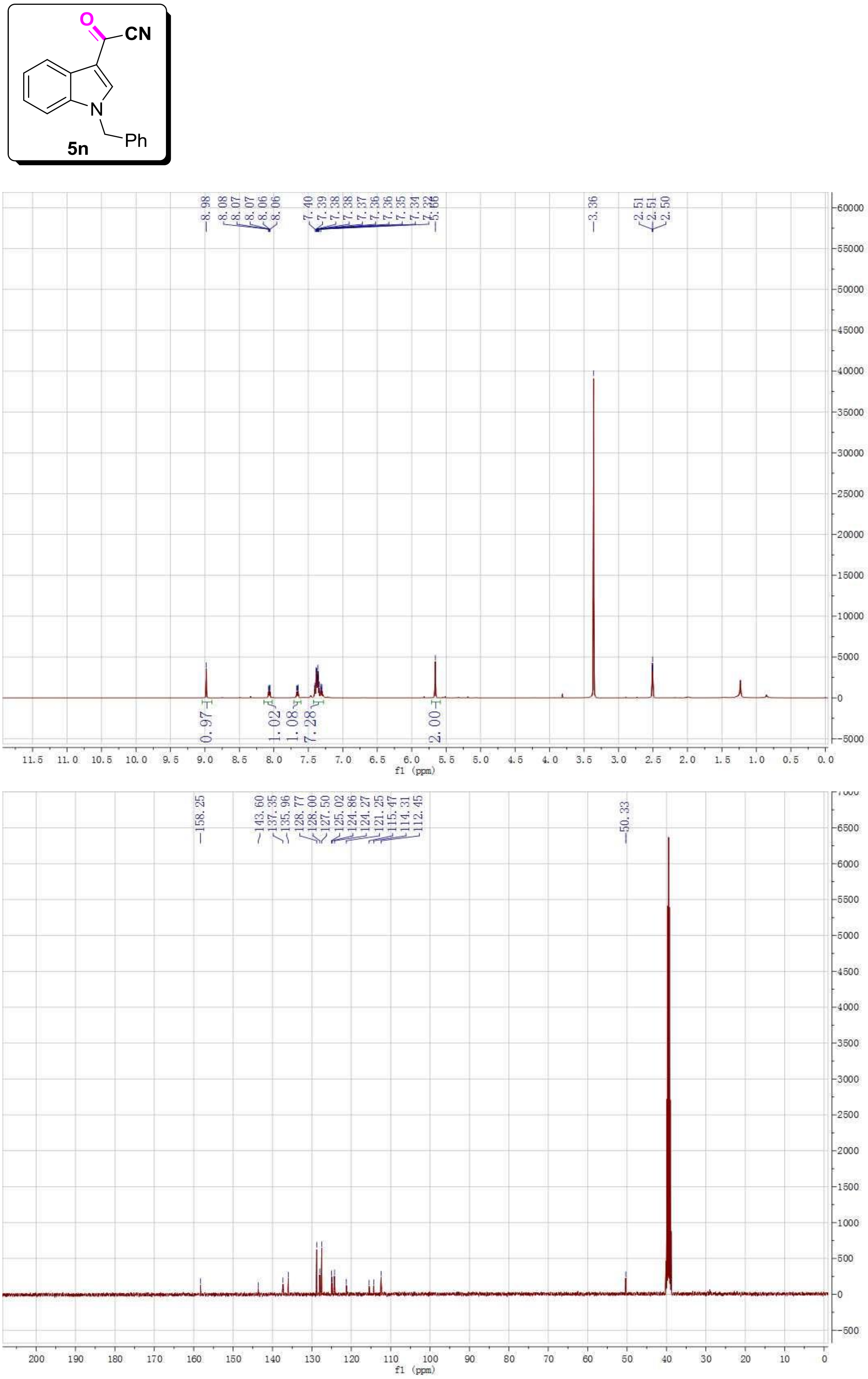

Supplementary Figure 73. ^{1}H and ^{13}C NMR spectrum for **5n**

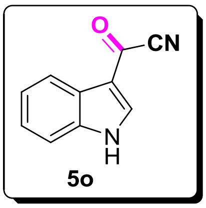

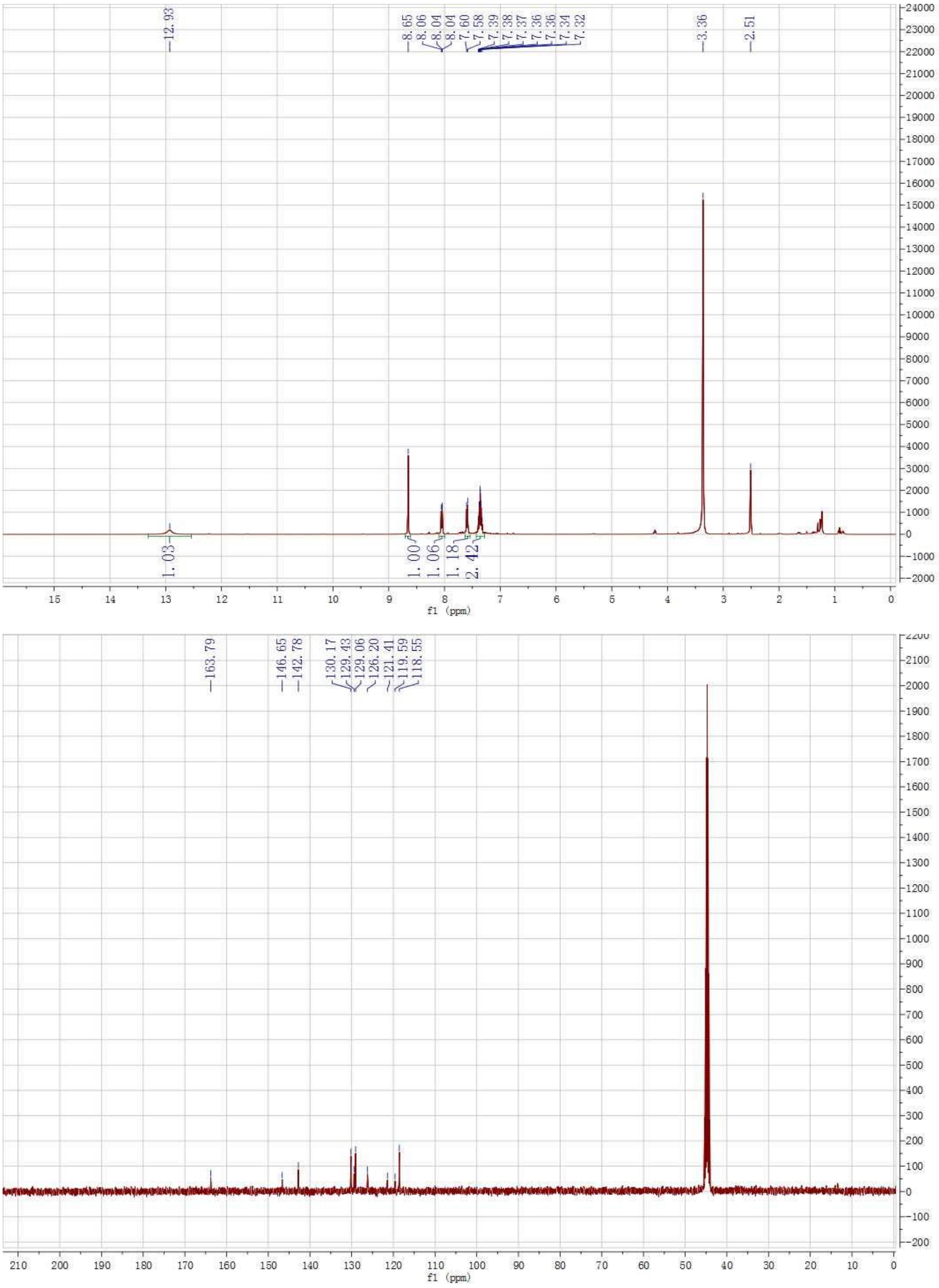

Supplementary Figure 74. ^{1}H and ^{13}C NMR spectrum for **5o**

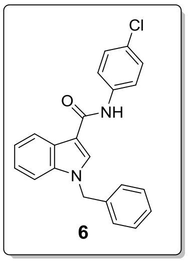

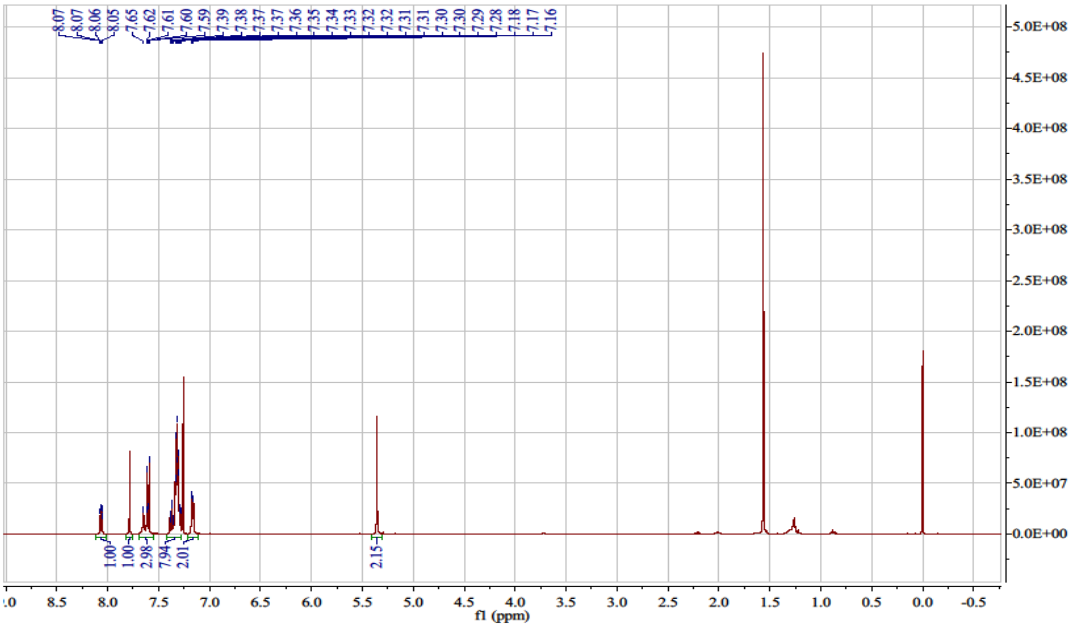

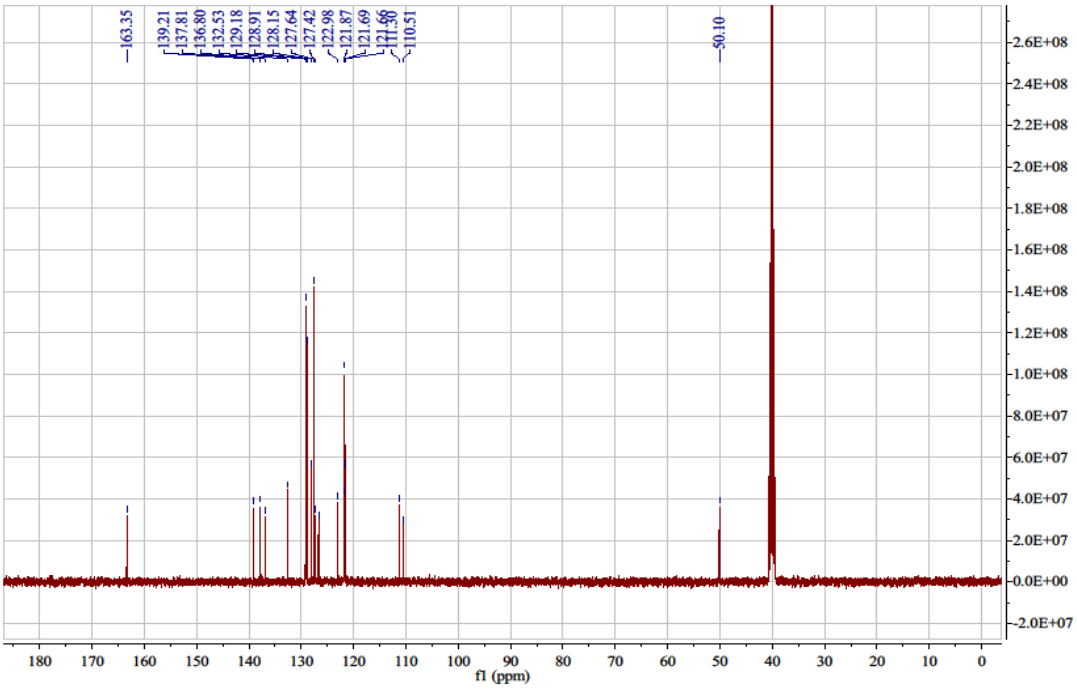

Supplementary Figure 75. ^{1}H and ^{13}C NMR spectrum for **6.**

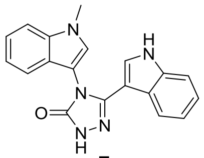

7c

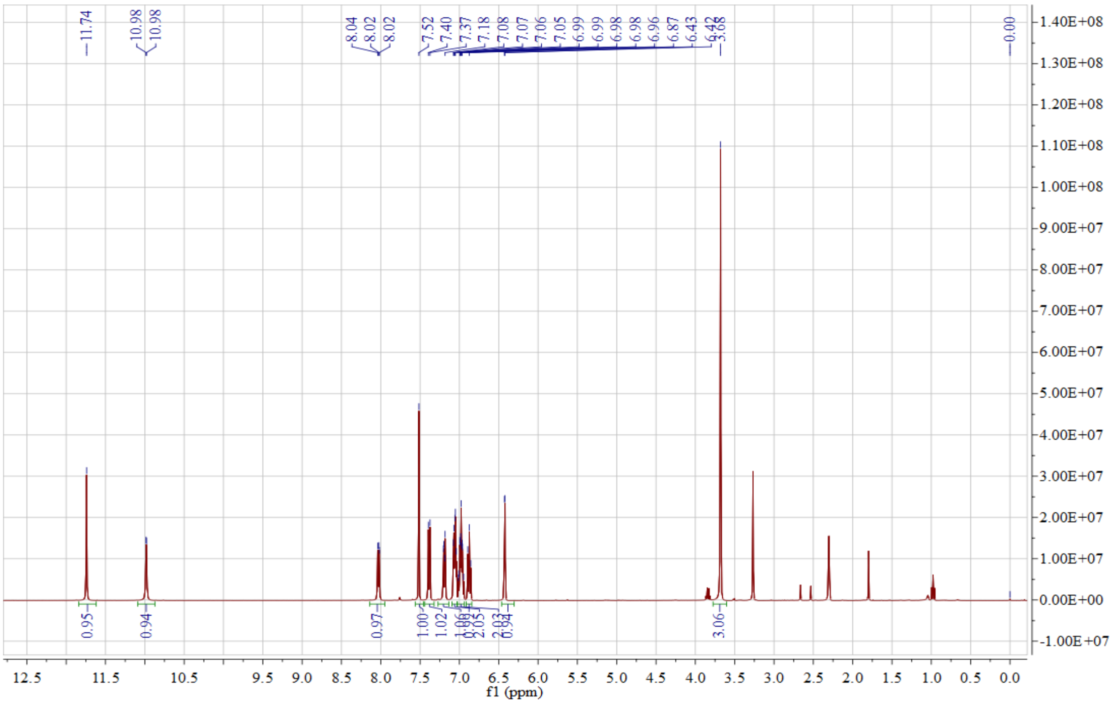

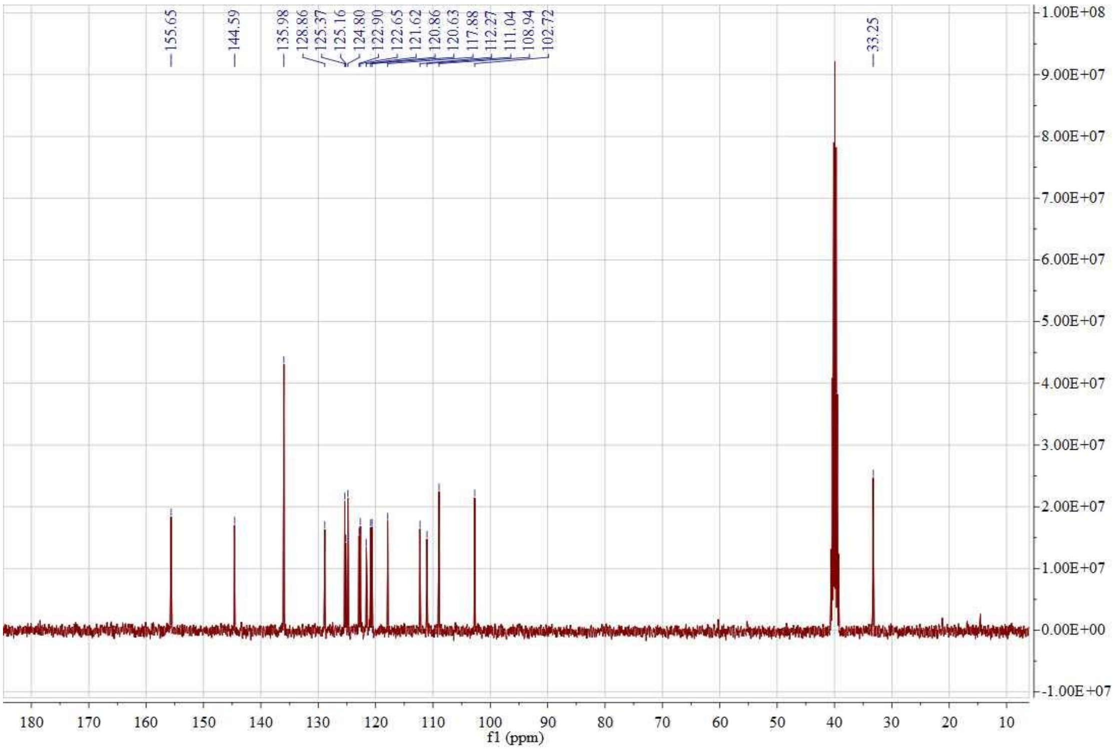

Supplementary Figure 76. ^{1}H and ^{13}C NMR spectrum for **7c.**

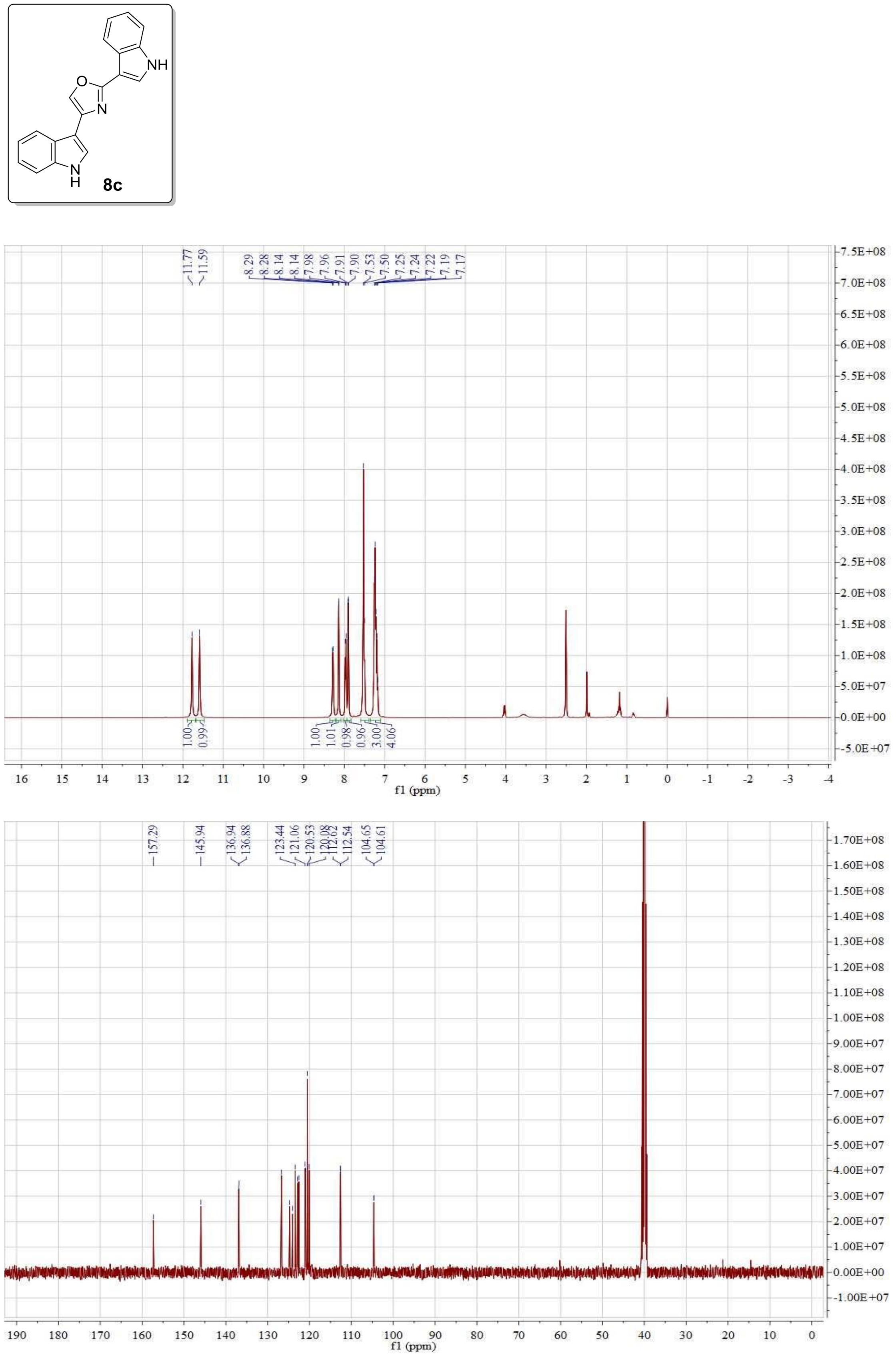

Supplementary Figure 77. ^{1}H and ^{13}C NMR spectrum for **8c.**

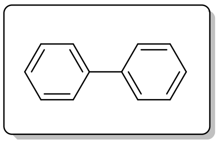

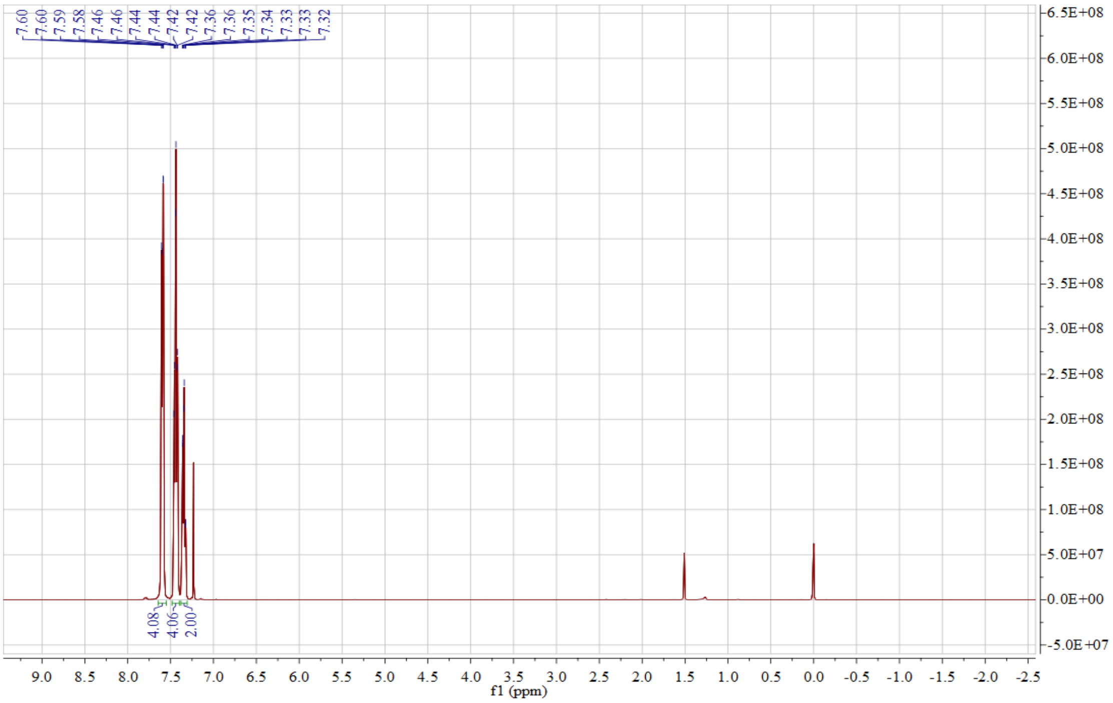

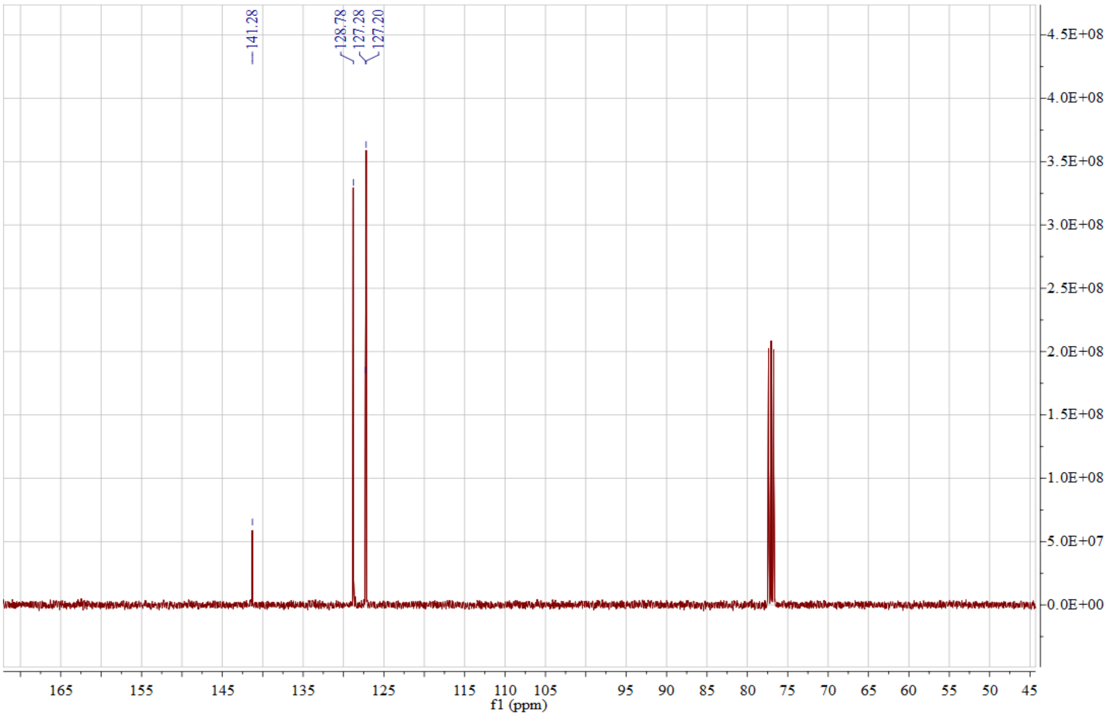

Supplementary Figure 78. ^{1}H and ^{13}C NMR spectrum for biphenyl.

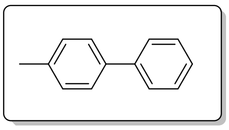

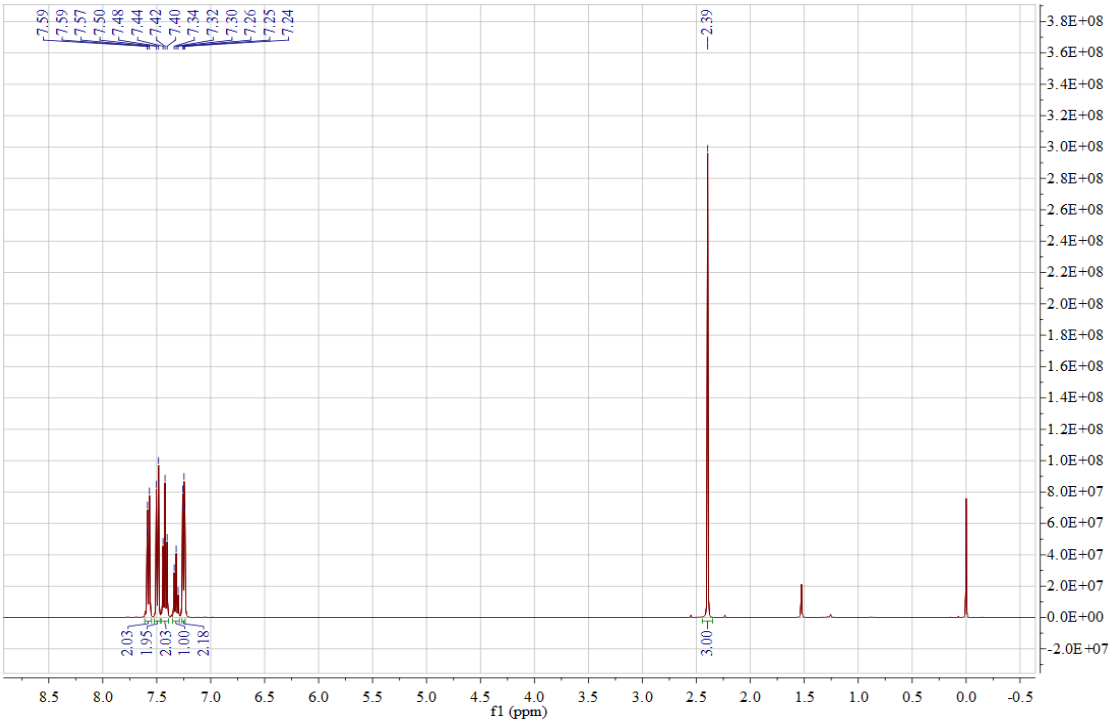

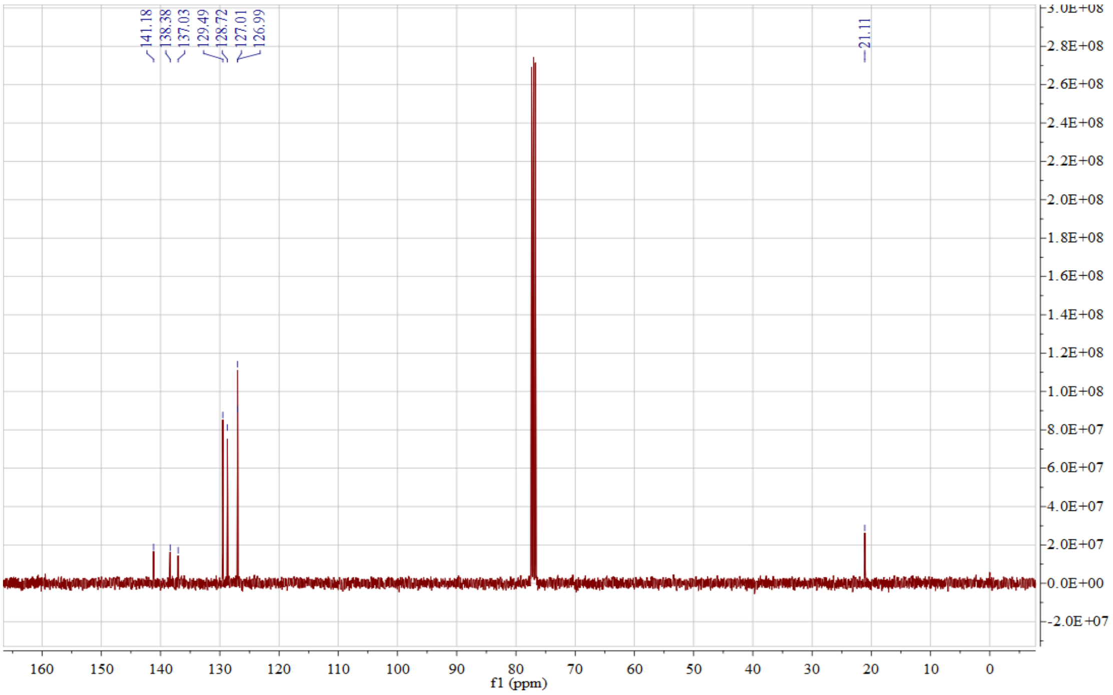

Supplementary Figure 79. ^{1}H and ^{13}C NMR spectrum for 4-methyl-1,1'-biphenyl.

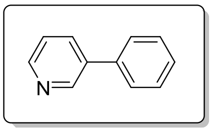

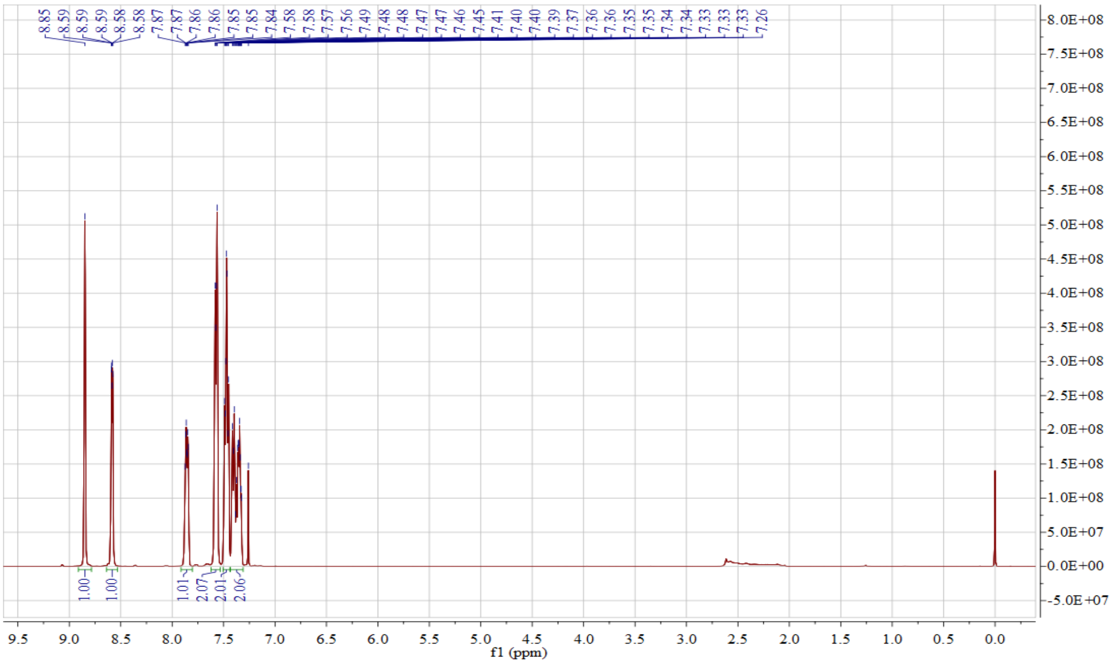

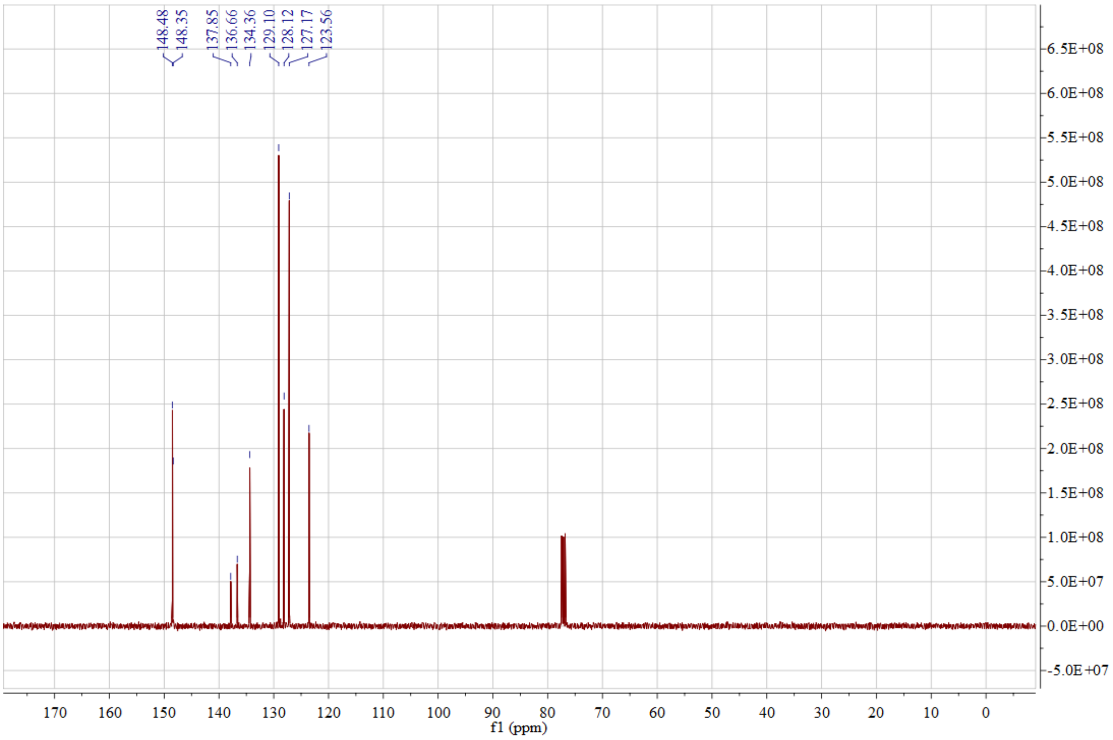

Supplementary Figure 80. ^{1}H and ^{13}C NMR spectrum for 3-phenylpyridine.

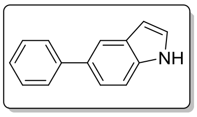

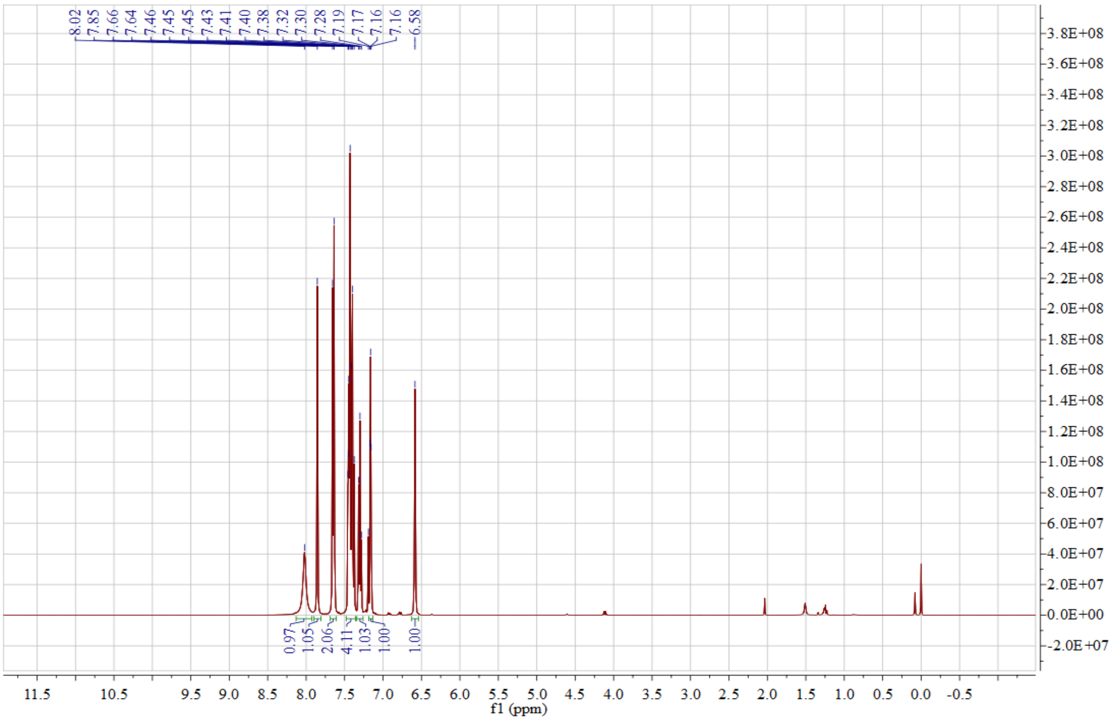

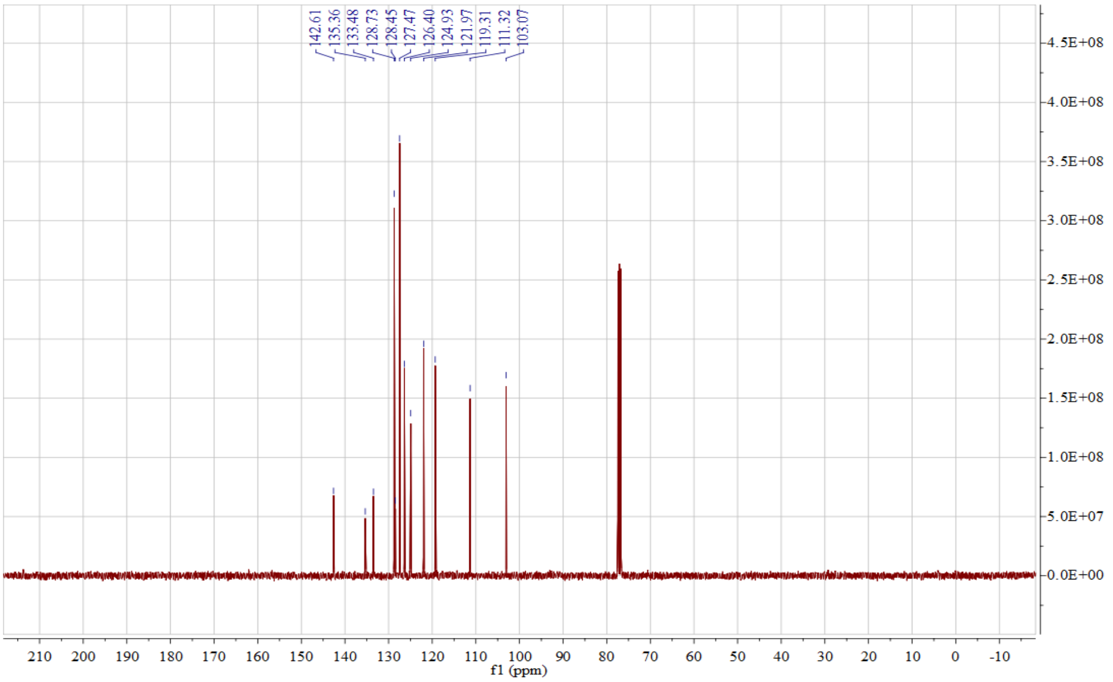

Supplementary Figure 81. ^{1}H and ^{13}C NMR spectrum for 5-phenyl-1H-indole.

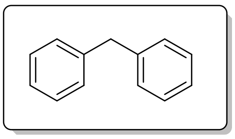

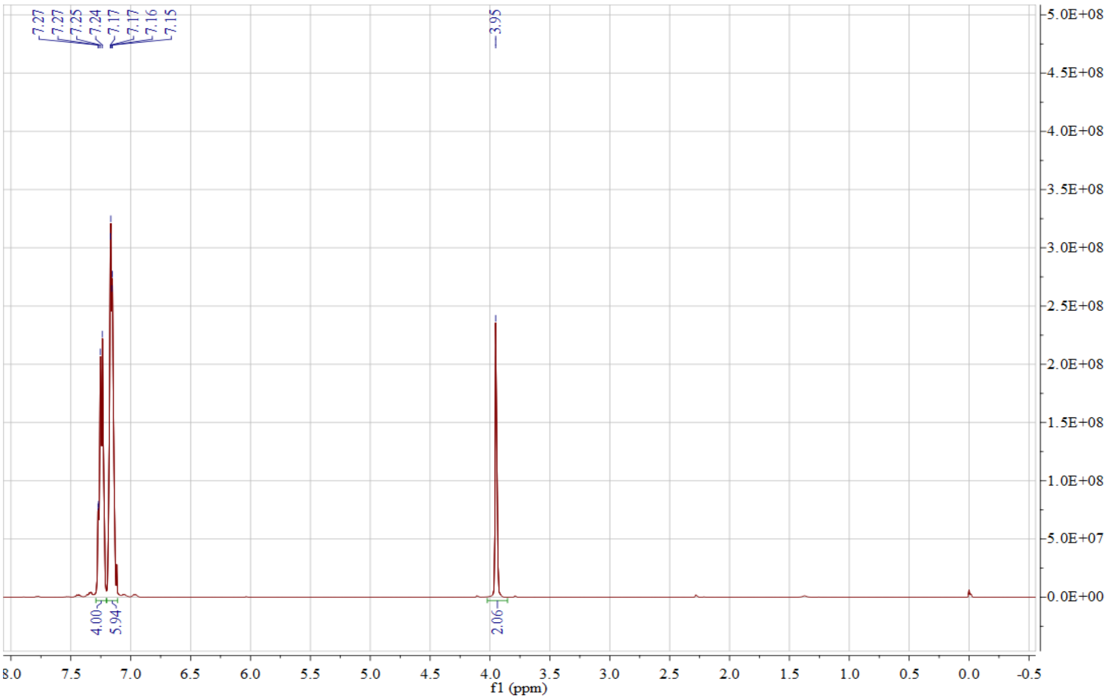

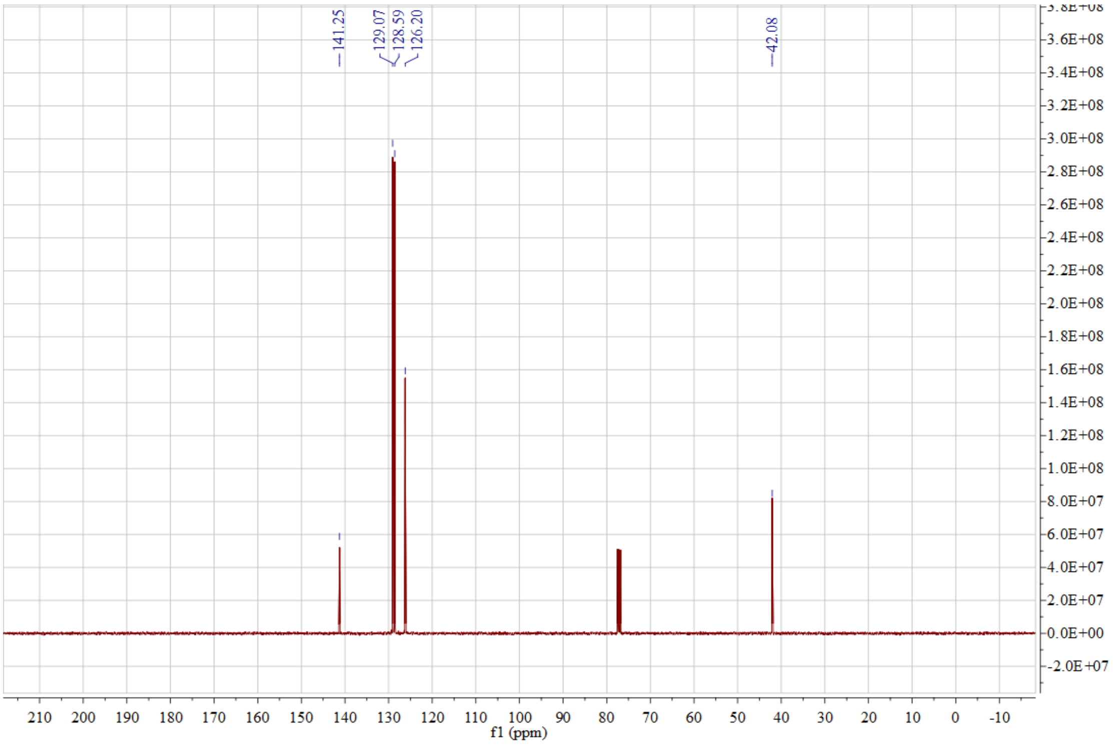

Supplementary Figure 82. ^{1}H and ^{13}C NMR spectrum for diphenylmethane.

Supplementary References

1. Koh K, Jeon M, Chevrier DM, Zhang P, Yoon CW, Asefa T. Novel Nanoporous N-Doped Carbon-Supported Ultrasmall Pd Nanoparticles: Efficient Catalysts for Hydrogen Storage and Release. *Appl. Catal. B: Environ.* **203**, 820-828 (2017).
2. Long X, Li Z, Gao G, Sun P, Wang J, Zhang B, Zhong J, Jiang Z, Li F. Graphitic phosphorus coordinated single Fe atoms for hydrogenative transformations. *Nature Commun.* **11**, 4074 (2020).
3. Patel MA, Luo F, Khoshi MR, Rabie E, Zhang Q, Flach CR, Mendelsohn R, Garfunkel E, Szostak M, He H. P-Doped Porous Carbon as Metal Free Catalysts for Selective Aerobic Oxidation with an Unexpected Mechanism. *ACS Nano* **10**, 2305-2315 (2016).
4. Cheng T, Chen J, Cai A, Wang J, Liu H, Hu Y, Bao X, Yuan P. Synthesis of Pd/SiO_2 Catalysts in Various HCl Concentrations for Selective NBR Hydrogenation: Effects of H^+ and Cl^- Concentrations and Electrostatic Interactions. *ACS Omega* **3**, 6651-6659 (2018).
5. Li B, Guan Z, Wang W, Yang X, Hu J, Tan B, Li T. Highly Dispersed Pd Catalyst Locked in Knitting Aryl Network Polymers for Suzuki–Miyaura Coupling Reactions of Aryl Chlorides in Aqueous Media. *Adv. Mater.* **24**, 3390-3395 (2012).
6. Wang W, Zheng A, Zhao P, Xia C, Li F. Au-NHC@Porous Organic Polymers: Synthetic Control and Its Catalytic Application in Alkyne Hydration Reactions. *ACS Catal.* **4**, 321-327 (2014).
7. Bukhryakov KV, Mugemana C, Vu KB, Rodionov VO. Palladium N-Heterocyclic Carbene Precatalyst Site Isolated in the Core of a Star Polymer. *Org. Lett.* **17**, 4826-4829 (2015).
8. Sashuk V, Schoeps D, Plenio H. Fluorophore Tagged Cross-Coupling Catalysts. *Chem. Commun.* 770-772 (2009).
9. Ogiwara, Y., Sakurai, Y., Hattori, H., Sakai, N. Palladium-Catalyzed Reductive Conversion of Acyl Fluorides via Ligand-Controlled Decarbonylation. *Org. Lett.* **20**, 4204-4208 (2018).
10. Agarwal, P. K., Dathi, M. D., Saifuddin, M., Kundu, B. Engineering of indole-based tethered biheterocyclic alkaloid meridianin into β-carboline-derived tetracyclic polyheterocycles via amino functionalization/6-endo cationic π-cyclization. *Beilstein J. Org. Chem.* **8**, 1901–1908 (2012).
11. Sauer, D. R., Kalvin, D., Phelan, K. M. Microwave-Assisted Synthesis Utilizing Supported Reagents: A Rapid and Efficient Acylation Procedure. *Org. Lett.* **5**, 4721-4724 (2003).
12. Reekie, T. A., Wilkinson, S. M., Law, V., Hibbs, D. E., Ong, J. A., Kassiou, M. Rapid access to N-(indol-2-yl)amides and N-(indol-3-yl)amides as unexplored pharmacophores. Org. Biomol. Chem. **15**, 576-580 (2017).
13. Abdel, R., Farghaly, A. H. Synthesis, Reactions and Antimicrobial Activity of Some New Indolyl-1,3,4-Oxadiazole, Triazole and Pyrazole Derivatives. *J. Chin. Chem. Soc.* **51**, 147-156 (2004).
14. Veale, Clinton G. L. A new synthesis of versatile indolyl-3-carbonylnitriles. *Tetrahedron Lett.* **56**, 5287-5290 (2015).
